# The role of neuroimaging in Parkinson’s disease

**DOI:** 10.1111/jnc.15516

**Published:** 2021-10-03

**Authors:** Natasha S. R. Bidesi, Ida Vang Andersen, Albert D. Windhorst, Vladimir Shalgunov, Matthias M. Herth

**Affiliations:** ^1^ Department of Drug Design and Pharmacology University of Copenhagen Copenhagen Denmark; ^2^ Radiology and Nuclear Medicine, Amsterdam UMC Vrije Universiteit Amsterdam Amsterdam Netherlands; ^3^ Department of Clinical Physiology Nuclear Medicine and PET, Rigshospitalet Copenhagen Denmark

**Keywords:** alpha‐synuclein, neurodegeneration, neuroimaging, Parkinson's disease, PET, SPECT

## Abstract

Parkinson's disease (PD) is a neurodegenerative disorder that affects millions of people worldwide. Two hallmarks of PD are the accumulation of alpha‐synuclein and the loss of dopaminergic neurons in the brain. There is no cure for PD, and all existing treatments focus on alleviating the symptoms. PD diagnosis is also based on the symptoms, such as abnormalities of movement, mood, and cognition observed in the patients. Molecular imaging methods such as magnetic resonance imaging (MRI), single‐photon emission computed tomography (SPECT), and positron emission tomography (PET) can detect objective alterations in the neurochemical machinery of the brain and help diagnose and study neurodegenerative diseases. This review addresses the application of functional MRI, PET, and SPECT in PD patients. We provide an overview of the imaging targets, discuss the rationale behind target selection, the agents (tracers) with which the imaging can be performed, and the main findings regarding each target's state in PD. Molecular imaging has proven itself effective in supporting clinical diagnosis of PD and has helped reveal that PD is a heterogeneous disorder, which has important implications for the development of future therapies. However, the application of molecular imaging for early diagnosis of PD or for differentiation between PD and atypical parkinsonisms has remained challenging. The final section of the review is dedicated to new imaging targets with which one can detect the PD‐related pathological changes upstream from dopaminergic degeneration. The foremost of those targets is alpha‐synuclein. We discuss the progress of tracer development achieved so far and challenges on the path toward alpha‐synuclein imaging in humans.

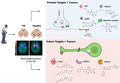

## Graphical abstract

This review addresses the clinical application of functional MRI, PET, and SPECT imaging in Parkinson's disease (PD). We provide an overview of the imaging targets and agents (tracers) used in PD diagnosis and clinical research and discuss the main findings for each target. Molecular imaging has proven itself effective in supporting symptom‐based clinical diagnosis of PD, but early pre‐symptomatic diagnosis and differentiation between PD and atypical parkinsonisms have remained challenging. New imaging targets capable of revealing PD‐related pathological changes upstream from dopaminergic degeneration should help overcome these challenges. The foremost of those targets is alpha‐synuclein.

**Abbreviations**
[^11^C/^18^F]DTBZ: [^11^C](+)dihydrotetrabenazine/[^18^F](+)dihydrotetrabenazine
[^11^C]HED: [^11^C]meta‐hydroxyephedrine
[^11^C]MeNER: (S,S)‐[^11^C]‐2‐(α‐(2‐methoxyphenoxy)benzyl)morpholine
[^11^C]MP4A: [^11^C]methyl‐4‐piperidyl acetate
[^11^C]PMP: [^11^C]methyl‐4‐piperidyl propionate
[^11^C]TMSX: 7‐methyl‐[^11^C]‐(E )‐8‐(3,4,5‐trimethoxystyryl)1,3,7‐trimethylxanthine
[^123^I]5‐IA‐85380: [^123^I]5‐iodo‐3‐[2(S)‐2‐azetidinylmethoxy]pyridine
[^123^I]IBVM: [^123^I]iodobenzovesamicol
[^123^I]MIBG: [^123^I]*meta*‐iodobenzylguanidine
[^18^F]BCPP‐EF: 2‐*tert*‐butyl‐4‐chloro‐5‐{6‐[2‐(2‐^18^F‐fluoroethoxy)‐ethoxy]‐pyridin‐3‐ylmethoxy}‐2*H*‐pyridazin‐3‐one
2‐[^18^F]fluoro‐A‐85380: 2‐[^18^F]fluoro‐3‐[2(S)‐2‐azetidinylmethoxy]pyridine
[^18^F]FDA: [^18^F]fluorodopamine
[^18^F]FDG: [^18^F]fluorodeoxyglucose
[^18^F]F‐DOPA: 6‐[^18^F]fluoro‐DOPA
[^18^F]FEOBV: [^18^F]fluoroethoxy benzovesamicol
[^18^F]FEP‐4MA: N‐[^18^F]fluoroethylpiperidin‐4‐ylmethyl acetate
[^18^F]FMT: 6‐[^18^F]fluoro‐m‐tyrosine
[^99m^Tc]Tc‐HMPAO: [^99m^Tc]hexamethylpropylene amine oxime
[^99m^Tc]Tc‐ECD: [^99m^Tc]‐ethyl cysteinate dimer
3‐MT: 3‐methoxytyramine
5‐HTR: 5‐hydroxytryptamine receptors, serotonin receptors
α‐Syn: alpha‐synuclein
A_2A_R: adenosine A_2A_ receptors
AADC: aromatic L‐amino acid decarboxylase
AChE: acetylcholine esterase
AD: Alzheimer's disease
ALDH: aldehyde dehydrogenase
APP: amyloid precursor protein
ASL: arterial spin labeling
BBB: blood–brain barrier
BOLD: blood oxygenation level dependent
cAMP: cyclic adenosine monophosphate
CB1: type 1 cannabinoid receptors
cGMP: cyclic guanosine monophosphate
COMT: catechol‐O‐methyl transferase
DAT: dopamine transporter
DBS: deep brain stimulation
DJ‐1: DJ‐1 mitochondrial protein
DLB: dementia with Lewy bodies
DOPAL: 3,4‐dihydroxyphenylacetaldehyde
ET: essential tremor
(f)MRI: (functional) magnetic resonance imaging
GPCR: G protein coupled receptor
HDAC: histone deacetylase
HVA: homovanillic acid
LC: locus coeruleus
L‐DOPA: L‐3,4‐dihydroxyphenylalanine
LID: L‐DOPA‐induced dyskinesia
LRRK2: leucine‐rich repeat kinase 2
MAO‐B: monoamine oxidase‐B
MPTP: 1‐methyl‐4‐phenyl‐1,2,3,6‐tetrahydropyridine
MSA: multiple system atrophy
NET: norepinephrine transporter
NMDAR: *N*‐methyl‐d‐aspartate receptor
PD: Parkinson's disease
PDE: phosphodiesterase
PDRP: PD‐related metabolic patterns
PET: positron emission tomography
PINK1: phosphatase and tensin homolog‐induced putative kinase 1
PRKN: Parkin E3 ubiquitin ligase
PS: Parkinsonian syndromes
PSP: progressive supranuclear palsy
ROS: reactive oxygen species
SERT: serotonin transporters
SN: substantia nigra
SNCA: a‐synuclein gene
SPECT: single photon emission computed tomography
SV2A: synaptic vesicle protein 2A
TSPO: translocator protein
UCHL1: ubiquitin carboxyl‐terminal hydrolase‐1
VAChT: vesicular acetylcholine transporter
VMAT2: vesicular monoamine transporter 2

## 1 INTRODUCTION

### 1.1 Parkinson's disease and its impact on society

Parkinson's disease (PD) is the second most common neurodegenerative disorder. It currently affects 0.2% of the average global population, 1% of the population older than 60 years, and up to 4% of people above 80 years, indicating an exponential growth of prevalence with age (Driver et al., 2009). In the aging Western society, the prevalence of PD is expected to continue growing (Wanneveich et al., 2018), which will increase the financial burden on society due to high requirements of health care for PD patients (Gustavsson et al., 2011). PD is widely known for its motor symptoms such as tremors, rigidity, and inability to initiate movements, but PD patients also suffer from non‐motor symptoms such as mood disorders, cognitive impairment, sleep disorders, gastrointestinal symptoms, and pain (Antonini et al., 2018; Balestrino & Schapira, 2020; Mertsalmi et al., 2017). Combinations of observed motor and non‐motor symptoms can be very different between patients (Von Coelln and Shulman 2016). Right now, there is no cure available for PD, and all existing therapeutic approaches focus on relieving the symptoms (Oertel & Schulz, 2016).

Diagnosis of PD is based on patient history and clinical examination (Armstrong & Okun, 2020). Namely, motor symptoms (bradykinesia, tremor, postural instability) and response to conventional anti‐parkinsonic medication (see Section 1.3) have to be shown, while possible causes of secondary parkinsonism (e.g., head injuries or exposure to toxic agents) have to be excluded (Marsili et al., 2018). This poses problems, as unambiguous signs and symptoms of PD only appear at an advanced stage of the disease. Besides, the symptoms of PD overlap with essential tremor (ET) and “atypical” Parkinsonian syndromes (PS) such as dementia with Lewy bodies (DLB), multiple system atrophy (MSA), and progressive supranuclear palsy (PSP), which occur more frequently in old age and thus confound PD diagnosis in elderly patients (Rizzo et al., 2016). For definitive diagnosis, pathological changes in brain tissue have to be confirmed (Dickson et al., 2009). Emergence of non‐invasive molecular imaging methods such as magnetic resonance imaging (MRI), single photon emission computed tomography (SPECT) and positron emission tomography (PET) has made it possible to distinguish between PD and ET (and, in some cases, atypical PS) in living patients rather than based on postmortem examinations (Abbasi Gharibkandi and Hosseinimehr 2019; Saeed et al., 2020). However, the true potential of molecular imaging in PD lies in the discovery of biomarkers and targets to, respectively, diagnose PD before clinical symptoms appear and treat the cause, rather than just the symptoms, of the disease.

In this paper, we provide an overview of established and emerging targets and agents used for molecular imaging in PD patients and discuss the advances in the understanding of PD achieved through molecular imaging.

### 1.2 Known pathophysiological mechanisms of Parkinson's disease

Two major hallmarks of PD pathophysiology are the accumulation of misfolded alpha‐synuclein (α‐Syn) and decline of dopaminergic neurons in the substantia nigra (SN). This interferes with the signal transduction pathways in the brain, leading to symptoms of PD. Misfolding and accumulation of α‐Syn, abnormal dopamine metabolism, oxidative stress, and neuronal death mutually reinforce each other as the disease progresses (Dauer & Przedborski, 2003).

#### 1.2.1 Alpha synuclein

The function of α‐Syn is not fully understood, but it is involved in synaptic maintenance, including the regulation of dopamine vesicle size, dopamine transporter (DAT) localization and dopamine biosynthesis (Dauer & Przedborski, 2003; Meiser et al., 2013). The protein is localized near synaptic membranes and in the cytosol and has been found in different areas of the brain: SN, hippocampus, neocortex, hypothalamus, thalamus, and cerebellum (Lee et al., 2011).

In a healthy brain, α‐Syn exists as a monomeric intrinsically disordered protein (Fakhree et al., 2018). The development of PD is associated with the misfolding and aggregation of α‐syn monomers, leading to the formation of pathological oligomers and fibrils inside neurons (Kalia et al., 2013; Nors Pedersen et al., 2015). The accumulation of α‐Syn is also found in DLB, MSA, and in Alzheimer's disease (AD) (Kim et al., 2014; Schulz‐Schaeffer, 2010). The fibrillation process can be separated into the nucleation (lag) phase of proto‐fibril formation, followed by an exponential buildup of fibrils in the growth phase until nearly all monomers/proto‐fibrils are converted into fibrils (the plateau phase). It is a reversible process, where monomers are added and fall off even after the fibril load has reached the plateau (Iadanza et al., 2018).

The mechanism of α‐Syn transformation from a disordered monomer to ordered beta‐sheeted fibrils is still not fully understood (Brundin et al., 2017; Chatani & Yamamoto, 2018; Iadanza et al., 2018). In particular, it is not known where the process of α‐syn fibrillation starts. Originally, α‐syn pathology was postulated to start in the enteric nervous system and subsequently spread through the sympathetic and vagal connections to the brain and rest of the body (Braak et al., 2003; Goedert et al., 2013; Scheperjans et al., 2018). A newer perspective, corroborated by imaging studies, states that there are two major subtypes of PD: the “brain‐first” subtype, where the α‐syn pathology starts in the brain, and the “body‐first” subtype, where it starts in the gut (Horsager et al., 2020).

Independently of where α‐Syn aggregation starts, the propagation of this process in a prion‐like manner is responsible for the spreading of neurodegeneration across the brain (Angot et al., 2010; Stuendl et al., 2016; Vaquer‐Alicea & Diamond, 2019). Therefore, α‐Syn is the key molecule in the cascade leading to neuronal decline in PD.

#### 1.2.2 Dopamine

Dopamine is a catecholamine neurotransmitter, which comprises 80% of catecholamine content in the brain. In the central nervous system, dopamine is synthesized in SN, central tegmental area, and hypothalamus and is involved in movement control, learning and motivated behavior (Vallone et al., 2000). Dopamine exerts its function by binding to dopamine receptors, which all belong to the G protein coupled receptor (GPCR) superfamily and are separated into five subtypes (D_1_–D_5_) (Beaulieu et al., 2015).

**FIGURE 1:**
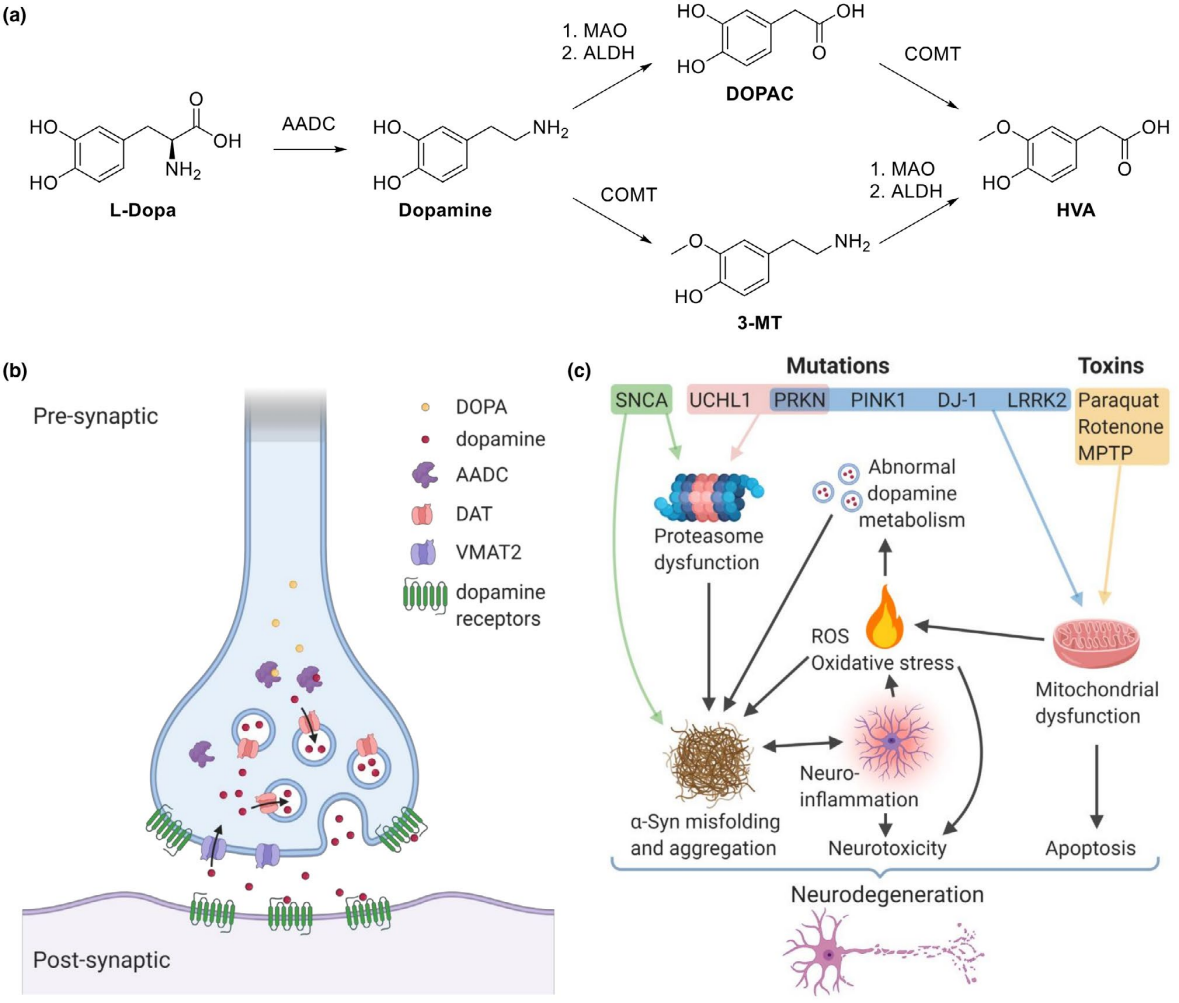
(a) Simplified scheme of dopamine biosynthesis and degradation. (b) Outline of a dopaminergic synapse. (c) Simplified overview of the known mechanisms leading to neurodegeneration in PD. AADC, aromatic‐L‐amino acid decarboxylase; ALDH, aldehyde dehydrogenase; COMT, catechol‐O‐methyl transferase; DAT, dopamine transporter; DOPAC, 3,4‐dihydroxyphenylacetic acid; HVA, homovanillic acid; L‐DOPA, L‐3,4‐dihydroxyphenylalanine; MAO, monoamine oxidase; VMAT2, vesicular monoamine transporter type 2; 3‐MT, 3‐methoxytyramine; SNCA, α‐synuclein gene; UCHL1, ubiquitin carboxyl‐terminal hydroxylase‐1 gene; PRKN, parkin E3 ubiquitin ligase gene; PINK1, phosphatase and tensin homolog‐induced putative putative kinase 1 gene; LRRK2, leucine‐rich repeat kinase 2 gene; ROS, reactive oxygen species; MPTP, 1‐methyl‐4‐phenyl‐1,2,3,6‐tetrahydropyridine. Figure created with BioRender.com

Dopamine is biosynthesized through decarboxylation of L‐3,4‐dihydroxyphenylalanine (L‐DOPA) by aromatic L‐amino acid decarboxylase (AADC, Figure 1a) (Meiser et al., 2013). Dopamine is stored in synaptic vesicles and released into synaptic cleft for signal transduction. Dopamine transporter (DAT) pumps released dopamine back into the presynaptic neuron, and vesicular monoamine transporter (VMAT2) again loads dopamine into the synaptic vesicles until it has to be released again (Figure 1b).

Dopamine that failed to be transported by DAT into the presynaptic neuron becomes degraded by the glial cells: catechol‐*O*‐methyl transferase (COMT) converts it into 3‐methoxytyramine (3‐MT) which is consequently oxidized to homovanillic acid (HVA) by monoamine oxidase‐B (MAO‐B). Inside neurons but outside the synaptic vesicles, dopamine is converted by MAO‐A and MAO‐B isoforms to 3,4‐dihydroxyphenylacetaldehyde (DOPAL), which is promptly oxidized by aldehyde dehydrogenase (ALDH) to 3,4‐dihydroxyphenylacetic acid (DOPAC). DOPAC is then methylated by COMT to form HVA (Meiser et al., 2013).

The metabolism of dopamine by MAO‐B can result in various cytotoxic radical species such as superoxide anions, dopamine‐quinone species and hydroxyl radicals. These radical species can contribute to neurodegeneration by for example forming adducts with toxic α‐syn protofibrils (Dias et al., 2013). In turn, α‐syn aggregates inside neurons can interfere with dopamine storage in the synaptic vesicles, leading to dopamine leakage into the cytoplasm or extracellular space and its conversion to toxic metabolites (Dias et al., 2013; Lotharius & Brundin, 2002).

#### 1.2.3 Genetic and environmental risk factors

Most of PD cases (~85%) are sporadic and idiopathic, while a minority (~15%) can be explained by genetic predisposition (Tran et al., 2020). The α‐Syn gene SNCA was the first gene mutation in which was shown to cause familial PD (Polymeropoulos, 1997). Currently, at least 16 loci associated with familial PD have been identified in the human genome (Singleton et al., 2013; Thomas & Beal, 2007; Tran et al., 2020). Genes most commonly associated with PD include:

‐ SNCA (α‐Syn);
‐ PRKN (parkin E3 ubiquitin ligase) (Poorkaj et al., 2004);
‐ UCHL1 (ubiquitin carboxyl‐terminal hydrolase‐1) (Leroy et al., 1998);
‐ LRRK2 (leucine‐rich repeat kinase 2) (Sanders et al., 2014);
‐ DJ‐1 (DJ‐1 mitochondrial protein) (Bonifati, 2003);
‐ PINK1 (phosphatase and tensin homolog‐induced putative kinase 1) (Valente, 2004).

Mutations in the SNCA gene can increase the propensity of α‐Syn to aggregate. At the same time, mutations in the SNCA, UCHL1, and PRKN genes can impair the functioning of the ubiquitin‐proteasome system, which is responsible for the removal of misfolded proteins like aggregated α‐Syn (Dauer & Przedborski, 2003). The PRKN, LRRK2, DJ‐1, and PINK1 genes are associated with mitochondrial deficiency and oxidative stress (Hauser & Hastings, 2013). Oxidative stress leads to the misfolding and aggregation of α‐Syn, while aggregated α‐Syn damages synaptic vesicles, releasing dopamine into the cytoplasm. Released dopamine is converted into toxic metabolites, which promote further oxidative stress and α‐Syn aggregation, thus closing the feedback loop (Dias et al., 2013; Lotharius & Brundin, 2002). Preclinical models of PD also show that α‐Syn aggregates promote neuroinflammation, while neuroinflammation causes α‐Syn aggregation, which forms another potential feedback loop in PD pathogenesis (Dias et al., 2013; Monahan et al., 2008; Tansey & Goldberg, 2010). Eventually, mitochondrial and cellular damage in dopaminergic neurons can lead to apoptosis. It is likely that idiopathic PD develops similarly to genetically predetermined PD, but the defects in the functioning of the relevant proteins are caused by factors other than mutations.

Fibrillation of α‐Syn and/or death of dopaminergic neurons can also be triggered by exposure to environmental toxins, such as pesticides rotenone and paraquat as well as the recreational drug contaminant 1‐methyl‐4‐phenyl‐1,2,3,6‐tetrahydropyridine (MPTP) (Langston et al., 1983; Tanner et al., 2011). The toxic metabolite of MPTP is structurally similar to paraquat, and both compounds are thought to exert their pathological effects by interfering with mitochondrial function and promoting the formation of free radicals and reactive oxygen species (Dauer & Przedborski, 2003).

The influence of genetic and environmental factors on the development of neurodegeneration in PD is illustrated in Figure 1c.

### 1.3 Treatment of Parkinson's disease

The most common pharmacological treatment strategy for the motor symptoms of PD is to increase the amount of dopamine in the striatum using the dopamine precursor L‐3,4‐dihydroxyphenylalanine (levodopa, L‐DOPA) (Rajput, 2001). L‐DOPA is usually combined with a peripherally active AADC inhibitor (e.g., carbidopa or benserazide) or centrally acting MAO‐B (selegine, rasagiline) and COMT inhibitors (entacapone, tolcapone) to minimize off‐target metabolism of L‐DOPA and degradation of dopamine. This reduces side effects and improves efficiency of the treatment. Nevertheless, the efficiency of L‐DOPA treatment decreases as the disease progresses, indicating a minimum threshold level of dopaminergic neurons required for L‐DOPA to work. Alternatively, postsynaptic dopamine D_2/3_ receptors can be activated directly by agonist drugs such as pramipexole and ropinirole (Ferreira et al., 2012; Zhang & Tan, 2016).

Non‐dopaminergic receptor systems targeted by anti‐parkinsonic treatments include cholinergic system (muscarinic cholinergic antagonist trihexyphenidyl, cholinesterase inhibitor rivastigmine (Pagano et al., 2015), noradrenergic system (norepinephrine transporter ligand amantadine (Sommerauer et al., 2012), and adenosine receptors (A_2A_ antagonist istradefylline (Chen & Cunha, 2020). These treatments are also often combined with L‐DOPA or dopamine agonists to alleviate side effects or address non‐motor symptoms of PD such as depression (Huber et al., 1999; Parkes et al., 1970).

Besides pharmacological treatment, surgical procedures such as deep brain stimulation (DBS) can be an option for certain PD patients. DBS proved highly effective in the improvement of motor functions in advanced PD (Benabid et al., 1994; Nutt et al., 2001). It is speculated that DBS treatment induces neuromodulation, synaptic plasticity, and neuroprotection (Goetz et al., 2005; Herrington et al., 2016). A non‐invasive alternative to DBS recently approved in the United States is magnetic resonance‐guided focused ultrasound ablation (Moosa et al., 2019). However, neither of the two methods can reverse neurodegeneration.

The grafting of neuronal and/or stem cells into the brain to compensate for the death of native dopaminergic neurons is another direction of PD therapy (Barker et al., 2015; Parmar et al., 2020). Original approaches using fetal material were found to be not superior to DBS, but improvements in stem cell technology promise better and more consistent results in the future (Parmar et al., 2020).

Pharmacological treatments targeting α‐Syn aggregation may provide a breakthrough in PD therapy, because α‐Syn aggregation is the key process that lets neurodegeneration spread to new cells. Approaches currently tested in clinical trials use both antibodies and small molecules to reduce the amount of toxic α‐Syn aggregates (Oliveri, 2019; Zella et al., 2019).

Summing up, the currently available treatments for PD focus on the reduction of the symptoms caused by dopaminergic denervation (Armstrong & Okun, 2020). There is an unmet need for therapies addressing the pathophysiology of PD on a more fundamental level. Such therapies should provide more robust and long‐lasting improvements in the patients’ quality of life.

Existing evidence strongly suggests that PD is a heterogeneous disorder, and the term “Parkinson's disease” is at least to some extent an umbrella term covering a family of disorders having different etiologies. Heterogeneity is one of the reasons why the exact mechanism that triggers PD has yet to be elucidated, and a cure has yet to be developed. Imaging of molecular targets involved in PD pathophysiology in living patients should help explain the observed heterogeneity and find the paths toward early diagnosis and new efficient treatments. These targets and imaging methods are discussed in the following sections.

## 2 THE MOLECULAR IMAGING OF PARKINSON'S DISEASE

Molecular imaging is the visualization, characterization, and measurement of biological processes at the molecular and cellular levels (Ametamey et al., 2008; Mankoff, 2007). Therefore, molecular imaging is functional imaging, meaning that it provides functional information about biological processes in vivo. Anatomical imaging, providing information about changes in organ size or tissue structure, is also used to study PD (Saeed et al., 2020; Weingarten et al., 2015), but is outside of the scope of this review.

### 2.1 Principles of molecular imaging methods used to diagnose and study PD

#### 2.1.1 PET

Positron emission tomography (PET) is a nuclear imaging technique which relies on the decay characteristics of positron‐emitting radionuclides such as fluorine‐18 (^18^F, *t*_1/2_ = 109 min), carbon‐11 (^11^C, *t*_1/2_ = 20 min) or oxygen‐15 (^15^O, *t*_1/2_ = 2 min) (Cherry & Dahlbom, 2006). A compound labeled with a positron‐emitting radionuclide, a so‐called tracer, is injected into the test subject and allowed to distribute across the body (Kristensen & Herth, 2017). Upon decay, the radionuclide ejects a positron, which is quickly stopped by the surrounding tissue, combines with an electron, and the two annihilate, producing a pair of gamma photons of 511 keV each. These gamma photons are emitted at an approximately 180 degree angle and are detected at the same time, co‐incidentally, by detectors arranged in rings around the test subject (Peter, 2009). The spatial distribution of the tracer as a function of time can then be inferred from the lists of detected co‐incident gamma impact events (Ametamey et al., 2008).

#### 2.1.2 SPECT

Single photon emission computed tomography (SPECT) is the second major nuclear imaging technique. Like PET, it requires a tracer to be injected into the test subject. However, SPECT uses radionuclides emitting single gamma photons such as technetium‐99m (^99m^Tc, *t*_1/2_ = 6.01 h), indium‐111 (^111^In, *t*_1/2_ = 2.80 d), iodine‐123 (^123^I, *t*_1/2_ = 13.22 h). A series of 2D projection images of tracer distribution in the body are acquired by one of more gamma cameras from multiple angles. These projection images are then assembled to produce a 3D image (Peter, 2009).

SPECT tracers can also be used for scintigraphy, an older imaging technique where only a single projection image of tracer distribution inside the patient's body is recorded (Prince & Links, 2014).

#### 2.1.3 MRI and fMRI

Magnetic resonance imaging (MRI) is an imaging technique that uses the behavior of atomic nuclei with a non‐zero spin in a strong magnetic field to generate images. Hydrogen nuclei (protons) in water molecules are the most abundant such nuclei in the human body. The test subject is placed in a strong magnetic field and exposed to brief electromagnetic energy pulses, which distort the protons’ spin orientations. After the energy pulse is stopped, the spin orientations “relax,” that is, return to equilibrium. The relaxation rates depend on the chemical environment of the nuclei, so recorded MR signal can be used to produce anatomical images of the tissues of interest (Peter, 2009).

MRI can also be used for functional imaging; in which case it is called “functional MRI” (fMRI). Conventional fMRI imaging relies on the detection of deoxygenated hemoglobin in the blood, or blood oxygenation level dependent (BOLD) contrast (Glover, 2011). However, fMRI can also be based on the following of blood water molecules magnetized by arterial spin labeling (ASL) (Glover, 2011) or on the injection of contrast agents. Contrast agents are molecules or nanostructures with specific magnetic properties that make their presence in the tissue detectable by MRI (Jasanoff, 2007).

Synchronization or temporal correlation of fMRI signal in different brain areas is evidence of a functional connection between them. Thus, fMRI can be applied to studies of functional connectivity of the brain and its changes in disease (Weingarten et al., 2015).

#### 2.1.4 Comparison of molecular imaging methods

Four important parameters in molecular imaging are sensitivity, accuracy (quantitativity), temporal and spatial resolution. SPECT scanners used in preclinical research tend to have a better spatial resolution compared to preclinical PET, but clinical PET scanners provide a higher spatial resolution than clinical SPECT scanners (Khalil et al., 2011). PET has higher sensitivity and temporal resolution compared to SPECT and provides essentially quantitative images (Rahmim & Zaidi, 2008). Moreover, hybrid PET/CT or PET/MRI systems providing an anatomical frame of reference to functional imaging data are more widely available than SPECT/CT and SPECT/MRI systems (Davis et al., 2020). Therefore, PET is often the preferred imaging modality for clinical research. Novel nuclear imaging agents for targets that previously could not be imaged are also overwhelmingly developed for the PET modality. Still, PET has its downsides. Both scanners and tracers are relatively expensive, and most PET tracers are labeled with short‐lived isotopes, so that PET imaging centers must be in the vicinity of cyclotrons producing those isotopes. SPECT, an older technique, is less expensive and relies on longer lived isotopes, which can be transported over very long distances. SPECT is still the method of choice in imaging centers, which do not possess or cannot maintain a PET scanner (Beer et al., 2011).

fMRI has a higher spatial and temporal resolution than PET and SPECT, which is convenient in measuring dynamic changes in neural network activity (Kameyama et al., 2016; Peter, 2009). Moreover, fMRI uses no ionizing radiation, and is completely non‐invasive when no contrast agents are used. In the latter case, there is in principle no limitation to the number of scans that can be performed each day. However, compared to PET and SPECT, MRI has a poorer signal to noise ratio, and the quantification of the imaging data is much more complicated and unreliable (Pysz et al., 2010).

### 2.2 Goals of molecular imaging in PD

Molecular imaging in PD patients typically pursues one or more of the four following goals:

1. Supporting the diagnosis of PD in the clinic and during patient recruitment for clinical trials by confirming the presence of expected pathological changes and/or ruling out alternative causes of observed symptoms,
2. Assessing the disease progression,
3. Studying differences between patients with PD and healthy controls,
4. Evaluating the effect of treatment.

Goal (3) does not include clinical diagnosis of PD, but rather fundamental research into pathological changes in PD. Goal (4) includes the use of molecular imaging outcomes as biomarkers to monitor the efficiency of PD treatment and the assessment dose‐occupancy relationships for drugs investigated as PD treatments.

### 2.3 Targets used in PD imaging

An overview of imaging targets investigated in PD, and the agents used to image them is presented in Table 1.

**TABLE 1:** Imaging targets used in PD diagnostics and research

Entry	Target	Modality	Examples of imaging agents	Application in PD diagnostics and research
1	Dopamine synthesis (AADC)	PET	[^18^F]F‐DOPA [^18^F]FMT	Diagnostic and research use: detecting loss of nigrostriatal dopaminergic nerve endings [^18^F]F‐DOPA approved in the EU and the US in 2019 for diagnosing PD and distinguishing ET from parkinsonian syndromes
2	Dopamine transporter	PET, SPECT	PET: [^18^F]FE‐PE2I SPECT: [^123^I]FP‐β‐CIT	Diagnostic and research use: detecting loss of nigrostriatal dopaminergic nerve endings [^123^I]FP‐β‐CIT (DatScan ®) approved in the EU and the US for diagnosing PD and distinguishing ET from parkinsonian syndromes
3	Vesicular monoamine transporter	PET	[^11^C/^18^F]DTBZ	Research use only: detecting loss of nigrostriatal dopaminergic nerve endings
4	Dopamine D_2/3_ receptors	PET, SPECT	PET: [^11^C]raclopride (+)‐[^11^C]PHNO [^18^F]fallypride SPECT: [^123^I]IBZM	Research use only: detecting loss of striatal neurons in MSA and PSP, measuring dopamine release, assessing receptor occupancy by D_2/3_‐targeting anti‐PD medications
5	Serotonin transporter (SERT)	PET, SPECT	PET: [^11^C]DASB [^18^F]F‐DOPA (off‐target binding) SPECT: [^123^I]FP‐CIT (off‐target binding)	Research use only: detecting loss of serotonergic nerve endings in the raphe nuclei
6	Serotonin 5‐HT_1_ receptors	PET	5‐HT_1A_: [^11^C]WAY100635 [^18^F]MPPF 5‐HT_1B_: [^11^C]AZ10419369	Research use only: loss of 5‐HT_1_R availability found in the cortex
7	Serotonin 5‐HT_2A_ receptors	PET	[^18^F]setoperone [^11^C]Cimbi−36	Research use only: changes in 5‐HT_2A_R availability found in PD patients with hallucinations
8	Vesicular acetylcholine transporter (VAChT)	PET, SPECT	PET: [^18^F]FEOBV SPECT: [^123^I]IBVM	Research use only: loss of VAChT found in the cortex
9	Acetylcholine esterase (AChE)	PET	[^11^C]MP4A [^11^C]PMP 5‐[^11^C]methoxydonepezil	Research use only: loss of AChE found in the cortex and in the peripheral nervous system
10	Nicotinic acetylcholine receptors (α4β2)	PET, SPECT	PET: 2‐[^18^F]fluoro‐A‐85380 SPECT: [^123^I]5‐IA‐85380	Research use only: loss of α4β2 found across the brain
11	Muscarinic acetylcholine receptors	PET, SPECT	PET: [^11^C]NMPB SPECT: [^123^I]QNB	Research use only: increase in muscarinic receptor availability found in the cortex
12	Norepinephrine transporter (NET)	PET	[^11^C]MeNER	Research use only: loss of NET found in the midbrain and thalamus
13	Norepinephrine synthesis in the heart (NET, VMAT2)	PET, SPECT	PET: [^11^C]HED SPECT: [^123^I]MIBG	Diagnostic and research use: detecting loss of cardiac noradrenergic innervation [^123^I]MIBG approved in Japan for diagnosing PD and distinguishing ET from parkinsonian syndromes
14	Synaptic terminals (SV2A)	PET	[^11^C]UCB‐J	Research use only: loss of synaptic terminals found in SN, cortical synaptic density decreased in PD patients with dementia
15	Glucose metabolism	PET	[^18^F]FDG	Research use only: detecting PD‐related patterns of metabolism/blood flow/functional connectivity [^18^F]FDG PET and fMRI show promise in diagnosing PD and distinguishing between PD and atypical PS on an individual level, but are not used for this purpose on a routine basis
16	Cerebral blood flow	PET, SPECT, fMRI	PET: [^15^O]H_2_O SPECT: [^99m^Tc]Tc‐ECD fMRI: none	
17	Neural connectivity	fMRI	None	
18	Microglia (TSPO)	PET	(*R*)‐[^11^C]PK11195 [^18^F]FEPPA	Research use only: studying microglia activation in PD Elevation of TSPO expression across the brain found in initial studies, but not confirmed in follow‐up studies
19	Adenosine A_2A_ receptors	PET	[^11^C]SCH442416 [^11^C]TMSX [^11^C]preladenant	Research use only: measuring occupancy of A_2A_ targeting drugs Increase in striatal A_2A_ availability found in PD with dyskinesias
20	Cannabinoid CB1 receptors	PET	[^18^F]MK‐9470	Research use only: increase in CB1 availability found in the striatum
21	N‐methyl‐d‐aspartate receptor (NMDA)	PET	[^11^C]CNS5161	Research use only: increase in striatal and cortical NMDAR availability found in PD patients with LID
22	Phosphodiesterase enzymes (PDE1‐11)	PED	PDE4: [^11^C]rolipram PDE10A: [^11^C]IMA107	Research use only: loss of PDE found in striatal and cortical regions
23	Neuromelanin	PET MRI	PET: [^18^F]AV1451 (off‐target binding) MRI: None	Research use only: loss of neuromelanin found in SN and locus coeruleus (LC)
24	Beta‐amyloid	PET	[^11^C]PIB [^18^F]florbetaben [^18^F]florbetapir [^18^F]flutametamol	Diagnostic and research use: imaging beta‐amyloid accumulation The three ^18^F‐tracers approved for use in AD diagnosis in the US and Europe DLB patients tend to have higher beta‐amyloid load than PD patients
25	Tau	PET	[^18^F]AV1451 [^18^F]FDDNP	Diagnostic and research use: imaging tau fibril load [^18^F]AV1451 approved in the US for AD diagnosis PSP patients tend to have higher tau load than PD patients

#### 2.3.1 Dopaminergic system

Deterioration of dopaminergic signaling is well documented in PD; therefore various elements of dopaminergic system have been used as target for imaging.

##### Dopamine synthesis and metabolism

The activity of enzymes involved in dopamine synthesis and metabolism can be imaged by PET with the aid of 6‐[^18^F]fluoro‐DOPA ([^18^F]F‐DOPA; Table 1, row 1), a ^18^F‐fluorinated analog of L‐DOPA (Garnett et al., 1983). Uptake of [^18^F]F‐DOPA in the striatum is a composite function of the density of catecholaminergic nerve terminals, activity of AADC and VMAT2 involved in dopamine synthesis (Toch et al., 2019). Washout of [^18^F]F‐DOPA reflects the activity of COMT and MAO which are involved in dopamine metabolism (Figure 1a). Imaging is normally performed during the first 90–120 min post‐injection, when the activity monotonously accumulates in a healthy striatum (Kuriakose & Stoessl, 2010).

**FIGURE 2:**
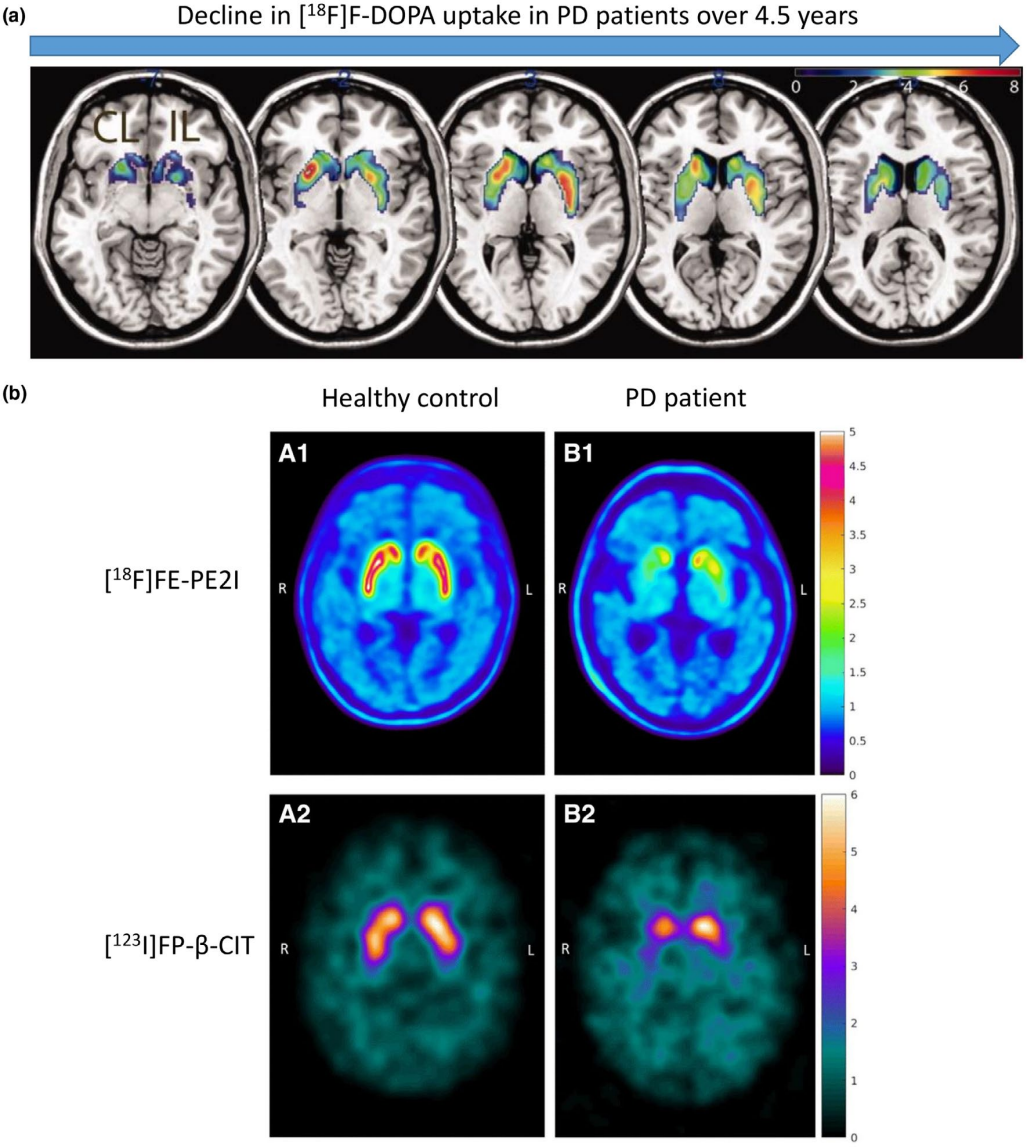
Examples of dopaminergic system imaging in PD research and diagnostics. (a) Decline of striatal [^18^F]F‐DOPA uptake in PD patients after 4.5 years of progressing disease. CL, contralateral side, IL, ipsilateral side. The colored scale bar indicates voxel‐level t‐statistic. Reproduced and adapted from (Gallagher, Oakes, et al., 2011) with permission. (b) Comparison of [^18^F]FE‐PE2I‐PET and [^123^I]FP‐β‐CIT‐SPECT images in the same individuals. Reproduced and adapted from (Jakobson Mo et al., 2018) under the terms of the Creative Commons Attribution 4.0 International License

In PD, [^18^F]F‐DOPA uptake in striatal regions decreases compared to healthy state and atypical PS (Figure 2a). The decrease is usually asymmetrical: signal loss is greater on one side of the brain than on the other (Kuriakose & Stoessl, 2010). Moreover, posterior parts of the putamen are usually more affected than anterior parts (Ibrahim et al., 2016). The decrease correlates with the severity of certain motor symptoms, especially rigidity and bradykinesia (Niethammer et al., 2012). However, non‐motor symptoms, such as cognitive impairment and depression, as well as some non‐motor symptoms, such as tremors, do not correlate with [^18^F]F‐DOPA uptake, which suggests that they are caused by other factors beyond dopaminergic terminal degradation (Broussolle et al., 1999; Otsuka et al., 1996). Imaging of PD patients after dopaminergic cell implantation demonstrated an increase of striatal [^18^F]F‐DOPA uptake, confirming partial normalization of dopaminergic function (Ma, Tang, et al., 2010; Politis et al., 2012). From [^18^F]F‐DOPA uptake measurements in PD patients taken at two timepoints 18 months apart, it was estimated that the average preclinical (asymptomatic) period of PD is likely no longer than 7 years (Morrish et al., 1998). [^18^F]F‐DOPA was officially approved in the EU in 2006 and in the United States in 2019 for diagnosing PD and distinguishing essential tremor from parkinsonian syndromes, that is, PD and atypical PS (Chevalme et al., 2007; NDA 200655).

6‐[^18^F]fluoro‐m‐tyrosine ([^18^F]FMT) is a structural analog of [^18^F]F‐DOPA which has a higher affinity for AADC and is not recognized by COMT, while its metabolites also have lower affinity toward DAT and VMAT2 compared to corresponding metabolites of [^18^F]F‐DOPA (Li et al., 2014). Therefore, compared to [^18^F]F‐DOPA, [^18^F]FMT has fewer radiometabolites that confound the interpretation of the results, and its uptake in the striatum should more closely reflect AADC activity. In a within‐subject comparison study of 12 PD patients, [^18^F]FMT signal indeed correlated with clinical symptoms better than did [^18^F]F‐DOPA signal (Gallagher, Christian, et al., 2011). However, neither [^18^F]F‐DOPA nor [^18^F]FMT uptake in extrastriatal regions could be correlated to cognitive and emotional symptoms of PD in another study on 15 patients (Li et al., 2014).

##### Dopamine transporter activity

The dopamine transporter (DAT) is a protein expressed on the presynaptic membrane. DAT is involved in the reuptake of dopamine from the synaptic cleft and regulation of dopamine storage in synaptic vesicles (Table 1, row 2).

There are both PET and SPECT tracers for DAT imaging and almost all of them are cocaine/tropane derivatives. Indeed, [^11^C]cocaine, a PET tracer, was one of the first radio ligands that could accurately image DAT in vivo (Fowler et al., 1989). However, it was the SPECT tracer [^123^I]β‐CIT that allowed DAT imaging to become a widely used supporting tool for PD diagnostics (Neumeyer et al., 1991). The drawbacks of [^123^I]β‐CIT were the lack of selectivity for DAT against serotonin transporters (SERT) and very slow pharmacokinetics that required 24 h intervals between tracer injection and imaging (Ziebell et al., 2010). Therefore, it was superseded by [^123^I]FP‐β‐CIT, which has an improved DAT‐over‐SERT selectivity and faster pharmacokinetics, so that image acquisition can be started 3–4 h after injection (Seibyl et al., 1998; Winogrodzka, 2003; Ziebell et al., 2010). SPECT tracers with even greater selectivity toward DAT and even faster pharmacokinetics include [^123^I]PE2I and [^123^I]altropane (Fischman et al., 1998; Kuikka et al., 1998). Another interesting example of a DAT SPECT tracer is [^99m^Tc]Tc‐TRODAT‐1 (Kung et al., 1996). It is currently the only chelator‐based nuclear imaging agent that can cross the intact blood–brain‐barrier (BBB). The main advantage of [^99m^Tc]Tc‐TRODAT‐1 is its low price compared to tracers labeled with cyclotron‐produced isotopes (e.g., ^123^I or ^18^F), because ^99m^Tc can be obtained from ^99^Mo/^99m^Tc generators. This might be the reason why studies with [^99m^Tc]Tc‐TRODAT‐1 are mostly performed in Asia and South America (Ferraz, 2014; Hossein‐Tehrani et al., 2020; Hwang et al., 2004; Sasannezhad et al., 2017; Sood et al., 2021).

PET tracers for DAT imaging include both compounds chemically identical to SPECT ligands, where a different atom in their structure replaced by a positron‐emitting radionuclide, and compounds specifically developed for PET imaging. Tracers belonging to the former category include [^11^C]FP‐β‐CIT, [^18^F]FP‐β‐CIT (Lundkvist et al., 1997) and [^11^C]PE2I (Appel et al., 2015). The latter category includes, for example, two non‐tropane DAT tracers: [^11^C]‐d‐*threo*‐methylphenidate (Ding et al., 1997) and the norepinephrine transporter‐preferring [^11^C]nomifensine (Aquilonius et al., 1987; Tatsumi et al., 1997). An important objective in the development of DAT tracers dedicated for PET is reaching faster pharmacokinetics, because PET isotopes like ^18^F and ^11^C have shorter half‐lives than SPECT isotopes like ^123^I and ^99m^Tc. For example, the striatal uptake of [^11^C]‐d‐*threo*‐methylphenidate and [^18^F]FE‐PE2I in healthy humans peaks within 15 min after injection (Jakobson Mo et al. 2018; Ding et al., 1997). Other DAT PET tracers have slower pharmacokinetics: time from injection until peak striatal uptake is 30–60 min for [^11^C]PE2I (Appel et al., 2015), 40–50 min for [^18^F]PR04.MZ (Juri et al., 2021; Kramer, Juri, et al., 2020), >80 min for [^11^C]RTI‐32 (Guttman et al., 1997), and 3–4 h for [^18^F]CFT (Laakso et al., 1998). Tracers that reach peak brain uptake faster have two advantages over tracers with slower pharmacokinetics. First, the bias in the estimates of target availability (DAT in the current context) is reduced, because the rate of the tracer's dissociation from its target can be estimated more precisely. Second, sufficient amount of imaging data for precise quantification can be obtained from a shorter scan, which means less discomfort for the patient (DeLorenzo et al., 2009; Sasaki et al., 2012).

Like in dopamine synthesis imaging, DAT signal in PD is decreased compared to healthy state, and the decrease correlates with the severity of bradykinesia, but not with tremor severity (Benamer et al., 2000; Li et al., 2018; Seibyl et al., 1995). Intriguingly, a correlation has been found between DAT signal (imaged by SPECT tracer [^123^I]FP‐β‐CIT and PET tracer [^11^C]RTI‐32) and depressive symptoms of PD (Remy et al., 2005; Vriend et al., 2014). A single dose of L‐DOPA results in a short‐term decrease of DAT availability, suggesting DAT occupancy by the newly synthesized dopamine (Mishina et al., 2011). Long‐term L‐DOPA treatment, however, accelerates the loss of DAT binding in progressing PD, although this can be remedied by adjunct therapy with dopamine receptor agonists (Ikeda et al., 2019). Nevertheless, these findings show that the outcomes of DAT imaging can be distorted by anti‐PD therapy.

DAT imaging with [^123^I]FP‐β‐CIT (also called [^123^I]FP‐CIT, ioflupane, or DaTSCAN®) was approved for clinical use by EMA in 2000 and by FDA in 2011 (EMEA/H/C/000266; Park, 2012). The intended application is differentiating PD from ET, but not from atypical PS (Roussakis et al., 2013).

To sum up, the imaging of DAT in PD diagnostics is used in very much the same way as dopamine synthesis imaging (Figure 2b). PET tracer [^18^F]FE‐PE2I may eventually overtake the SPECT tracer [^123^I]FP‐β‐CIT as the most popular nuclear imaging agent for DAT, because it has the same diagnostic performance but has faster pharmacokinetics and benefits from overall advantages of PET over SPECT (Jakobson Mo et al. 2018).

##### Vesicular monoamine transporter type 2

Vesicular monoamine transporter type 2 (VMAT2) is located in the membrane of synaptic vesicles where dopamine is stored (Figure 1b; Table 1, row 3). VMAT2 can be imaged with the PET tracers [^11^C](+)dihydrotetrabenazine ([^11^C]DTBZ) and 9‐[^18^F]fluoropropyl‐(+)‐dihydrotetrabenazine ([^18^F]DTBZ or [^18^F]AV‐133). Presynaptic degeneration in PD results in a decrease of striatal uptake of VMAT2 tracers compared to healthy state (Pérez‐Lohman et al., 2018; Jung Lung et al. 2018). Within‐subject comparison of [^18^F]F‐DOPA, [^11^C]‐d‐*threo*‐methylphenidate and [^11^C]DTBZ showed that, in PD patients, striatal uptake of [^18^F]F‐DOPA was reduced less than the binding of [^11^C]DTBZ, while the binding of [^11^C]‐d‐*threo*‐methylphenidate was reduced more than of [^11^C]DTBZ (Lee et al., 2000). This suggests that VMAT2 availability may be a less biased biomarker of PD progression than presynaptic dopamine synthesis activity or DAT availability. Moreover, preclinical data show that the binding of VMAT2 tracers is less likely to be distorted by anti‐PD medication than the binding of DAT or dopamine synthesis tracers (Kilbourn et al., 1996). It has been estimated that VMAT2 density starts to decline ~17 years before PD symptoms appear (De La Fuente‐Fernández et al., 2011), so VMAT2 imaging may be helpful in studying PD development throughout the presymptomatic phase.

##### Dopamine receptors

Imaging of dopamine receptors provides information about the neurons on the postsynaptic side of dopaminergic nerve terminals, as the majority of those receptors are located on the postsynaptic membranes (Figure 1b; Table 1, row 4). Most dopaminergic receptor tracers bind to either D_1_‐like (D_1_ and D_5_) or D_2_‐like (D_2_, D_3_ and D_4_) receptor subfamilies (Elsinga et al., 2006). D_2_‐like receptors are often referred to as D_2/3_ receptors, because D_4_ subtype is much less abundant than the former two.

Radioligands for D_1_‐like receptor imaging are relatively few and limited to PET tracers such as [^11^C]NNC112 and [^11^C]SCH23390 (Elsinga et al., 2006). On the contrary, radioligands for D_2/3_ receptors are numerous and include both SPECT tracers such as [^123^I]IBZM and PET tracers such as [^11^C]raclopride, [^18^F]desmethoxyfallypride and (+)‐[^11^C]PHNO (Elsinga et al., 2006; Mukherjee et al., 1996). These tracers are suitable for the imaging of D_2/3_‐rich striatal regions. Extrastriatal regions, having much lower D_2/3_ densities, can be imaged with ultra‐high‐affinity PET tracers such as [^11^C]FLB457 and [^18^F]fallypride (Mukherjee et al., 2002). As opposed to D_1_‐like receptor tracers, virtually all D_2/3_ tracers are sensitive to intrasynaptic dopamine concentrations (Laruelle, 2000, 2012), (+)‐[^11^C]PHNO having the highest sensitivity (Shotbolt et al., 2012). Sensitivity to intrasynaptic dopamine is both an advantage and a disadvantage. On the one hand, the measurement of changes in receptor availability before and after triggered dopamine release (e.g., administration of amphetamine) can be used to monitor the recovery of dopaminergic signaling after anti‐PD treatment (Piccini et al., 1999). On the other hand, single measurements of receptor availability are hard to interpret, because they are influenced both by receptor density and dopamine concentration (Thobois et al., 2010).

PET imaging of D_1_‐like receptors in PD failed to detect any differences in receptor availability between PD and healthy state (Cropley et al., 2008; Rinne et al., 1990). D_2/3_ receptor availability is also normal or elevated in PD patients, although it declines as the disease progresses, and the decline is steeper in extrastriatal regions than in the striatum (Kaasinen et al., 2003). In atypical PS, the baseline availability of striatal D_2/3_ receptors is significantly lower than in PD (Antonini et al., 1997; Schreckenberger et al., 2004; Vlaar et al., 2008; La Fougère et al. 2010). Therefore, PET and SPECT imaging of D_2/3_ receptors has been promoted as a method of differential diagnosis between PD and atypical PS. PET imaging of D_2/3_ receptors has also been used to confirm their occupancy by dopamine and D_2/3_ agonists after respective anti‐PD treatments (Deutschländer et al., 2016; Mishina et al., 2011).

Dopamine receptors belonging to the D_3_ subtype are thought to be the main target of anti‐parkinsonic dopaminergic drugs (Joyce & Millan, 2007; Silverdale et al., 2004). Therefore, selective imaging of D_3_ can be advantageous. Binding of the D_3_‐preferring tracer (+)‐[^11^C]PHNO was found to correlate with motor deficits and lowered mood in PD patients (Boileau et al., 2009). (+)‐[^11^C]PHNO is actively used in clinical occupancy studies of D_3_‐preferring antipsychotic drugs (Le Foll et al. 2014; Di Ciano et al. 2019). Development of D_3_‐selective PET tracers is underway (Mach & Luedtke, 2018).

Summing up, although multiple tracers for PET and SPECT imaging of dopamine receptors in humans are available, their clinical value has so far been limited, and imaging experiments with dopamine receptor tracers in PD patients are mostly performed for research purposes.

#### 2.3.2 Serotonergic system

Serotonin is a monoamine neurotransmitter which, like dopamine, is produced from an amino acid (tryptophan), stored in synaptic vesicles, released into the synaptic cleft, and re‐captured by the respective transporter protein (Visser et al., 2011). The serotonergic neurons project from the raphe nuclei in the brain stem to most regions in the brain and are involved in regulation of emotions, sleep, motor activity and cognition. Depression, cognitive impairment, and sleep disorder are among the non‐motor symptoms of PD (Kohl & Winkler, 2020), which is why the involvement of serotonergic system in PD is being investigated by molecular imaging (for reviews, see Pagano & Politis, 2018; Politis & Niccolini, 2015).

##### Serotonin transporter (SERT)

SERT can be imaged with PET tracers [^11^C]McN5652 and [^11^C]DASB (Table 1, row 5). [^11^C]DASB is considerably more popular than [^11^C]McN5652 due to its much greater SERT‐over‐DAT selectivity and more favorable pharmacokinetics (Frankle et al., 2004).

Tracers for dopamine transporter imaging such as [^123^I]FP‐β‐CIT have some off‐target affinity for SERT. Therefore, they can also be used for SERT imaging in SERT‐rich regions of the brain, primarily raphe nuclei (Pasquini et al., 2020; Qamhawi et al., 2015). It was even shown that [^18^F]F‐DOPA uptake in the raphe nuclei reflected SERT availability (Pavese et al., 2012).

**FIGURE 3:**
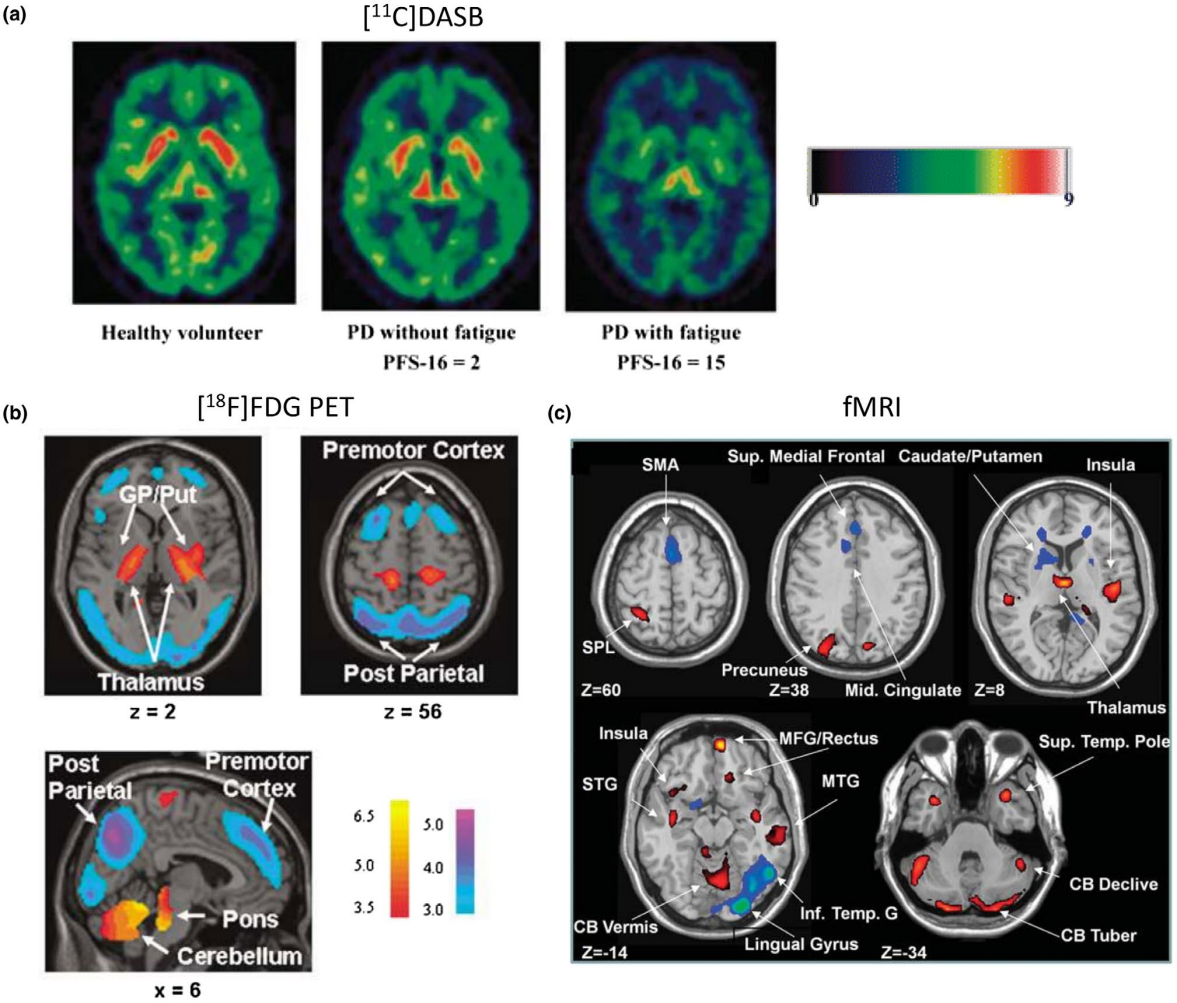
Examples of non‐dopaminergic imaging in PD research and diagnosis. (a) Association of SERT availability with fatigue in PD. Brain uptake of the SERT tracer [^11^C]DASB shown in a healthy control (left), PD patient without (middle) and with fatigue (right). Color bar shows [^11^C]DASB binding potential. Reproduced and adapted from (Pavese et al., 2010) with permission. (b) PD‐related pattern (PDRP) in brain glucose metabolism identified by network analysis of [^18^F]FDG scans in PD patients and healthy controls. Color coding indicates areas with increased (red to yellow) and decreased (blue to purple) metabolism. Reproduced from (Ma et al., 2007) with permission. (c) PD‐related pattern in neural activity identified network analysis of resting state fMRI scans in PD patients and healthy controls. Color coding indicates increased (red) and decreased (blue) neural activity. Reproduced from (Wu et al., 2015) with permission

A systematic review of SERT imaging studies in PD showed that PD patients have consistently lower SERT availability in raphe nuclei, striatal, and thalamic regions compared to healthy controls (Pagano et al., 2017). Association of SERT availability with both motor (tremors and dyskinesia) and non‐motor symptoms (depression, fatigue) was found (Figure 3a).

[^11^C]DASB imaging of patients with dopamine grafts was used to show that their serotonergic neurons were still degenerated, even though the grafts had restored the dopaminergic function (Politis et al., 2012).

##### Serotonin receptors

Serotonin receptors (5‐HTR) are a diverse group of receptors separated into 7 subfamilies (5‐HT_1‐7_R), some of which are split into further subtypes (5‐HT_1A_, 5‐HT_1B_ etc.) (Paterson et al., 2010). Imaging studies in PD patients have looked at subtypes 5‐HT_1A_, 5‐HT_1B_, and 5‐HT_2A_ (Table 1, rows 6 and 7).

Receptors of the 5‐HT_1A_ subtype can be imaged with PET tracers [^11^C]WAY100635 and [^18^F]MPPF. The availability of these receptors in raphe and cortical areas was found to be decreased in PD patients, and the degree of decrease correlated with the severity of tremor and with depression (Doder M. et al. 2003; Ballanger et al., 2012).

Receptors of the 5‐HT_1B_ subtype can be imaged with the PET tracer [^11^C]AZ10419369. Two studies with this tracer found 5‐HT_1B_ availability in the brain to be decreased in PD patients compared to healthy controls. Receptor availability also correlated with creative ability, suggesting a role of 5‐HT_1B_ receptors in cognitive symptoms of PD (Varrone et al., 2014, 2015).

PET tracers for the serotonin 5‐HT_2A_ receptors are numerous and include [^11^C]MDL100907, (*R*)‐[^18^F]MH.MZ, [^18^F]altanserin, [^18^F]setoperone, and [^11^C]Cimbi‐36 (Blin et al., 1990; Ettrup et al., 2016; Herth & Knudsen, 2015; Kramer, Dyssegaard, et al., 2020; L’Estrade et al., 2018). Hallucinogenic drugs are known to exert their effect by activating 5‐HT_2A_ receptors. A study with [^18^F]setoperone was performed in PD patients with and without visual hallucinations (Ballanger et al., 2010). In agreement with the original rationale, [^18^F]setoperone binding in cortical areas responsible for vision was higher in PD patients with visual hallucinations.

Despite interesting findings, serotonin receptor imaging studies performed to date in PD patients are performed on small subject groups, so their reproducibility and the relevance of detected effects for PD pathogenesis require further confirmation.

#### 2.3.3 Cholinergic system

Acetylcholine, the first neurotransmitter identified as such, regulates brain activities that require selective attention (Perry et al., 1999). Dementia and sleep disturbances observed in PD patients are thought to be associated with the loss of cholinergic neurotransmission (Müller & Bohnen, 2013). Detailed reviews on the imaging of acetylcholinergic system in PD have been published recently (Bohnen et al., 2018; Shinotoh et al., 2021). A short summary follows below.

##### Vesicular acetylcholine transporter (VAChT)

VAChT transports acetylcholine into synaptic vesicles at acetylcholinergic terminals (Bohnen et al., 2018; Table 1, row 8). This transporter can be imaged with the SPECT tracer [^123^I]iodobenzovesamicol ([^123^I]IBVM) and the PET tracer [^18^F]fluoroethoxy benzovesamicol ([^18^F]FEOBV). Both tracers demonstrated significantly lower availability of VAChT in cortical regions of PD patients compared to healthy controls (Kuhl et al., 1996; Zee et al., 2019). Moreover, in a SPECT study with [^123^I]IBVM, PD patients with dementia had more severe and extensive decrease in tracer binding than PD patients without dementia (Kuhl et al., 1996).

##### Acetylcholine esterase (AChE)

AChE is an enzyme that hydrolyzes acetylcholine and regulates its concentration at the cholinergic synapses (Shinotoh et al., 2021; Table 1, row 9). Two PET tracers primarily used for AChE imaging in humans are [^11^C]methyl‐4‐piperidyl acetate ([^11^C]MP4A) and [^11^C]methyl‐4‐piperidyl propionate ([^11^C]PMP). Both tracers are AChE substrates and form radiometabolites which are irreversibly trapped in the brain. [^11^C]MP4A has a somewhat higher specificity for AChE over butyrylcholine esterase, a less selective esterase enzyme that also hydrolyzes acetylcholine (Darvesh et al., 2003; Irie et al., 1996). N‐[^18^F]Fluoroethylpiperidin‐4‐ylmethyl acetate ([^18^F]FEP‐4MA), a ^18^F‐labeled analog of [^11^C]MP4A may also be suitable for AChE imaging, although the radiometabolite formed after hydrolysis by AChE is slowly eliminated from the brain (Ohya et al., 2011). Another PET tracer is the reversible AChE inhibitor 5‐[^11^C]methoxydonepezil, which, however, also binds to sigma receptors in the brain (Hiraoka et al., 2012; Ishikawa et al., 2009).

AChE imaging studies in PD patients mostly showed a decrease in cortical AChE activity compared to healthy controls, and the magnitude of the decrease was greater in patients with dementia (see e.g., Bohnen et al., 2007; Hilker et al., 2005). In patients with MSA and PSP, reductions in thalamic AChE activity tended to be more prominent than in PD patients (Gilman et al., 2010; Shinotoh et al., 1999).

LRRK2 mutation carriers, both healthy and with PD, were found to have higher cortical AChE activity than, respectively, healthy controls and patients with idiopathic PD (Liu et al., 2018).

AChE activity was also found to be decreased in the small intestine and pancreas of PD patients (Gjerløff et al., 2015). This finding corroborates the hypothesis that in some cases PD pathogenesis may start from α‐Syn fibrillation in the gut.

##### Cholinergic receptors

Cholinergic receptors are divided into nicotinic receptors, which are ion channels, and muscarinic receptors, which belong to the GPCR superfamily (Bohnen et al., 2018). These receptor groups are further divided into subtypes (Table 1, rows 10 and 11).

Of the nicotinic receptors, the role of the α4β2 subtype has been investigated in PD. These receptors can be imaged by PET with 2‐[^18^F]fluoro‐3‐[2(*S*)‐2‐azetidinylmethoxy]pyridine (2‐[^18^F]fluoro‐A‐85380) and by SPECT with [^123^I]5‐iodo‐3‐[2(*S*)‐2‐azetidinylmethoxy]pyridine ([^123^I]5‐IA‐85380). Scans of PD patients with both tracers showed widespread losses of α4β2 receptor availability compared to healthy controls in the cortex, thalamus, and striatum (e.g., Fujita et al., 2006; Meyer et al., 2009).

Five subtypes of muscarinic receptors (M_1_–M_5_) exist (Eglen, 2012), but PET and SPECT tracers for muscarinic receptors generally bind to several subtypes at once. SPECT imaging of PD patients with an M_1_/M_4_ tracer [^123^I]QNB showed increased binding in the occipital lobe of the cortex (Colloby et al., 2006). PET imaging with the subtype‐unselective tracer [^11^C]NMPB showed increased receptor availability in the frontal cortex of PD patients compared to healthy controls (Asahina et al., 1995). Therefore, muscarinic receptors appear to be up‐regulated rather than degraded in PD, which may be a compensatory mechanism.

#### 2.3.4 Noradrenergic system

Norepinephrine (noradrenaline) is a catecholamine neurotransmitter similar to dopamine. In the brain, it is primarily synthesized in the locus coeruleus (LC)—a nucleus in the brainstem. The noradrenergic system modulates a wide range of functions, including attention, mood, movement, memory, and cognition. Norepinephrine is also the main neurotransmitter of the sympathetic nervous system, a part of the peripheral nervous system (Nahimi et al., 2018).

##### Norepinephrine transporter (NET)

Deficits in noradrenergic transmission are thought to underlie the non‐motor symptoms in PD patients (Nahimi, Sommerauer, et al., 2018; Table 1, row 12). Recently, the availability of NET in PD patients was studied by PET using the selective NET inhibitor (*S*,*S*)‐[^11^C]‐2‐(α‐(2‐methoxyphenoxy)benzyl)morpholine ([^11^C]MeNER) (Nahimi, Sommerauer, et al., 2018; Sommerauer et al., 2018). In both studies, the binding of [^11^C]MeNER in NET‐rich regions (midbrain and thalamus) was lower in PD patients that in healthy controls. Moreover, in PD patients with a sleep behavior disorder, [^11^C]MeNER binding inversely correlated with the disorder severity (Sommerauer et al., 2018).

##### Noradrenergic innervation of the heart

Neuropathological alterations in PD are not limited to the central nervous system. As discussed in Section 1.2.1, α‐Syn fibrils are found in the gut of PD patients and may even first arise there and subsequently spread to the brain and across the body through nerve connections (Horsager et al., 2020; Table 1, row 13). It is no surprise then that sympathetic nerve endings in the heart are also degenerated in PD (Orimo et al., 2012).

Cardiac innervation can be imaged by PET and SPECT using radiolabeled catecholamines and their mimetics (Langer & Halldin, 2002). These compounds are recognized by NET and VMAT2 and thus accumulate in healthy noradrenergic terminals. The SPECT tracer [^123^I]meta‐iodobenzylguanidine ([^123^I]MIBG) is used in clinical practice for differential diagnostics of PD (Orimo et al., 2012). Heart uptake of [^123^I]MIBG is decreased in PD but stays normal in essential tremor and atypical PS. The use of [^123^I]MIBG for this purpose was approved in Japan in 2012, while the US FDA has so far only approved [^123^I]MIBG for the diagnostics of heart failure (Travin et al., 2019).

PET tracers for cardiac innervation imaging include [^11^C]meta‐hydroxyephedrine ([^11^C]HED) and 6‐[^18^F]fluorodopamine ([^18^F]FDA). Preclinical data suggest that [^11^C]HED uptake in the heart should more specifically reflect norepinephrine transporter function than [^123^I]MIBG uptake (Berding et al., 2003). Both [^11^C]HED and [^18^F]FDA were evaluated in PD patients and showed decreased heart uptake compared to healthy controls and MSA patients (only [^11^C]HED) (Berding et al., 2003; Goldstein, 2000; Wong et al., 2017). Nevertheless, their value in differential PD diagnosis remains to be investigated in more detail.

#### 2.3.5 Synaptic terminals

Damage to synaptic vesicles in synaptic terminals is known to play a role PD pathogenesis, and disruption in synaptic vesicle trafficking is likely to happen at an early stage in neurodegeneration (Esposito et al., 2012; Table 1, row 14). Synaptic vesicle protein 2A (SV2A) is a transmembrane protein which is ubiquitously expressed in all pre‐synaptic terminals at relatively constant levels (Heurling et al., 2019). Therefore, SV2A is a good biomarker for synaptic terminal density.

SV2A can be imaged with the PET tracer [^11^C]UCB‐J (Finnema et al., 2016). Two [^11^C]UCB‐J imaging studies showed a decrease in SV2A availability in SN in patients with early PD compared to healthy controls (Delva et al., 2020; Matuskey et al., 2020). A third study in patients with PD‐related dementia revealed decreased SV2A availability not only in SN, but also in cortical regions, where the observed decrease correlated with the patients’ levels of cognitive function (Andersen et al., 2021). These findings suggest that the loss of SV2A does indeed play a role in PD and might be a contributing factor to cognitive impairment observed in advanced PD. However, more studies are necessary to validate SV2A as a biomarker for PD.

#### 2.3.6 Neural connectivity, cerebral blood flow, and metabolism

Neurodegenerative diseases like PD cause system‐level changes in brain functioning, leading to altered patterns of blood flow, oxygen and energy consumption, and interregional connectivity in comparison to healthy brain (Table 1 rows 15–17). Brain energy consumption can be imaged with the PET tracer [^18^F]fluorodeoxyglucose ([^18^F]FDG) (Meles et al., 2017). Cerebral blood flow can be imaged with the SPECT tracers ^99m^Tc‐hexamethylpropylene amine oxime ([^99m^Tc]Tc‐HMPAO) and [^99m^Tc]‐ethyl cysteinate dimer ([^99m^Tc]Tc‐ECD) as well as with the PET tracer [^15^O]H_2_O (Ma et al., 2011). Oxygen consumption, cerebral blood flow and interregional connectivity can be imaged by fMRI (Pyatigorskaya et al., 2014). Like fMRI, PET measurements of energy consumption and blood flow can also be converted into measurements of function connectivity between brain areas (Watabe & Hatazawa, 2019). Brain functioning can be investigated not only in resting state, but also while performing tasks that involve brain areas implicated in PD (for reviews, see Herz et al., 2014; Weingarten et al., 2015).

Metabolic imaging with [^18^F]FDG revealed so‐called PD‐related metabolic patterns (PDRP) in patients with PD and atypical PS (Tang et al., 2010; Teune et al., 2013). In particular, the pattern related to idiopathic PD was characterized by increased metabolic activity in the pallidum/thalamus and pons/cerebellum and decreased metabolic activity in the premotor cortex, supplementary motor area, and parietal association regions (relative to the “healthy” reference pattern) (Ma et al., 2007). MSA‐related pattern was characterized by metabolic reductions on both sides of the putamen and in the cerebellum, while the PSP‐related pattern was characterized by decreased metabolic activity in the upper brainstem, medial frontal cortex, and medial thalamus (Eckert et al., 2008). Differences in metabolic patterns were large enough to distinguish between PD, MSA, and PSP patients on an individual level by assessing the similarity between each patient's [^18^F]FDG scan and the three disease‐specific patterns (Tang et al., 2010). Similar patterns have been detected in cerebral blood flow with [^99m^Tc]Tc‐ECD SPECT and [^15^O]H_2_O PET (Eckert et al., 2007; Ma et al., 2007).

Resting‐state fMRI imaging using arterial spin labeling or BOLD signal also revealed differences in patterns of oxygen consumption/blood flow between PD patients and healthy controls (Ma, Huang, et al., 2010; Melzer et al., 2011; Wu et al., 2015). Functional connectivity studies in PD patients showed a remapping of connections between striatal and cortical regions of the brain (Helmich et al., 2010).

Partial normalization of PD‐related patterns of cerebral energy consumption, blood flow, and connectivity was demonstrated by [^99m^Tc]Tc‐ECD SPECT, [^18^F]FDG PET, [^15^O]H_2_O PET, and fMRI in patients undergoing DBS or L‐DOPA treatments (Asanuma et al., 2006; Geday et al., 2009; Hirano et al., 2008; Vo et al., 2017; Wielepp et al., 2001).

Although [^18^F]FDG PET and fMRI have demonstrated the ability to distinguish between PD patients and healthy controls (Figure 3B,C), as well as between PD patients and patients with MSA and PSP on an individual level (Matthews et al., 2018; Tang et al., 2010; Wu et al., 2015), these methods are not used routinely for (differential) PD diagnostics, and their use is not standardized (Walker et al., 2018).

#### 2.3.7 Microglia

Microglia are macrophage‐like cells, which are components of the immune system in the brain (Bachiller et al., 2018; Table 1, row 18). Microglia become activated in neuroinflammation, which follows or accompanies brain injury and neurodegeneration. Post‐mortem studies in PD patients revealed high numbers of activated microglia in the SN (Hirsch et al., 2003). Activated microglia express higher levels of the mitochondrial translocator protein (TSPO) (Guilarte, 2019). Therefore, TSPO has been promoted as a biomarker for neuroinflammation (Dimitrova‐Shumkovska et al., 2020).

The extent of neuroinflammation in PD patients has been studied with TSPO PET tracers (*R*)‐[^11^C]PK11195 (Gerhard et al., 2006; Ouchi et al., 2005), [^11^C]PBR28 (Varnäs et al., 2019) and [^18^F]FEPPA (Ghadery et al., 2017). Initial studies with the first‐generation TSPO tracer (*R*)‐[^11^C]PK11195 showed higher tracer uptake in certain brain regions (midbrain, pons, cortex) of PD patients compared to healthy controls, which corroborated the hypothesis of neuroinflammation taking place in the PD‐affected brain (Gerhard et al., 2006; Ouchi et al., 2005). One study even found a correlation between (*R*)‐[^11^C]PK11195 uptake and motor symptoms of PD (Ouchi et al., 2005). However, follow‐up studies with second‐generation tracers [^11^C]PBR28 and [^18^F]FEPPA failed to reveal any differences between PD patients and healthy controls (Ghadery et al., 2017; Varnäs et al., 2019). Moreover, it was found that the polymorphism of the TSPO gene carried by the test subjects had more influence on TSPO tracer uptake in the brain than their PD status (Dimitrova‐Shumkovska et al., 2020). Therefore, the value of microglial imaging in PD remains unconfirmed.

#### 2.3.8 Other targets

##### Adenosine A_2A_ receptors (A_2A_R)

A_2A_R are one of the four adenosine receptor subtypes: A_1_, A_2A_, A_2B_, and A_3_ (Table 1, row 19). There are two rationales for the imaging of these receptors in PD. First, these receptors expressed in striatopallidal neurons and are known to heteromerize with D_2_ receptors, so they may be involved in the degeneration of dopaminergic pathways in PD (Pinna et al., 2018). Second, they are over‐expressed in activated microglia and are considered an alternative to TSPO as a target for imaging neuroinflammation (Tronel et al., 2017).

A_2A_R can be imaged with PET tracers such as [^11^C]SCH442416, [7‐methyl‐^11^C]‐(*E*)‐8‐(3,4,5‐trimethoxystyryl)1,3,7‐trimethylxanthine ([^11^C]TMSX) and [^11^C]preladenant (Grachev et al., 2014; Tronel et al., 2017). Studies with the former two tracers showed elevated availability of A_2A_R in the striatum of PD patients with dyskinesia, but not in dyskinesia‐free PD patients (Mishina et al., 2011; Ramlackhansingh et al., 2011). This increased binding is thought to be related to neuronal regulation rather than neuroinflammation (Mishina et al., 2011). [^11^C]Preladenant has been used for drug occupancy studies at the A_2A_Rs in the development of new A_2A_R‐targeting anti‐parkinsonian drugs (Ishibashi et al., 2018).

##### Type 1 cannabinoid receptors (CB1)

Endocannabinoids are modulators of neurotransmitter release: unlike neurotransmitters, they are synthesized on the postsynaptic side and activate cannabinoid receptors on the presynaptic membrane (Casteels et al., 2014; Table 1 row 20). Cannabinoid receptors are divided into type 1 (CB1) and type 2 (CB2). Most cannabinoid receptors in the brain belong to the CB1 type and are enriched in striatopallidal neurons (Cilia, 2018). CB1 receptors can be imaged with PET tracers [^18^F]FMPEP‐d_2_, [^11^C]OMAR, and [^18^F]MK‐9470 (Casteels et al., 2014).

Postmortem studies in PD patient brains show an up‐regulation of CB1 receptors in striatal regions (Lastres‐Becker et al., 2001). Treatment with CB1 receptor agonist may alleviate L‐DOPA induced dyskinesia (LID) in PD (Sieradzan et al., 2001). A PET imaging study using [^18^F]MK‐9470 in PD patients with and without LID showed increased CB1 availability in the putamen (a subsection of the striatum) of PD patients compared to healthy controls (Van Laere et al. 2012). However, no statistical association could be found between CB1 availability and the presence of LID.

##### *N*‐methyl‐d‐aspartate receptor (NMDAR)

NMDARs are a family of ionotropic receptors (ion channels) activated by glutamate—the principal excitatory neurotransmitter in the brain (Fuchigami et al., 2021). Overactive glutamatergic neurotransmission is thought to underlie LID, and inhibition of NMDARs reduces LID in preclinical PD models (Niccolini et al., 2015; Table 1, row 21). The availability of NMDAR in PD patients was imaged by PET using the noncompetitive NMDAR antagonist [^11^C]CNS5161 (Ahmed et al., 2011). The uptake of the tracer did not differ between LID‐free PD patients and healthy controls. However, the uptake in striatal and cortical regions was higher in patients with LID after L‐DOPA medication compared to patients without dyskinesia, which corroborates the hypothesis that dysregulated glutamate signaling is one of the causes of LID.

##### Phosphodiesterase enzymes

Phosphodiesterase's (PDE1‐11) are a family of intracellular enzymes that hydrolyze cyclic adenosine monophosphate (cAMP) and cyclic guanosine monophosphate (cGMP)—secondary messengers formed in the cell after the activation of GPCRs (Ignacio Andres et al. 2012; Table 1, row 22). Many neurotransmitters (dopamine, norepinephrine, serotonin, acetylcholine, glutamate, and others) bind to GPCRs, so PDEs play a role in modulating neurotransmission. There are PET tracers for PDE types 2, 4, 5, 7 and 10, and tracers for PDE2, 4, and 10 have been translated to humans (see (McCluskey et al., 2020; Ignacio Andres et al. 2012) for reviews).

The binding of the PDE4 tracer [^11^C]rolipram was decreased in the striatal, thalamic, and cortical regions of PD patients, and the decrease correlated with the degree of impairment in spatial working memory (Niccolini et al., 2017). Likewise, the binding of the PDE10A tracer [^11^C]IMA107 was decreased in the striatal regions of PD patients, and the decrease correlated with PD duration and severity of motor symptoms and complications (Niccolini, Foltynie, et al., 2015).

##### Neuromelanin

Neuromelanin is a dark polymeric pigment found in large quantities in the catecholaminergic cells of SN and LC. Neuromelanin can bind metal ions (e.g., iron) and harmful products of dopamine oxidation, thereby playing a protective role in dopaminergic neurons (Haining & Achat‐Mendes, 2017; Table 1, row 23). On the other hand, preclinical research in PD models shows that over‐accumulation of neuromelanin in cells leads to neurodegeneration (Vila, 2019).

PET imaging of neuromelanin in PD has been performed with the tracer [^18^F]AV1451, originally developed for tau tangles. Of the three published PET studies, two detected a lower [^18^F]AV1451 uptake in the SN of PD patients compared to healthy controls (Coakeley et al., 2018; Hansen et al., 2016), while in the third study the SN uptake was only significantly decreased in PD patients with dementia (Smith et al., 2018). The uptake of [^18^F]AV1451 in the SN was also decreased in PSP patients (Coakeley et al., 2018).

Iron ions bind to neuromelanin and form a paramagnetic complex that can be detected by MRI (Sulzer et al., 2018). Multiple MRI studies have found consistent decrease of neuromelanin‐specific MRI signal in the SN and LC of PD patients compared to healthy controls (see (Sulzer et al., 2018) for a review). A within‐subject comparison of neuromelanin MRI and DAT SPECT (with [^123^I]FP‐β‐CIT) showed that neuromelanin MRI could predict motor complications such as LIDs in PD patients, while DAT SPECT could not (Okuzumi et al., 2019). However, the sensitivity of neuromelanin MRI still does not permit diagnosis of PD on an individual level.

##### Tau and beta‐amyloid aggregates

Tau is a protein that regulates microtubule‐based intracellular transport (Lei et al., 2010; Table 1, rows 24 and 25). Beta‐amyloid is a peptide cleaved from the amyloid precursor protein (APP), a multifunctional protein involved in neuronal growth regulation (Lim et al., 2019; Thinakaran & Koo, 2008). Hyperphosphorylated tau aggregates and beta‐amyloid peptide fibrils are well known to be the hallmarks of AD (Uzuegbunam et al., 2020; Wilson et al., 2020), while idiopathic PD is not characterized by increased tau or beta‐amyloid load (Winer et al., 2018). However, evidence from postmortem examinations shows that DLB patients and PD patients with dementia have accumulation of beta‐amyloid in the striatum (Lim et al., 2019), while PSP patients have tau aggregates in multiple regions of the brain (Williams & Lees, 2009; Zhang et al., 2018). Therefore, the imaging of beta‐amyloid and tau aggregates is relevant for atypical PS.

Four most popular PET tracers for beta‐amyloid imaging are [^11^C]‐Pittsburgh compound B([^11^C]PIB), [^18^F]florbetaben, [^18^F]florbetapir, and [^18^F]flutemetamol, of which the latter three are approved for use in AD diagnosis in the US and Europe (Jovalekic et al. 2017; Uzuegbunam et al., 2020). The PET tracer [^18^F]AV1451 has recently been approved in the US for tau imaging in AD (Jie et al., 2021).

[^11^C]PIB showed elevated tracer uptake in the cortex of DLB patients compared to PD patients (Edison et al., 2008). Increased [^11^C]PIB uptake appears to be associated with the presence of cognitive dysfunction in PD, but different studies provide conflicting evidence (see Petrou et al., 2015; Saeed et al., 2020) for reviews). Increased uptake of [^18^F]AV1451 was found in the globus pallidus, putamen, subthalamic nucleus, midbrain, and dentate nucleus of PSP patients relative to PD patients (Schonhaut et al., 2017). The uptake of another tau tracer, [^18^F]FDDNP, was also significantly elevated in subthalamic area, midbrain, and cerebellar white matter compared to PD patients (Kepe et al., 2013). Therefore, beta‐amyloid and tau imaging may in the future become useful for differentiating PD from DLB and PSP, respectively.

## 3 NEW TARGETS FOR THE IMAGING OF PARKINSON'S DISEASE PATHOGENESIS

### 3.1 Alpha‐synuclein (α‐Syn)

Oligomerization and aggregation of α‐Syn seem to be the first step in the cascade of PD pathogenesis eventually leading to neuronal death and the spread of misfolded α‐Syn across the central and peripheral nervous systems (see Section 1.2.1). Therefore, there is virtually universal agreement that the appearance of effective molecular imaging agents for α‐Syn will transform PD research and diagnostics (Eberling et al., 2013; Helmich et al., 2018; Kotzbauer et al., 2017; Saeed et al., 2020; Strafella et al., 2017; Weingarten et al., 2015). Imaging of α‐Syn would allow clinicians to spot pathological alterations in the brain long before PD symptoms develop, follow the progression of the disease and assess the efficacy of novel anti‐PD treatments, especially treatments that target α‐Syn itself.

Currently, no radiotracers are available for α‐Syn imaging in humans. This is due to several specific challenges of α‐Syn as an imaging target (Eberling et al., 2013; Kotzbauer et al., 2017). First, the abundance of α‐Syn in the brain is low, both in absolute terms and relative to other misfolded proteins associated with neurodegeneration, such as tau and beta‐amyloid. Therefore, an effective α‐Syn tracer must have both an extremely high affinity and high selectivity toward α‐Syn fibrils. Second, most of the α‐Syn is located intracellularly, which means that the tracer has to pass both the BBB and cell membranes before it can bind. Third, α‐Syn does not have a specific structure and can undergo various post‐translational modifications such as methionine sulfoxidation, tyrosine nitration, and serine phosphorylation (Anderson et al., 2006; Schildknecht et al., 2013). Modified forms of α‐Syn differ in their cytotoxicity, solubility, and propensity to form oligomers and fibrils. This heterogeneity of α‐Syn makes it difficult to establish in vitro assays to optimize lead compounds (Kotzbauer et al., 2017). It should also be noted that initial in vivo evaluation of candidate tracers for brain imaging is usually performed in rodents, and there are well‐described discrepancies between BBB permeability in rodents compared to larger animals including humans (Shalgunov et al., 2020; Syvänen et al., 2009).

**FIGURE 4:**
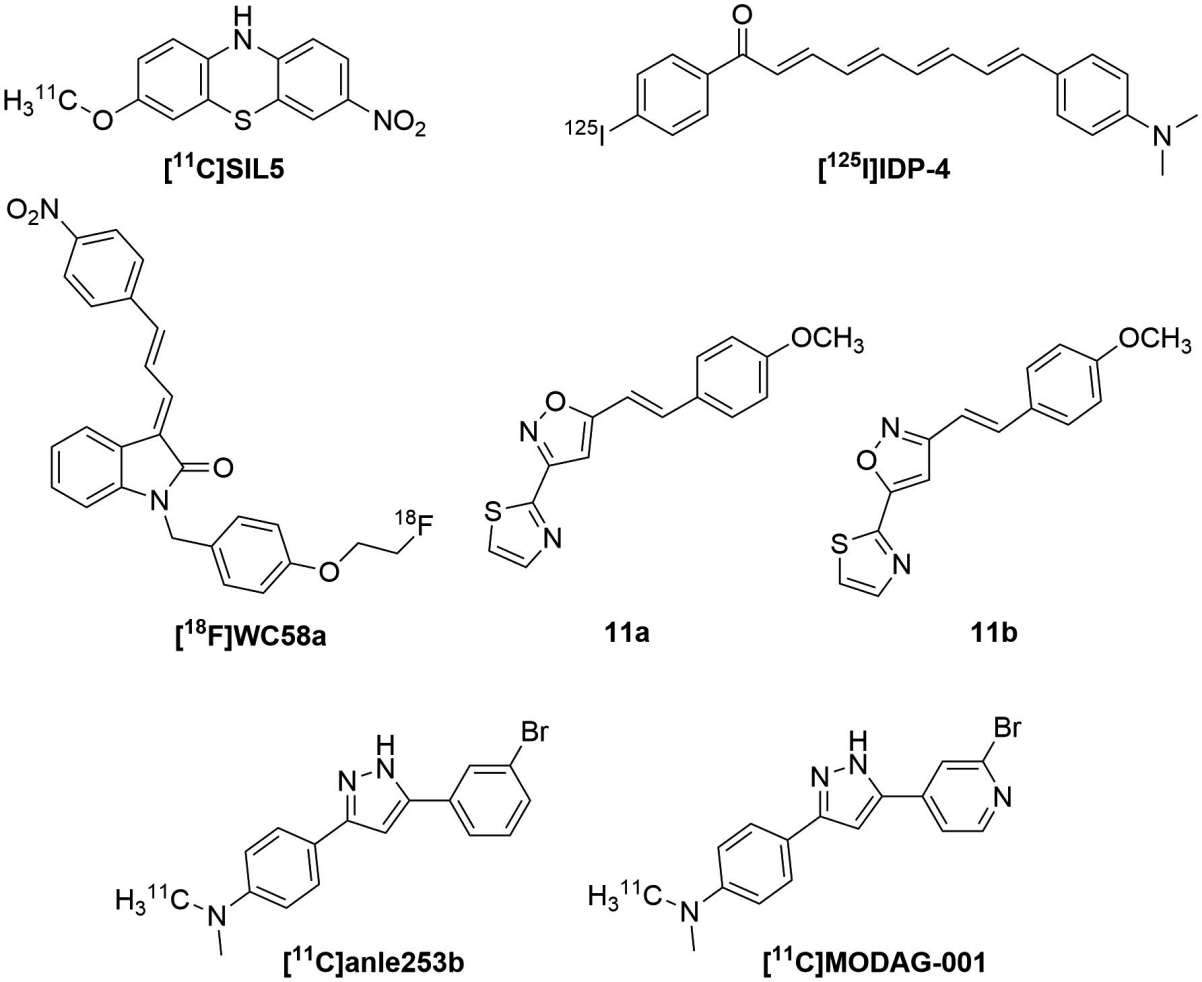
The most promising candidate structures for α‐Syn tracer development

Lead compounds from several classes (Figure 4) are currently being optimized by various research groups to obtain a PET tracer for α‐Syn (Jovalekic et al., 2017; Korat et al., 2021; Kotzbauer et al., 2017; Uzuegbunam et al., 2020). In silico docking studies they showed that these compounds bind to different subsets of binding sites present on α‐Syn fibrils (Hsieh, Ferrie, et al., 2018). Phenothiazine [^11^C]SIL5 with an affinity of ~30 nM toward α‐Syn showed good brain penetration in rats and macaques, but its affinity is considered too low for α‐Syn imaging in humans (Bagchi et al., 2013; Zhang et al., 2014). Optimization of the indolinone scaffold resulted in the development of [^18^F]WC58a with α‐Syn affinity of 9 nM (Chu et al., 2015). However, this compound was judged too lipophilic to be evaluated in vivo. Chalcone derivative IDP‐4, with a 5 nM affinity toward α‐Syn, was labeled with iodine‐125 and evaluated in mice (Ono et al., 2016). It showed low brain uptake and slow clearance, apparently due to its extremely apolar structure. Further optimization of the chalcone scaffold generated more polar compounds 11a and 11b (Figure 4), which had α‐Syn affinity of 18.5 nM (Hsieh, Xu, et al., 2018). These compounds are used as lead structures for further structure activity relationship (SAR) studies.

Pyrazole derivatives [^11^C]anle253 and [^11^C]MODAG‐001 have shown the most promising results so far (Kuebler et al., 2020; Maurer et al., 2020). in vitro, they demonstrated very high affinities toward α‐Syn fibrils (IC_50_ 1.6 nM and *K*_d_ 0.6 nM, respectively), higher than all other candidate of α‐Syn ligands previously reported. [^11^C]MODAG‐001 also showed 30‐fold preference for α‐Syn over tau and beta‐amyloid fibrils. PET experiments in rats confirmed that both [^11^C]anle253 and [^11^C]MODAG‐001 had suitable brain penetration and washout kinetics. A methyl group in [^11^C]MODAG‐001 structure was deuterated to diminish radiometabolite formation, giving (d_3_)‐[^11^C]MODAG‐001. In subsequent (d_3_)‐[^11^C]MODAG‐001 evaluation, recombinant α‐Syn fibrils were inoculated into the striatum, and PET images showed the accumulation of the tracer at the inoculation site (Kuebler et al., 2020). However, tritiated [^3^H]MODAG‐001 failed to show any specific binding to α‐Syn in brain slices of patients with DLB (Kuebler et al., 2020). Possible explanations are insufficient α‐Syn affinity of the [^3^H]MODAG‐001 under the used assay conditions, high non‐specific binding of the radioligand or structural differences between α‐Syn fibrils present in the brain slices and used for screening assays.

### 3.2 Leucine‐rich repeat kinase 2 (LRRK2)

LRRK2 is a large protein with kinase and GTPase activity, which is localized in synaptic vesicles and mitochondrial membranes in mammalian brain (Biskup et al., 2006). LRRK2 mutations, especially those with increased kinase activity, are associated with familial PD forms (Estrada & Sweeney, 2015; Zimprich et al., 2004). LRRK2‐associated PD closely resembles idiopathic PD in its symptoms and response to treatment (Tolosa et al., 2020), but some features, for example, the change in brain AChE activity (Liu et al., 2018), are different. Therefore, investigating LRRK2 as a biomarker and a therapeutic target is a promising strategy both for personalized and general PD treatment. The development and evaluation of brain penetrant LRRK2 inhibitors for PD therapy is underway (Chen et al., 2018; Estrada & Sweeney, 2015).

Several LRRK2 inhibitors have been labeled with PET isotopes for use as tracers (Rideout et al., 2020). These include [^11^C]HG‐10‐102‐01, [^11^C]GNE‐1023, and [^18^F]FIPM (Chen, Shao, et al., 2019; Mori et al., 2020; Wang et al., 2017). In vivo evaluation of [^11^C]HG‐10‐102‐01 has not yet been reported, while [^11^C]GNE‐1023 and [^18^F]FIPM failed to show sufficient specific signal in preclinical studies (Chen, Shao, et al., 2019). Given the low expected density (<1 nM) of LRRK2 expression in the brain (Rideout et al., 2020), development of nuclear imaging agents for this target will be challenging. However, if a working tracer is developed, the benefits for the understanding of PD pathophysiology can be immense.

### 3.3 Mitochondrial complex I

Mitochondrial complex I is the first component in the respiratory electron transport chain, which participates in adenosine triphosphate (ATP) synthesis by oxidative phosphorylation in mitochondria (Hirst, 2013). A deficiency in the mitochondrial complex I has been found in the SN neurons in PD (Chen et al., 2019; Hauser & Hastings, 2013). The PET tracer 2‐*tert*‐butyl‐4‐chloro‐5‐{6‐[2‐(2‐^18^F‐fluoroethoxy)‐ethoxy]‐pyridin‐3‐ylmethoxy}‐2*H*‐pyridazin‐3‐one ([^18^F]BCPP‐EF) demonstrated the ability to detect changes in mitochondrial complex‐I activity in the brains of MPTP‐treated parkinsonic monkeys (Tsukada et al., 2016). [^18^F]BCPP‐EF has recently been translated into humans (Mansur et al., 2020, 2021), so imaging studies investigating mitochondrial dysfunction in PD patients may appear soon.

### 3.4 Histone deacetylases (HDACs)

HDAC enzymes are involved in histone (de)acetylation, which is one of the mechanisms of epigenetic modification of the DNA (Hegarty et al., 2016). Alterations in histone acetylation have been demonstrated both in preclinical models of PD and in dopaminergic neurons of PD patients (Park et al., 2016) and may be a consequence of α‐Syn neurotoxicity (Harrison & Dexter, 2013). HDAC inhibitors are being investigated as anti‐PD drugs (Hegarty et al., 2016). Therefore, HDACs have potential as new imaging biomarkers for PD. Several PET tracers for HDACs have been developed and evaluated in rodents and non‐human primates in recent years, but only one tracer, [^11^C]Martinostat, selective for class I HDACs (isoforms 1, 2, and 3), has been tested in humans (Tago & Toyohara, 2018; Wey et al., 2016). Alterations of HDAC levels measured by [^11^C]Martinostat were recently found in AD and bipolar disorder patients (Pascoal et al., 2018; Tseng et al., 2020), but no imaging studies with [^11^C]Martinostat in PD patients have so far been reported.

## 4 DISCUSSION AND CONCLUSION

PD is a complex disease, the etiology of which remains incompletely understood. Therefore, diagnosing the disease at an early stage and preventing its development is difficult. PD diagnosis is currently based on the history of motor symptoms, while the role of molecular imaging methods remains auxiliary (Armstrong & Okun, 2020). However, molecular imaging is very important for PD research because it allows the investigation of molecular mechanisms underlying this disease in both patients and animal models. This aids the development and evaluation of new anti‐PD treatments and can potentially allow neurologists to diagnose PD before symptoms develop. Molecular imaging has helped clinicians to uncover the multifaceted nature of PD and led to the current understanding that PD is—very likely—not a single disease but rather a heterogeneous family of disorders with similar symptoms (Armstrong & Okun, 2020; Von Coelln and Shulman 2016; Cropley et al., 2008).

Molecular imaging methods used to study PD include SPECT, PET, and MRI. PET, and SPECT tracers can be tailored to provide information about specific molecular targets. Therefore, nuclear imaging can be used to detect minor alterations in the molecular machinery of the brain.

SPECT and PET tracers that target the dopaminergic system of the brain ([^18^F]F‐DOPA, [^123^I]FP‐β‐CIT) and the noradrenergic cardiac innervation ([^123^I]MIBG) are used to assist PD diagnosis (Orimo et al., 2012; Puñal‐Riobóo et al., 2009; Roussakis et al., 2013). Imaging of cerebral flow and metabolism with PET ([^18^F]FDG, [^15^O]H_2_O) and fMRI has also proven useful in diagnosing PD and differentiating it from atypical PS (Walker et al., 2018; Wu et al., 2015). Tracers for non‐dopaminergic neurotransmitter receptors and enzymes involved in secondary messenger regulation have been used to investigate the origins of non‐motor symptoms of PD and assess target occupancy of drugs applied to treat those symptoms (Ballanger et al., 2010; Bohnen et al., 2007; Ishibashi et al., 2018; Niccolini, Foltynie, et al., 2015). Such studies should eventually result in the development of drugs for adjunct therapy of PD that would further improve the quality of life for PD patients.

However, to achieve a real breakthrough in PD research, diagnostics, and treatment, the mechanisms of PD pathogenesis during the asymptomatic phase have to be unraveled. PET imaging of DAT and VMAT2 has demonstrated that the dopaminergic connections in the brain start to degenerate years before PD symptoms develop (De La Fuente‐Fernández et al., 2011). There is an unmet need for tracers capable of imaging the cause of the neurodegeneration, rather than the neurodegeneration itself.

The most relevant imaging target in this regard is α‐Syn. Accumulation of α‐Syn is well known to be the hallmark of PD, while α‐Syn oligomers were shown to be neurotoxic (Kalia et al., 2013). However, α‐Syn is a very challenging imaging target (Eberling et al., 2013; Kotzbauer et al., 2017). Nevertheless, several classes of small‐molecule probes are currently being optimized and evaluated for the purpose of α‐Syn imaging, and some of them have shown promising results in vivo (Kuebler et al., 2020; Maurer et al., 2020).

Two other targets the imaging of which may help reveal the cause of neurodegeneration in PD are LRRK2 and mitochondrial complex I. LRRK2 dysfunction is associated with familial PD and possible with idiopathic PD as well (Zimprich et al., 2004). Mitochondrial deficiency is also a well‐known factor in the development of oxidative stress and neurotoxicity in PD (Hauser & Hastings, 2013). A first‐in‐human evaluation of a mitochondrial complex I tracer has recently been reported (Mansur et al., 2020, 2021). Tracers for LRRK2 are still in preclinical development. Histone (de)acetylation, although probably a consequence of α‐Syn toxicity or gene dysfunctions mentioned above, may also be a valid imaging biomarker in PD. One tracer for HDAC imaging, [^11^C]Martinostat, has already been translated into humans, and its use led to interesting findings in other neurodegenerative diseases (Pascoal et al., 2018).

It should be noted that there may be no single imaging target that could serve as a perfect PD biomarker (Schapira, 2013; Weingarten et al., 2015). Even α‐Syn accumulation is not strictly specific to PD, but also occurs in DLB and atypical PS (Eberling et al., 2013). This is where multimodal imaging, that is the comparison of findings from different imaging modalities, could help (Horsager et al., 2020; Perani et al., 2019).

Fundamental research into the mechanisms of PD pathogenesis would benefit from accelerated development of imaging agents for targets suspected to be involved in PD. The use of engineered antibodies actively transported over the BBB as imaging agents can be especially fruitful (Gee et al., 2020; Sehlin & Syvänen, 2019). Antibodies developed for passive immunotherapy of PD can be repurposed as imaging agents (Oertel & Schulz, 2016; Zella et al., 2019). The feasibility of this approach has recently been demonstrated preclinically with bispecific antibodies against beta‐amyloid, which were transported across the BBB by interacting with transferrin receptors on the luminal side of the cerebral vasculature (Sehlin et al., 2016). Although the slow pharmacokinetics of antibodies remains an obstacle to the clinical translation of antibody‐based imaging agents, the rapidly developing pretargeting strategies can solve this problem soon (Altai et al., 2017; Rossin et al., 2010; Stéen et al., 2018; Zeglis et al., 2013).

## CONFLICT OF INTERESTS

The authors declare no conflict of interests.

## AUTHOR CONTRIBUTION

Conceptualization (lead) : MMH. Original draft preparation, review and editing (equal contribution): NB and IVA. Review and editing: VS (lead), MMH and ADW. All authors read and approved the final manuscript.

## Data Availability

Data sharing is not applicable to this Review article as no new datasets were generated during the current study.

## References

[jnc15516-bib-0001] Abbasi Gharibkandi, N. , & Hosseinimehr, S. J. (2019). Radiotracers for imaging of Parkinson's disease. European Journal of Medicinal Chemistry, 166, 75–89. 10.1016/j.ejmech.2019.01.029. 30685535

[jnc15516-bib-0002] Ahmed, I. , Bose, S. K. , Pavese, N. , Ramlackhansingh, A. , Turkheimer, F. , Hotton, G. , Hammers, A. , & Brooks, D. J. (2011). Glutamate NMDA receptor dysregulation in Parkinson’s disease with dyskinesias. Brain, 134, 979–986. 10.1093/brain/awr028. 21371994

[jnc15516-bib-0003] Altai, M. , Membreno, R. , Cook, B. , Tolmachev, V. , & Zeglis, B. M. (2017). Pretargeted imaging and therapy. Journal of Nuclear Medicine, 58, 1553–1559. 28687600 10.2967/jnumed.117.189944 PMC5632733

[jnc15516-bib-0004] Ametamey, S. M. , Honer, M. , & Schubiger, P. A. (2008). Molecular imaging with PET. Chemical Reviews, 108, 1501–1516. 18426240 10.1021/cr0782426

[jnc15516-bib-0005] Andersen, K. B. , Hansen, A. K. , Damholdt, M. F. , Horsager, J. , Skjærbæk, C. , Gottrup, H. , Klit, H. , Schacht, A. C. , Danielsen, E. H. , Brooks, D. J. , & Borghammer, P. (2021). Reduced synaptic density in patients with lewy body dementia: An [^11^C] UCB‐J PET imaging study. Movement Disorders, 36, 2057–2065. 10.1002/mds.28617. 33899255

[jnc15516-bib-0006] Anderson, J. P. , Walker, D. E. , Goldstein, J. M. , de Laat, R. , Banducci, K. , Caccavello, R. J. , Barbour, R. , Huang, J. , Kling, K. , Lee, M. , Diep, L. , Keim, P. S. , Shen, X. , Chataway, T. , Schlossmacher, M. G. , Seubert, P. , Schenk, D. , Sinha, S. , Gai, W. P. , … Chilcote, T. J. (2006). Phosphorylation of Ser‐129 Is the dominant pathological modification of α‐synuclein in familial and sporadic lewy body disease. Journal of Biological Chemistry, 281, 29739–29752. 10.1074/jbc.m600933200. 16847063

[jnc15516-bib-0007] Angot, E. , Steiner, J. A. , Hansen, C. , Li, J.‐Y. , & Brundin, P. (2010). Are synucleinopathies prion‐like disorders? The Lancet Neurology, 9, 1128–1138. 20846907 10.1016/S1474-4422(10)70213-1

[jnc15516-bib-0008] Antonini, A. , Leenders, K. L. , Vontobel, P. , Maguire, R. P. , Missimer, J. , Psylla, M. , & Günther, I. (1997). Complementary PET studies of striatal neuronal function in the differential diagnosis between multiple system atrophy and Parkinson’s disease. Brain, 120, 2187–2195. 10.1093/brain/120.12.2187. 9448574

[jnc15516-bib-0009] Antonini, A. , Tinazzi, M. , Abbruzzese, G. , Berardelli, A. , Chaudhuri, K. R. , Defazio, G. , Ferreira, J. , Martinez‐Martin, P. , Trenkwalder, C. , & Rascol, O. (2018). Pain in Parkinson’s disease: Facts and uncertainties. European Journal of Neurology, 25, 917–924. 10.1111/ene.13624. 29520899

[jnc15516-bib-0010] Appel, L. , Jonasson, M. , Danfors, T. , Nyholm, D. , Askmark, H. , Lubberink, M. , & Sorensen, J. (2015). Use of ^11^C‐PE2I PET in differential diagnosis of parkinsonian disorders. Journal of Nuclear Medicine, 56, 234–242. 25593112 10.2967/jnumed.114.148619

[jnc15516-bib-0011] Aquilonius, S.‐M. , Bertröm, K. , Eckernäs, S‐Å. , Hartvig, P. , Leenders, K.L. , Lundquist, H. , Antoni, G. , Gee, A. , Rimland, A. , Uhlin, J. , & Långström, B. (1987). In vivo evaluation of striatal dopamine reuptake sites using 11C‐nomifensine and positron emission tomography. Acta Neurologica Scandinavica, 76, 283–287. 10.1111/j.1600-0404.1987.tb03582.x. 2961191

[jnc15516-bib-0012] Armstrong, M. J. , & Okun, M. S. (2020). Diagnosis and treatment of Parkinson disease. JAMA, 323, 548. 10.1001/jama.2019.22360. 32044947

[jnc15516-bib-0013] Asahina, M. , Shinotoh, H. , Hirayama, K. , Suhara, T. , Shishido, F. , Inoue, O. , & Tateno, Y. (1995). Hypersensitivity of cortical muscarinic receptors in Parkinson’s disease demonstrated by PET. Acta Neurologica Scandinavica, 91, 437–443. 7572037 10.1111/j.1600-0404.1995.tb00443.x

[jnc15516-bib-0014] Asanuma, K. , Tang, C. , Ma, Y. , Dhawan, V. , Mattis, P. , Edwards, C. , Kaplitt, M. G. , Feigin, A. , & Eidelberg, D. (2006). Network modulation in the treatment of Parkinson’s disease. Brain, 129, 2667–2678. 10.1093/brain/awl162. 16844713 PMC4459513

[jnc15516-bib-0015] Bachiller, S. , Jiménez‐Ferrer, I. , Paulus, A. , Yang, Y. , Swanberg, M. , Deierborg, T. , & Boza‐Serrano, A. (2018). Microglia in neurological diseases: A road map to brain‐disease dependent‐inflammatory response. Frontiers in Cellular Neuroscience, 12, 488. 30618635 10.3389/fncel.2018.00488 PMC6305407

[jnc15516-bib-0016] Bagchi, D. P. , Yu, L. , Perlmutter, J. S. , Xu, J. , Mach, R. H. , Tu, Z. , & Kotzbauer, P. T. (2013). Binding of the radioligand SIL23 to α‐synuclein fibrils in Parkinson disease brain tissue establishes feasibility and screening approaches for developing a Parkinson disease imaging agent. PLoS One, 8, e55031. 10.1371/journal.pone.0055031. 23405108 PMC3566091

[jnc15516-bib-0017] Balestrino, R. , & Schapira, A. H. V. (2020). Parkinson disease. European Journal of Neurology, 27, 27–42. 31631455 10.1111/ene.14108

[jnc15516-bib-0018] Ballanger, B. , Klinger, H. , Eche, J. , Lerond, J. , Vallet, A.‐E. , Bars, D. , Le, T. L. , Sgambato‐Faure, V. , Broussolle, E. , & Thobois, S. (2012). Role of serotonergic 1A receptor dysfunction in depression associated with Parkinson’s disease. Movement Disorders, 27, 84–89. 10.1002/mds.23895. 21994070

[jnc15516-bib-0019] Ballanger, B. , Strafella, A. P. , Eimeren, T. , van Zurowski, M. , Rusjan, P. M. , Houle, S. , & Fox, S. H. (2010). Serotonin 2A receptors and visual hallucinations in Parkinson disease. Archives of Neurology, 67, 416–421. 20385906 10.1001/archneurol.2010.35

[jnc15516-bib-0020] Barker, R. A. , Drouin‐Ouellet, J. , & Parmar, M. (2015). Cell‐based therapies for Parkinson disease‐past insights and future potential. Nature Reviews Neurology, 11, 492–503. 26240036 10.1038/nrneurol.2015.123

[jnc15516-bib-0021] Beaulieu, J.‐M. , Espinoza, S. , & Gainetdinov, R. R. (2015). Dopamine receptors ‐ IUPHAR Review 13. British Journal of Pharmacology, 172, 1–23. 25671228 10.1111/bph.12906 PMC4280963

[jnc15516-bib-0022] Beer, A. , Kessler, H. , Wester, H.‐J. , & Schwaiger, M. (2011). PET imaging of integrin αVβ3 expression. Theranostics, 1, 48–57. 10.7150/thno/v01p0048. 21547152 PMC3086612

[jnc15516-bib-0023] Benabid, A. L. , Pollak, P. , Gross, C. , Hoffmann, D. , Benazzouz, A. , Gao, D. M. , Laurent, A. , Gentil, M. , & Perret, J. (1994). Acute and long‐term effects of Subthalamic nucleus stimulation in Parkinson’s disease. Stereotactic and Functional Neurosurgery, 62, 76–84. 10.1159/000098600. 7631092

[jnc15516-bib-0024] Benamer, H. T. S. , Patterson, J. , Wyper, D. J. , Hadley, D. M. , Macphee, G. J. A. , & Grosset, D. G. (2000). Correlation of Parkinson’s disease severity and duration with ^123^I‐FP‐CIT SPECT striatal uptake. Movement Disorders, 15, 692–698. 10.1002/1531-8257(200007)15:4<692:AID-MDS1014>3.0.CO;2-V. 10928580

[jnc15516-bib-0025] Berding, G. , Schrader, C. , Peschel, T. , Hoff, J. , & van den Kolbe, H. , Meyer, G. , Dengler, R. , & Knapp, W. (2003). [N‐methyl ^11^C]meta‐Hydroxyephedrine positron emission tomography in Parkinson’s disease and multiple system atrophy. European Journal of Nuclear Medicine and Molecular Imaging, 30, 127–131. 12483420 10.1007/s00259-002-1019-7

[jnc15516-bib-0026] Biskup, S. , Moore, D. J. , Celsi, F. , Higashi, S. , West, A. B. , Andrabi, S. A. , Kurkinen, K. , Yu, S.‐W. , Savitt, J. M. , Waldvogel, H. J. , Faull, R. L. M. , Emson, P. C. , Torp, R. , Ottersen, O. P. , Dawson, T. M. , & Dawson, V. L. (2006). Localization of LRRK2 to membranous and vesicular structures in mammalian brain. Annals of Neurology, 60, 557–569. 10.1002/ana.21019. 17120249

[jnc15516-bib-0027] Blin, J. , Sette, G. , Fiorelli, M. , Bletry, O. , Elghozi, J. L. , Crouzel, C. , & Baron, J. C. (1990). A method for the in vivo investigation of the serotonergic 5‐HT_2_ receptors in the human cerebral cortex using positron emission tomography and ^18^F‐labeled setoperone. Journal of Neurochemistry, 54, 1744–1754. 2182776 10.1111/j.1471-4159.1990.tb01229.x

[jnc15516-bib-0028] Bohnen, N. I. , Kanel, P. , & Müller, M. L. T. M. (2018). Molecular imaging of the cholinergic system in Parkinson’s disease. International Review of Neurobiology, 141, 211–250. 30314597 10.1016/bs.irn.2018.07.027 PMC6218162

[jnc15516-bib-0029] Bohnen, N. I. , Kaufer, D. I. , Hendrickson, R. , Constantine, G. M. , Mathis, C. A. , & Moore, R. Y. (2007). Cortical cholinergic denervation is associated with depressive symptoms in Parkinson’s disease and parkinsonian dementia. Journal of Neurology, Neurosurgery and Psychiatry, 78, 641–643. 17507447 10.1136/jnnp.2006.100073 PMC2077949

[jnc15516-bib-0030] Boileau, I. , Guttman, M. , Rusjan, P. , Adams, J. R. , Houle, S. , Tong, J. , Hornykiewicz, O. , Furukawa, Y. , Wilson, A. A. , Kapur, S. , & Kish, S. J. (2009). Decreased binding of the D_3_ dopamine receptor‐preferring ligand [^11^C]‐(+)‐PHNO in drug‐naive Parkinson’s disease. Brain, 132, 1366–1375. 10.1093/brain/awn337. 19153147

[jnc15516-bib-0031] Bonifati, V. (2003). Mutations in the DJ‐1 gene associated with autosomal recessive early‐onset parkinsonism. Science, 299, 256–259. 10.1126/science.1077209. 12446870

[jnc15516-bib-0032] Braak, H. , Tredici, K. , Del, R. U. , De, V. R. A. I. , Jansen, S. E. , & Braak, E. (2003). Staging of brain pathology related to sporadic Parkinson’s disease. Neurobiology of Aging, 24, 197–211. 12498954 10.1016/s0197-4580(02)00065-9

[jnc15516-bib-0033] Broussolle, E. , Dentresangle, C. , Landais, P. , Garcia‐Larrea, L. , Pollak, P. , Croisile, B. , Hibert, O. , Bonnefoi, F. , Galy, G. , Froment, J.C. , & Comar, D. (1999). The relation of putamen and caudate nucleus ^18^F‐Dopa uptake to motor and cognitive performances in Parkinson’s disease. Journal of the Neurological Sciences, 166, 141–151. 10.1016/s0022-510x(99)00127-6. 10475108

[jnc15516-bib-0034] Brundin, P. , Dave, K. D. , & Kordower, J. H. (2017). Therapeutic approaches to target alpha‐synuclein pathology. Experimental Neurology, 298, 225–235. 28987463 10.1016/j.expneurol.2017.10.003 PMC6541231

[jnc15516-bib-0035] Casteels, C. , Bormans, G. , & Van Laere, K. (2014). Brain Imaging of Cannabinoid Receptors, in Imaging Hum. Brain Heal. Dis., pp. 37–79. Elsevier.

[jnc15516-bib-0036] Chatani, E. , & Yamamoto, N. (2018). Recent progress on understanding the mechanisms of amyloid nucleation. Biophysical Reviews, 10, 527–534. 29214606 10.1007/s12551-017-0353-8 PMC5899713

[jnc15516-bib-0037] Chen, C. , Turnbull, D. M. , & Reeve, A. K. (2019). Mitochondrial dysfunction in Parkinson’s disease‐cause or consequence? Biology (Basel), 8, 38. 10.3390/biology8020038. PMC6627981 31083583

[jnc15516-bib-0038] Chen, J. , Chen, Y. , & Pu, J. (2018). Leucine‐rich repeat kinase 2 in Parkinson’s disease: Updated from pathogenesis to potential therapeutic target. European Neurology, 79, 256–265. 10.1159/000488938. 29705795

[jnc15516-bib-0039] Chen, J.‐F. , & Cunha, R. A. (2020). The belated US FDA approval of the adenosine A2A receptor antagonist istradefylline for treatment of Parkinson’s disease. Purinergic Signal, 16, 167–174. 10.1007/s11302-020-09694-2. 32236790 PMC7367999

[jnc15516-bib-0040] Chen, Z. , Shao, T. , Gao, W. , Fu, H. , Collier, T. L. , Rong, J. , Deng, X. , Yu, Q. , Zhang, X. , Davenport, A. T. , Daunais, J. B. , Wey, H.‐Y. , Shao, Y. , Josephson, L. , Qiu, W.‐W. , & Liang, S. (2019). Synthesis and preliminary evaluation of [^11^C]GNE‐1023 as a potent PET probe for imaging leucine‐rich repeat kinase 2 (LRRK2) in Parkinson’s disease. ChemMedChem, 14, 1580–1585. 10.1002/cmdc.201900321. 31365783 PMC6726558

[jnc15516-bib-0041] Cherry, S. R. , & Dahlbom, M. (2006). PET: physics, instrumentation, and scanners, (Phelps M. E., ed). Springer New York.

[jnc15516-bib-0042] Chevalme, Y. M. , Montravers, F. , Vuillez, J. P. , Zanca, M. , Fallais, C. , Oustrin, J. , & Talbot, J. N. (2007). FDOPA‐(^18^F): A PET radiopharmaceutical recently registered for diagnostic use in countries of the European Union. Brazilian Archives of Biology and Technology, 50, 77–90.

[jnc15516-bib-0043] Chu, W. , Zhou, D. , Gaba, V. , Liu, J. , Li, S. , Peng, X. , Xu, J. , Dhavale, D. , Bagchi, D. P. , d’Avignon, A. , Shakerdge, N. B. , Bacskai, B. J. , Tu, Z. , Kotzbauer, P. T. , & Mach, R. H. (2015). Design, synthesis, and characterization of 3‐(Benzylidene)indolin‐2‐one derivatives as ligands for α‐synuclein fibrils. Journal of Medicinal Chemistry, 58, 6002–6017. 10.1021/acs.jmedchem.5b00571. 26177091 PMC4624220

[jnc15516-bib-0044] Cilia, R. (2018). Molecular imaging of the cannabinoid system in idiopathic Parkinson’s disease. International Review of Neurobiology, 141, 305–345. 30314601 10.1016/bs.irn.2018.08.004

[jnc15516-bib-0045] Coakeley, S. , Cho, S. S. , Koshimori, Y. , Rusjan, P. , Ghadery, C. , Kim, J. , Lang, A. E. , Houle, S. , & Strafella, A. P. (2018). [^18^F]AV‐1451 binding to neuromelanin in the substantia nigra in PD and PSP. Brain Structure and Function, 223, 589–595. 28884232 10.1007/s00429-017-1507-y

[jnc15516-bib-0046] Colloby, S. J. , Pakrasi, S. , Firbank, M. J. , Perry, E. K. , Piggott, M. A. , Owens, J. , Wyper, D. J. , McKeith, I. G. , Burn, D. J. , Williams, E. D. , & O’Brien, J. T. (2006). In vivo SPECT imaging of muscarinic acetylcholine receptors using (R, R) ^12^3I‐QNB in dementia with Lewy bodies and Parkinson’s disease dementia. NeuroImage, 33, 423–429. 10.1016/j.neuroimage.2006.07.026. 16959499

[jnc15516-bib-0047] Cropley, V. L. , Fujita, M. , Bara‐Jimenez, W. , Brown, A. K. , Zhang, X.‐Y. , Sangare, J. , Herscovitch, P. , Pike, V. W. , Hallett, M. , Nathan, P. J. , & Innis, R. B. (2008). Pre‐ and post‐synaptic dopamine imaging and its relation with frontostriatal cognitive function in Parkinson disease: PET studies with [^11^C]NNC112 and [^18^F]FDOPA. Psychiatry Res. ‐ Neuroimaging, 163, 171–182. 10.1016/j.pscychresns.2007.11.003. 18504119

[jnc15516-bib-0048] Darvesh, S. , Hopkins, D. A. , & Geula, C. (2003). Neurobiology of butyrylcholinesterase. Nature Reviews Neuroscience, 4, 131–138. 12563284 10.1038/nrn1035

[jnc15516-bib-0049] Dauer, W. , & Przedborski, S. (2003). Parkinson’s disease: Mechanisms and MODELS. Neuron, 39, 889–909. 10.1016/S0896-6273(03)00568-3. 12971891

[jnc15516-bib-0050] Davis, K. M. , Ryan, J. L. , Aaron, V. D. , & Sims, J. B. (2020). PET and SPECT imaging of the brain: History, technical considerations, applications, and radiotracers. Seminars in Ultrasound, CT and MRI, 41, 521–529. 10.1053/j.sult.2020.08.006. 33308491

[jnc15516-bib-0153] de la Fuente‐Fernández, R. , Schulzer, M. , Kuramoto, L. , Cragg, J. , Ramachandiran, N. , Au, W. L. , Mak, E. , McKenzie, J. , McCormick, S. , Sossi, V. , Ruth, T. J. , Lee, C. S. , Calne, D. B. , & Stoessl, A. J. (2011). Age‐specific progression of nigrostriatal dysfunction in Parkinson's disease. Annals of Neurology, 69, 803–810. 10.1002/ana.22284. 21246604

[jnc15516-bib-0051] DeLorenzo, C. , Kumar, J. D. , Zanderigo, F. , Mann, J. J. , Parsey, R. V. (2009). Modeling considerations for in vivo quantification of the dopamine transporter using [^11^C]PE2I and positron emission tomography. Journal of Cerebral Blood Flow and Metabolism, 29, 1332–1345. 19458606 10.1038/jcbfm.2009.49 PMC2757108

[jnc15516-bib-0052] Delva, A. , Weehaeghe, D. , Van, K. M. , Van, L. K. , & Vandenberghe, W. (2020). Loss of presynaptic terminal integrity in the substantia nigra in early Parkinson’s disease. Movement Disorders, 35, 1977–1986. 10.1002/mds.28216. 32767618

[jnc15516-bib-0053] Deutschländer, A. , la Fougère, C. , Boetzel, K. , Albert, N. L. , Gildehaus, F.‐J. , Bartenstein, P. , Xiong, G. , & Cumming, P. (2016). Occupancy of pramipexole (Sifrol) at cerebral dopamine D_2/3_ receptors in Parkinson’s disease patients. NeuroImage Clinical, 12, 41–46. 10.1016/j.nicl.2016.06.007. 27408789 PMC4925448

[jnc15516-bib-0054] Di, C. P. , Mansouri, E. , Tong, J. , Wilson, A. A. , Houle, S. , Boileau, I. , Duvauchelle, T. , Robert, P. , Schwartz, J. C. , & Le, F. B. (2019). Occupancy of dopamine D2 and D3 receptors by a novel D3 partial agonist BP1.4979: a [^11^C]‐(+)‐PHNO PET study in humans. Neuropsychopharmacology, 44, 1284–1290. 10.1038/s41386-018-0285-4. 30659274 PMC6785153

[jnc15516-bib-0055] Dias, V. , Junn, E. , & Mouradian, M. M. (2013). The role of oxidative stress in Parkinson’s disease. Journal of Parkinson's Disease, 3, 461–491. 10.3233/JPD-130230 PMC4135313 24252804

[jnc15516-bib-0056] Dickson, D. W. , Braak, H. , Duda, J. E. , Duyckaerts, C. , Gasser, T. , Halliday, G. M. , Hardy, J. , Leverenz, J. B. , Del Tredici, K. , Wszolek, Z. K. , & Litvan, I. (2009). Neuropathological assessment of Parkinson’s disease: Refining the diagnostic criteria. The Lancet Neurology, 8, 1150–1157. 10.1016/S1474-4422(09)70238-8. 19909913

[jnc15516-bib-0057] Dimitrova‐Shumkovska, J. , Krstanoski, L. , & Veenman, L. (2020). Diagnostic and therapeutic potential of TSPO studies regarding neurodegenerative diseases, psychiatric disorders, alcohol use disorders, traumatic brain injury, and stroke: An update. Cells, 9, 870. 10.3390/cells9040870. PMC7226777 32252470

[jnc15516-bib-0058] Ding, Y.‐S. , Fowler, J. S. , Volkow, N. D. , Dewey, S. L. , Wang, G.‐J. , Logan, J. , Gatley, S. J. , & Pappas, N. (1997). Chiral drugs: comparison of the pharmacokinetics of [^11^C]d‐threo and l‐threo ‐methylphenidate in the human and baboon brain. Psychopharmacology (Berl), 131, 71–78. 10.1007/s002130050267. 9181638

[jnc15516-bib-0059] Doder, M. A. , Rabiner, E. J. , Turjanski, N. J. , Lees, A. J. , & Brooks, D. J. (2003). Tremor in Parkinson’s disease and serotonergic dysfunction: An ^11^C‐WAY100635 PET study. Neurology, 60, 601–605. 10.1212/01.WNL.0000031424.51127.2B. 12601099

[jnc15516-bib-0060] Driver, J. A. , Logroscino, G. , Gaziano, J. M. , & Kurth, T. (2009). Incidence and remaining lifetime risk of Parkinson disease in advanced age. Neurology, 72, 432–438. 10.1212/01.wnl.0000341769.50075.bb. 19188574 PMC2676726

[jnc15516-bib-0061] Eberling, J. L. , Dave, K. D. , & Frasier, M. A. (2013). α‐synuclein imaging: A critical need for parkinson’s disease research. Journal of Parkinson's Disease, 3, 565–567. 10.3233/JPD-130247 24192754

[jnc15516-bib-0062] Eckert, T. , Laere, K. , Van, T. C. , Lewis, D. E. , Edwards, C. , Santens, P. , & Eidelberg, D. (2007). Quantification of Parkinson’s disease‐related network expression with ECD SPECT. European Journal of Nuclear Medicine and Molecular Imaging, 34, 496–501. 17096095 10.1007/s00259-006-0261-9

[jnc15516-bib-0063] Eckert, T. , Tang, C. , Ma, Y. , Brown, N. , Lin, T. , Frucht, S. , Feigin, A. , & Eidelberg, D. (2008). Abnormal metabolic networks in atypical parkinsonism. Movement Disorders, 23, 727–733. 10.1002/mds.21933. 18186116

[jnc15516-bib-0064] Edison, P. , Rowe, C. C. , Rinne, J. O. , Ng, S. , Ahmed, I. , Kemppainen, N. , Villemagne, V. L. , O'Keefe, G. , Nagren, K. , Chaudhury, K. R. , Masters, C. L. , & Brooks, D. J. (2008). Amyloid load in Parkinson's disease dementia and Lewy body dementia measured with [^11^C]PIB positron emission tomography. Journal of Neurology, Neurosurgery & Psychiatry, 79, 1331–1338. 10.1136/jnnp.2007.127878. 18653550

[jnc15516-bib-0065] Eglen, R. M. (2012). Overview of muscarinic receptor subtypes, in muscarinic receptors. Handbook of experimental pharmacology, Vol. 208 (pp. 3–28). Springer. 10.1007/978-3-642-23274-9_1 22222692

[jnc15516-bib-0066] Elsinga, P. H. , Hatano, K. , & Ishiwata, K. (2006). PET tracers for imaging of the dopaminergic system. Current Medicinal Chemistry, 13, 2139–2153. 16918344 10.2174/092986706777935258

[jnc15516-bib-0067] EMEA/H/C/000266 European public assessment report (EPAR) for DaTSCAN https://www.ema.europa.eu/en/medicines/human/EPAR/datscan.

[jnc15516-bib-0068] Esposito, G. , Ana, C. F. , & Verstreken, P. (2012). Synaptic vesicle trafficking and Parkinson’s disease. Developmental Neurobiology, 72, 134–144. 21563316 10.1002/dneu.20916

[jnc15516-bib-0069] Estrada, A. A. , & Sweeney, Z. K. (2015). Chemical biology of leucine‐rich repeat kinase 2 (LRRK2) inhibitors. Journal of Medicinal Chemistry, 58, 6733–6746. 25915084 10.1021/acs.jmedchem.5b00261

[jnc15516-bib-0070] Ettrup, A. , Svarer, C. , McMahon, B. , da Cunha‐Bang, S. , Lehel, S. , Møller, K. , Dyssegaard, A. , Ganz, M. , Beliveau, V. , Jørgensen, L. M. , Gillings, N. , & Knudsen, G. M. (2016). Serotonin 2A receptor agonist binding in the human brain with [^11^C]Cimbi‐36: Test‐retest reproducibility and head‐to‐head comparison with the antagonist [^18^F]altanserin. NeuroImage, 130, 167–174. 10.1016/j.neuroimage.2016.02.001. 26876490

[jnc15516-bib-0071] Fakhree, M. A. A. , Nolten, I. S. , Blum, C. , & Claessens, M. M. A. E. (2018). Different conformational subensembles of the intrinsically disordered protein α‐synuclein in cells. The Journal of Physical Chemistry Letters, 9, 1249–1253. 29474083 10.1021/acs.jpclett.8b00092 PMC5857923

[jnc15516-bib-0072] Ferraz, H. B. (2014). Dopamine transporter imaging using ^99m^Tc‐TRODAT‐1 SPECT in Parkinson’s disease. Medical Science Monitor, 20, 1413–1418. 25109468 10.12659/MSM.890522 PMC4138066

[jnc15516-bib-0073] Ferreira, J. J. , Katzenschlager, R. , Bloem, B. R. , Bonuccelli, U. , Burn, D. , Deuschl, G. , Dietrichs, E. , Fabbrini, G. , Friedman, A. , Kanovsky, P. , Kostic, V. , Nieuwboer, A. , Odin, P. , Poewe, W. , Rascol, O. , Sampaio, C. , Schüpbach, M. , Tolosa, E. , Trenkwalder, C. , … Oertel, W. H. (2013). Summary of the recommendations of the EFNS/MDS‐ES review on therapeutic management of Parkinson's disease. European Journal of Neurology, 20, 5–15. 10.1111/j.1468-1331.2012.03866.x. 23279439

[jnc15516-bib-0074] Finnema, S. J. , Nabulsi, N. B. , Eid, T. , Detyniecki, K. , Lin, S.‐F. , Chen, M‐K. , Dhaher, R. , Matuskey, D. , Baum, E. , Holden, D. , Spencer, D. D. , Mercier, J. , Hannestad, J. , Huang, Y. , & Carson, R. E. (2016). Imaging synaptic density in the living human brain. Science Translational Medicine, 8, 348ra96. 10.1126/scitranslmed.aaf6667. 27440727

[jnc15516-bib-0075] Fischman, A. J. , Bonab, A. A. , Babich, J. W. , Palmer, E. P. , Alpert, N. M. , Elmaleh, D. R. , Callahan, R. J. , Barrow, S. A. , Graham, W. , Meltzer, P. C. , Hanson, R. N. , & Madras, B. K. (1998). Rapid detection of Parkinson’s disease by SPECT with altropane: A selective ligand for dopamine transporters. Synapse (New York, N. Y.), 29, 128–141. 10.1002/(SICI)1098-2396(199806)29:2<128:AID-SYN4>3.0.CO;2-9. 9593103

[jnc15516-bib-0076] Fowler, J. S. , Volkow, N. D. , Wolf, A. P. , Dewey, S. L. , Schlyer, D. J. , MacGregor, R. R. , Hitzemann, R. , Logan, J. , Bendriem, B. , Gatley, S. J. , & Christman, D. (1989). Mapping cocaine binding sites in human and baboon brain in vivo. Synapse (New York, N. Y.), 4, 371–377. 10.1002/syn.890040412. 2557686

[jnc15516-bib-0077] Frankle, W. G. , Huang, Y. , Hwang, D.‐R. , Talbot, P. S. , Slifstein, M. , Heertum, R. , Van, A.‐D. , & Laruelle, M. (2004). Comparative evaluation of serotonin transporter radioligands ^11^C‐DASB and ^11^C‐McN5652 in healthy humans. Journal of Nuclear Medicine, 45, 682–694. 15073266

[jnc15516-bib-0078] Fuchigami, T. , Nakayama, M. , & Magata, Y. (2021). Development of PET and SPECT Radioligands for In Vivo Imaging of NMDA Receptors, in PET and SPECT of Neurobiological Systems, pp. 661–711. Springer, Cham.

[jnc15516-bib-0079] Fujita, M. , Ichise, M. , Zoghbi, S. S. , Liow, J.‐S. , Ghose, S. , Vines, D. C. , Sangare, J. , Lu, J.‐Q. , Cropley, V. L. , Iida, H. , Kim, K. M. , Cohen, R. M. , Bara‐Jimenez, W. , Ravina, B. , & Innis, R. B. (2006). Widespread decrease of nicotinic acetylcholine receptors in Parkinson's disease. Annals of Neurology, 59, 174–177. 10.1002/ana.20688. 16374823 PMC1351078

[jnc15516-bib-0080] Gallagher, C. L. , Christian, B. T. , Holden, J. E. , Dejesus, O. T. , Nickles, R. J. , Buyan‐Dent, L. , Bendlin, B. B. , Harding, S. J. , Stone, C. K. , Mueller, B. , & Johnson, S. C. (2011). A within‐subject comparison of 6‐[^18^F]fluoro‐m‐tyrosine and 6‐[^18^F]fluoro‐L ‐dopa in Parkinson's disease. Movement Disorders, 26, 2032–2038. 10.1002/mds.23778. 21638324 PMC3278160

[jnc15516-bib-0081] Gallagher, C. L. , Oakes, T. R. , Johnson, S. C. , Chung, M. K. , Holden, J. E. , Bendlin, B. B. , McLaren, D. G. , Xu, G. , Nickles, R. J. , Pyzalski, R. , DeJesus, O. , & Brown, W. D. (2011). Rate of 6‐[^18^F]fluorodopa uptake decline in striatal subregions in Parkinson’s disease. Movement Disorders, 26, 614–620. 10.1002/mds.23503. 21449008 PMC3080432

[jnc15516-bib-0082] Garnett, E. S. , Firnau, G. , & Nahmias, C. (1983). Dopamine visualized in the basal ganglia of living man. Nature, 305, 137–138. 10.1038/305137a0. 6604227

[jnc15516-bib-0083] Geday, J. , Østergaard, K. , Johnsen, E. , & Gjedde, A. (2009). STN‐Stimulation in Parkinson’s disease restores striatal inhibition of thalamocortical projection. Human Brain Mapping, 30, 112–121. 10.1002/hbm.20486. 18041743 PMC6870788

[jnc15516-bib-0084] Gee, A. D. , Herth, M. M. , James, M. L. , Korde, A. , Scott, P. J. H. , & Vasdev, N. (2020). Radionuclide imaging for neuroscience: Current opinion and future directions. Molecular Imaging, 19, 1–9. 10.1177/1536012120936397 PMC7493278 32907484

[jnc15516-bib-0085] Gerhard, A. , Pavese, N. , Hotton, G. , Turkheimer, F. , Es, M. , Hammers, A. , Eggert, K. , Oertel, W. , Banati, R. B. , & Brooks, D. J. (2006). In vivo imaging of microglial activation with [^11^C](R)‐PK11195 PET in idiopathic Parkinson’s disease. Neurobiology of Diseases, 21, 404–412. 10.1016/j.nbd.2005.08.002 16182554

[jnc15516-bib-0086] Ghadery, C. , Koshimori, Y. , Coakeley, S. , Harris, M. , Rusjan, P. , Kim, J. , Houle, S. , & Strafella, A. P. (2017). Microglial activation in Parkinson’s disease using [^18^F]FEPPA. Journal of Neuroinflammation, 14, 1–8. 10.1186/s12974-016-0778-1. 28086916 PMC5234135

[jnc15516-bib-0087] Gilman, S. , Koeppe, R. A. , Nan, B. , Wang, C. N. , Wang, X. , Junck, L. , Chervin, R. D. , Consens, F. , & Bhaumik, A. (2010). Cerebral cortical and subcortical cholinergic deficits in parkinsonian syndromes. Neurology, 74, 1416–1423. 10.1212/WNL.0b013e3181dc1a55. 20439843 PMC2871002

[jnc15516-bib-0088] Gjerløff, T. , Fedorova, T. , Knudsen, K. , Munk, O. L. , Nahimi, A. , Jacobsen, S. , Danielsen, E. H. , Terkelsen, A. J. , Hansen, J. , Pavese, N. , Brooks, D. J. , & Borghammer, P. (2015). Imaging acetylcholinesterase density in peripheral organs in Parkinson’s disease with ^11^C‐donepezil PET. Brain, 138, 653–663. 10.1093/brain/awu369. 25539902 PMC4408425

[jnc15516-bib-0089] Glover, G. H. (2011). Overview of functional magnetic resonance imaging. Neurosurgery Clinics of North America, 22, 133–139. 21435566 10.1016/j.nec.2010.11.001 PMC3073717

[jnc15516-bib-0090] Goedert, M. , Spillantini, M. G. , Tredici, K. , & Del, B. H. (2013). 100 years of Lewy pathology. Nature Reviews Neurology, 9, 13–24. 10.1038/nrneurol.2012.242 23183883

[jnc15516-bib-0091] Goetz, C. G. , Poewe, W. , Rascol, O. , & Sampaio, C. (2005). Evidence‐based medical review update: Pharmacological and surgical treatments of Parkinson’s disease: 2001 to 2004. Movement Disorders, 20, 523–539. 10.1002/mds.20464. 15818599

[jnc15516-bib-0092] Goldstein, D. S. (2000). Cardiac sympathetic denervation in Parkinson disease. Annals of Internal Medicine, 133, 338–347. 10979878 10.7326/0003-4819-133-5-200009050-00009

[jnc15516-bib-0093] Grachev, I. , Doder, M. , Brooks, D. , & Hinz, R. (2014). Quantitative in vivo imaging of adenosine A2A receptors in the human brain using ^11^C‐SCH442416 PET: A pilot study. Journal of Diagnostic Imaging in Therapy, 1, 1–19. 10.17229/jdit.2014-0620-001.

[jnc15516-bib-0094] Guilarte, T. R. (2019). TSPO in diverse CNS pathologies and psychiatric disease: A critical review and a way forward. Pharmacology & Therapeutics, 194, 44–58. 10.1016/j.pharmthera.2018.09.003. 30189290 PMC6348013

[jnc15516-bib-0095] Gustavsson, A. , Svensson, M. , Jacobi, F. , Allgulander, C. , Alonso, J. , Beghi, E. , Dodel, R. , Ekman, M. , Faravelli, C. , Fratiglioni, L. , Gannon, B. , Jones, D. H. , Jennum, P. , Jordanova, A. , Jönsson, L. , Karampampa, K. , Knapp, M. , Kobelt, G. , Kurth, T. , … Olesen, J. (2011). Cost of disorders of the brain in Europe 2010. European Neuropsychopharmacology, 21, 718–779. 10.1016/j.euroneuro.2011.08.008. 21924589

[jnc15516-bib-0096] Guttman, M. , Burkholder, J. , Kish, S. J. , Hussey, D. , Wilson, A. , DaSilva, J. , & Houle, S. (1997). [^11^C]RTI‐32 PET studies of the dopamine transporter in early dopa‐naive Parkinson’s disease: Implications for the symptomatic threshold. Neurology, 48, 1578–1583. 9191769 10.1212/wnl.48.6.1578

[jnc15516-bib-0097] Haining, R. L. , & Achat‐Mendes, C. (2017). Neuromelanin, one of the most overlooked molecules in modern medicine, is not a spectator. Neural Regeneration Research, 12, 372–375. 28469642 10.4103/1673-5374.202928 PMC5399705

[jnc15516-bib-0098] Hansen, A. K. , Knudsen, K. , Lillethorup, T. P. , Landau, A. M. , Parbo, P. , Fedorova, T. , Audrain, H. , Bender, D. , Østergaard, K. , Brooks, D. J. , & Borghammer, P. (2016). In vivo imaging of neuromelanin in Parkinson’s disease using ^18^F‐AV‐1451 PET. Brain, 139, 2039–2049. 10.1093/brain/aww098. 27190023

[jnc15516-bib-0099] Harrison, I. F. , & Dexter, D. T. (2013). Epigenetic targeting of histone deacetylase: Therapeutic potential in Parkinson’s disease? Pharmacology & Therapeutics, 140, 34–52. 10.1016/j.pharmthera.2013.05.010. 23711791

[jnc15516-bib-0100] Hauser, D. N. , & Hastings, T. G. (2013). Mitochondrial dysfunction and oxidative stress in Parkinson’s disease and monogenic parkinsonism. Neurobiology of Diseases, 51, 35–42. 10.1016/j.nbd.2012.10.011 PMC3565564 23064436

[jnc15516-bib-0101] Hegarty, S. V. , Sullivan, A. M. , & O’Keeffe, G. W. (2016). The Epigenome as a therapeutic target for Parkinson’s disease. Neural Regeneration Research, 11, 1735–1738. 28123403 10.4103/1673-5374.194803 PMC5204215

[jnc15516-bib-0102] Helmich, R. C. , Derikx, L. C. , Bakker, M. , Scheeringa, R. , Bloem, B. R. , & Toni, I. (2010). Spatial remapping of Cortico‐striatal connectivity in Parkinson’s disease. Cerebral Cortex, 20, 1175–1186. 10.1093/cercor/bhp178. 19710357

[jnc15516-bib-0103] Helmich, R. C. , Vaillancourt, D. E. , & Brooks, D. J. (2018). The future of brain imaging in Parkinson’s disease. Journal of Parkinson's Disease, 8, S47–S51. 10.3233/JPD-181482. PMC6311365 30584163

[jnc15516-bib-0104] Herrington, T. M. , Cheng, J. J. , & Eskandar, E. N. (2016). Mechanisms of deep brain stimulation. Journal of Neurophysiology, 115, 19–38. 10.1152/jn.00281.2015. 26510756 PMC4760496

[jnc15516-bib-0105] Herth, M. M. , & Knudsen, G. M. (2015). Current radiosynthesis strategies for 5‐HT 2A receptor PET tracers. Journal of Labelled Compounds and Radiopharmaceuticals, 58, 265–273. 25997728 10.1002/jlcr.3288

[jnc15516-bib-0106] Herz, D. M. , Eickhoff, S. B. , Løkkegaard, A. , & Siebner, H. R. (2014). Functional neuroimaging of motor control in Parkinson’s disease: A meta‐analysis. Human Brain Mapping, 35, 3227–3237. 10.1002/hbm.22397. 24123553 PMC6869014

[jnc15516-bib-0107] Heurling, K. , Ashton, N. J. , Leuzy, A. , Zimmer, E. R. , Blennow, K. , Zetterberg, H. , Eriksson, J. , Lubberink, M. , & Schöll, M. (2019). Synaptic vesicle protein 2A as a potential biomarker in synaptopathies. Molecular and Cellular Neurosciences, 97, 34–42. 30796959 10.1016/j.mcn.2019.02.001

[jnc15516-bib-0108] Hilker, R. , Thomas, A. V. , Klein, J. C. , Weisenbach, S. , Kalbe, E. , Burghaus, L. , Jacobs, A. H. , Herholz, K. , & Heiss, W. D. (2005). Dementia in Parkinson disease: Functional imaging of cholinergic and dopaminergic pathways. Neurology, 65, 1716–1722. 10.1212/01.wnl.0000191154.78131.f6. 16344512

[jnc15516-bib-0109] Hirano, S. , Asanuma, K. , Ma, Y. , Tang, C. , Feigin, A. , Dhawan, V. , Carbon, M. , & Eidelberg, D. (2008). Dissociation of metabolic and neurovascular responses to levodopa in the treatment of Parkinson’s disease. Journal of Neuroscience, 28, 4201–4209. 10.1523/JNEUROSCI.0582-08.2008. 18417699 PMC2577921

[jnc15516-bib-0110] Hiraoka, K. , Okamura, N. , Funaki, Y. , Hayashi, A. , Tashiro, M. , Hisanaga, K. , Fujii, T. , Takeda, A. , Yanai, K. , Iwata, R. , & Mori, E. (2012). Cholinergic deficit and response to donepezil therapy in Parkinson's disease with dementia. European Neurology, 68, 137–143. 10.1159/000338774. 22832236

[jnc15516-bib-0111] Hirsch, E. C. , Breidert, T. , Rousselet, E. , Hunot, S. , Hartmann, A. , & Michel, P. P. (2003). The role of glial reaction and inflammation in Parkinson’s disease. Annals of the New York Academy of Sciences, 991, 214–228. 12846989 10.1111/j.1749-6632.2003.tb07478.x

[jnc15516-bib-0112] Hirst, J. (2013). Mitochondrial complex I. Annual Review of Biochemistry, 82, 551–575. 10.1146/annurev-biochem-070511-103700 23527692

[jnc15516-bib-0113] Horsager, J. , Andersen, K. B. , Knudsen, K. , Skjærbæk, C. , Fedorova, T. D. , Okkels, N. , Schaeffer, E. , Bonkat, S. K. , Geday, J. , Otto, M. , Sommerauer, M. , Danielsen, E. H. , Bech, E. , Kraft, J. , Munk, O. L. , Hansen, S. D. , Pavese, N. , Göder, R. , Brooks, D. J. , … Borghammer, P. (2020). Brain‐first versus body‐first Parkinson’s disease: a multimodal imaging case‐control study. Brain, 143, 3077–3088. 10.1093/brain/awaa238. 32830221

[jnc15516-bib-0114] Hossein‐Tehrani, M. R. , Ghaedian, T. , Hooshmandi, E. , Kalhor, L. , Foroughi, A. A. , & Ostovan, V. R. (2020). Brain TRODAT‐SPECT versus MRI morphometry in distinguishing early mild Parkinson’s disease from other extrapyramidal syndromes. Journal of Neuroimaging, 30, 683–689. 10.1111/jon.12740. 32557946

[jnc15516-bib-0115] Hsieh, C.‐J. , Ferrie, J. J. , Xu, K. , Lee, I. , Graham, T. J. A. , Tu, Z. , Yu, J. , Dhavale, D. , Kotzbauer, P. , Petersson, E. J. , & Mach, R. H. (2018). Alpha synuclein fibrils contain multiple binding sites for small molecules. ACS Chemical Neuroscience, 9, 2521–2527. 10.1021/acschemneuro.8b00177. 29750499 PMC6736640

[jnc15516-bib-0116] Hsieh, C.‐J. , Xu, K. , Lee, I. , Graham, T. J. A. , Tu, Z. , Dhavale, D. , Kotzbauer, P. , & Mach, R. H. (2018). Chalcones and five‐membered heterocyclic isosteres bind to alpha synuclein fibrils in vitro. ACS Omega, 3, 4486–4493. 10.1021/acsomega.7b01897. 30221226 PMC6130786

[jnc15516-bib-0117] Huber, T. , Dietrich, D. , & Emrich, H. (1999). Possible use of amantadine in depression. Pharmacopsychiatry, 32, 47–55. 10.1055/s-2007-979191. 10333162

[jnc15516-bib-0118] Hwang, W. J. , Yao, W. J. , Wey, S. P. , & Ting, G. (2004). Reproducibility of ^99m^Tc‐TRODAT‐1 SPECT measurement of dopamine transporters in Parkinson’s disease. Journal of Nuclear Medicine, 45, 207–213. 14960637

[jnc15516-bib-0119] Iadanza, M. G. , Jackson, M. P. , Hewitt, E. W. , Ranson, N. A. , & Radford, S. E. (2018). A new era for understanding amyloid structures and disease. Nature Reviews Molecular Cell Biology, 19, 755–773. 30237470 10.1038/s41580-018-0060-8 PMC7617691

[jnc15516-bib-0120] Ibrahim, N. , Kusmirek, J. , Struck, A. F. , Floberg, J. M. , Perlman, S. B. , Gallagher, C. , & Hall, L. T. (2016). The sensitivity and specificity of F‐DOPA PET in a movement disorder clinic. American Journal of Nuclear Medicine and Molecular Imaging, 6, 102–109. 27069770 PMC4749509

[jnc15516-bib-0121] Ignacio, A. J. , De, A. M. , Alcazar, J. , Celen, S. , & Bormans, G. (2012). Recent advances in positron emission tomography (PET) radiotracers for imaging phosphodiesterases. Current Topics in Medicinal Chemistry, 12, 1224–1236. 22571785 10.2174/156802612800672853

[jnc15516-bib-0122] Ikeda, K. , Ebina, J. , Kawabe, K. , & Iwasaki, Y. (2019). Dopamine transporter imaging in Parkinson disease: Progressive changes and therapeutic modification after anti‐parkinsonian medications. Internal Medicine, 58, 1665–1672. 30799370 10.2169/internalmedicine.2489-18 PMC6630131

[jnc15516-bib-0123] Irie, T. , Fukushi, K. , Namba, H. , Iyo, M. , Tamagami, H. , Nagatsuka, S. , & Ikota, N. (1996). Brain acetylcholinesterase activity: Validation of a PET tracer in a rat model of Alzheimer’s disease. Journal of Nuclear Medicine, 37, 649–655. 8691261

[jnc15516-bib-0124] Ishibashi, K. , Miura, Y. , Wagatsuma, K. , Toyohara, J. , Ishiwata, K. , & Ishii, K. (2018). Occupancy of adenosine A2A receptors by istradefylline in patients with Parkinson’s disease using ^11^C‐preladenant PET. Neuropharmacology, 143, 106–112. 10.1016/j.neuropharm.2018.09.036. 30253174

[jnc15516-bib-0125] Ishikawa, M. , Sakata, M. , Ishii, K. , Kimura, Y. , Oda, K. , Toyohara, J. , Wu, J. , Ishiwata, K. , Iyo, M. , & Hashimoto, K. (2009). High occupancy of σ1 receptors in the human brain after single oral administration of donepezil: A positron emission tomography study using [^11^C]SA4503. International Journal of Neuropsychopharmacology, 12, 1127–1131. 10.1017/S1461145709990204. 19573265

[jnc15516-bib-0126] Jakobson Mo, S. , Axelsson, J. , Jonasson, L. , Larsson, A. , Ögren, M. J. , Ögren, M. , Varrone, A. , Eriksson, L. , Bäckström, D. , af Bjerkén, S. , Linder, J. , & Riklund, K. (2018). Dopamine transporter imaging with [^18^F]FE‐PE2I PET and [^123^I]FP‐CIT SPECT—a clinical comparison. EJNMMI Research, 8, 100. 10.1186/s13550-018-0450-0. 30443684 PMC6238014

[jnc15516-bib-0127] Jasanoff, A. (2007). MRI contrast agents for functional molecular imaging of brain activity. Current Opinion in Neurobiology, 17, 593–600. 18093824 10.1016/j.conb.2007.11.002 PMC2883914

[jnc15516-bib-0128] Jie, C. V. M. L. , Treyer, V. , Schibli, R. , & Mu, L. (2021). Tauvid^TM^: The first FDA‐approved pet tracer for imaging tau pathology in Alzheimer’s disease. Pharmaceuticals, 14, 1–12. 10.3390/ph14020110 PMC7911942 33573211

[jnc15516-bib-0129] Jovalekic, A. , Koglin, N. , Mueller, A. , & Stephens, A. W. (2017). New protein deposition tracers in the pipeline. EJNMMI Radiopharmacy and Chemistry, 1, 11. 10.1186/s41181-016-0015-3. 29564387 PMC5843813

[jnc15516-bib-0130] Joyce, J. N. , & Millan, M. J. (2007). Dopamine D_3_ receptor agonists for protection and repair in Parkinson’s disease. Current Opinion in Pharmacology, 7, 100–105. 17174156 10.1016/j.coph.2006.11.004

[jnc15516-bib-0131] Jung, L. H. , Weng, Y.‐H. , Wen, M.‐C. , Hsiao, I.‐T. , & Lin, K.‐J. (2018). Quantitative study of ^18^F‐(+)DTBZ image: Comparison of PET template‐based and MRI based image analysis. Scientific Reports, 8, 16027. 10.1038/s41598-018-34388-6. 30375444 PMC6207708

[jnc15516-bib-0132] Juri, C. , Kramer, V. , Riss, P. J. , Soza‐Ried, C. , Haeger, A. , Pruzzo, R. , Rösch, F. , Amaral, H. , & Chana‐Cuevas, P. (2021). [^18^F]PR04.MZ PET/CT imaging for evaluation of nigrostriatal neuron integrity in patients with Parkinson disease. Clinical Nuclear Medicine, 46, 119–124. 33323728 10.1097/RLU.0000000000003430 PMC7774816

[jnc15516-bib-0133] Kaasinen, V. , Aalto, S. , Någren, K. , Hietala, J. , Sonninen, P. , & Rinne, J. O. (2003). Extrastriatal dopamine D_2_ receptors in Parkinson’s disease: A longitudinal study. Journal of Neural Transmission, 110, 591–601. 10.1007/s00702-003-0816-x. 12768355

[jnc15516-bib-0134] Kalia, L. V. , Kalia, S. K. , McLean, P. J. , Lozano, A. M. , & Lang, A. E. (2013). α‐synuclein oligomers and clinical implications for Parkinson disease. Annals of Neurology, 73, 155–169. 23225525 10.1002/ana.23746 PMC3608838

[jnc15516-bib-0135] Kameyama, M. , Murakami, K. , & Jinzaki, M. (2016). Comparison of [^15^O] H_2_O positron emission tomography and functional magnetic resonance imaging in activation studies. World Journal of Nuclear Medicine, 15, 3–6. 26912971 10.4103/1450-1147.172139 PMC4729011

[jnc15516-bib-0136] Kepe, V. , Bordelon, Y. , Boxer, A. , Huang, S.‐C. , Liu, J. , Thiede, F. C. , Mazziotta, J. C. , Mendez, M. F. , Donoghue, N. , Small, G. W. , & Barrio, J. R. (2013). PET imaging of neuropathology in tauopathies: Progressive supranuclear palsy. Journal of Alzheimer's Disease, 36, 145–153. 10.3233/JAD-130032. PMC3674205 23579330

[jnc15516-bib-0137] Khalil, M. M. , Tremoleda, J. L. , Bayomy, T. B. , & Gsell, W. (2011). Molecular SPECT imaging: An overview. International Journal of Molecular Imaging, 2011, 1–15. 10.1155/2011/796025 PMC3094893 21603240

[jnc15516-bib-0138] Kilbourn, M. R. , Frey, K. A. , Borght, T. , & Vander, S. P. S. (1996). Effects of dopaminergic drug treatments on in vivo radioligand binding to brain vesicular monoamine transporters. Nuclear Medicine and Biology, 23, 467–471. 8832701 10.1016/0969-8051(96)00023-6

[jnc15516-bib-0139] Kim, W. S. , Kagedal, K. , & Halliday, G. M. (2014). Alpha‐synuclein biology in Lewy body diseases. Alzheimer's Research & Therapy, 6, 1–9. 10.1186/s13195-014-0073-2 PMC4288216 25580161

[jnc15516-bib-0140] Kohl, Z. , & Winkler, J. (2020). Serotonin in Parkinson’s disease. Handbook of Behavioral Neuroscience, 31, 969–979.

[jnc15516-bib-0141] Korat, Š. , Bidesi, N. S. R. , Bonanno, F. , Di Nanni, A. , Hoàng, A. N. N. , Herfert, K. , Maurer, A. , Battisti, U. M. , Bowden, G. D. , Thonon, D. , Vugts, D. , Windhorst, A. D. , & Herth, M. M. (2021). Alpha‐synuclein PET tracer development—An overview about current efforts. Pharmaceuticals, 14, 847. 10.3390/ph14090847. 34577548 PMC8466155

[jnc15516-bib-0142] Kotzbauer, P. T. , Tu, Z. , & Mach, R. H. (2017). Current status of the development of PET radiotracers for imaging alpha synuclein aggregates in Lewy bodies and Lewy neurites. Clinical and Translational Imaging, 5, 3–14.

[jnc15516-bib-0143] Kramer, V. , Dyssegaard, A. , Flores, J. , Soza‐Ried, C. , Rösch, F. , Knudsen, G. M. , Amaral, H. , & Herth, M. M. (2020). Characterization of the serotonin 2A receptor selective PET tracer (R)‐[^18^F]MH.MZ in the human brain. European Journal of Nuclear Medicine and Molecular Imaging, 47, 355–365. 10.1007/s00259-019-04527-w. 31606832

[jnc15516-bib-0144] Kramer, V. , Juri, C. , Riss, P. J. , Pruzzo, R. , Soza‐Ried, C. , Flores, J. , Hurtado, A. , Rösch, F. , Chana‐Cuevas, P. , & Amaral, H. (2020). Pharmacokinetic evaluation of [^18^F]PR04.MZ for PET/CT imaging and quantification of dopamine transporters in the human brain. European Journal of Nuclear Medicine and Molecular Imaging, 47, 1927–1937. 10.1007/s00259-019-04594-z. 31788709

[jnc15516-bib-0145] Kristensen, J. L. , & Herth, M. M. (2017). In vivo imaging in drug discovery. In K. Stromgaard , P. Krogsgaard‐Larsen , & U. Madsen (Eds.), Textbook of drug design and discovery, 5th ed. (pp. 119–135). CRC Press.

[jnc15516-bib-0146] Kuebler, L. , Buss, S. , Leonov, A. , Ryazanov, S. , Schmidt, F. , Maurer, A. , Weckbecker, D. , Landau, A. M. , Lillethorup, T. P. , Bleher, D. , Saw, R. S. , Pichler, B. J. , Griesinger, C. , Giese, A. , & Herfert, K. (2021). [^11^C]MODAG‐001—towards a PET tracer targeting α‐synuclein aggregates. European Journal of Nuclear Medicine and Molecular Imaging, 48, 1759–1772. 10.1007/s00259-020-05133-x. 33369690 PMC8113290

[jnc15516-bib-0147] Kuhl, D. E. , Minoshima, S. , Fessler, J. A. , Ficaro, E. P. , Wieland, D. M. , Koeppe, R. A. , Frey, K. A. , & Foster, N. L. (1996). In vivo mapping of cholinergic terminals in normal aging, Alzheimer’s disease, and Parkinson’s disease. Annals of Neurology, 40, 399–410. 8797529 10.1002/ana.410400309

[jnc15516-bib-0148] Kuikka, J. T. , Baulieu, J. L. , Hiltunen, J. , Halldin, C. , Bergström, K. A. , Farde, L. , Emond, P. , Chalon, S. , Yu, M. , Nikula, T. , Laitinen, T. , Karhu, J. , Tupala, E. , Hallikainen, T. , Kolehmainen, V. , Mauclaire, L. , Maziere, B. , Tiihonen, J. , & Guilloteau, D. (1998). Pharmacokinetics and dosimetry of iodine‐123 labelled PE2I in humans, a radioligand for dopamine transporter imaging. European Journal of Nuclear Medicine and Molecular Imaging, 25, 531–534. 10.1007/s002590050254. 9575250

[jnc15516-bib-0149] Kung, H. F. , Kim, H.‐J. , Kung, M.‐P. , Meegalla, S. K. , Plössl, K. , & Lee, H.‐K. (1996). Imaging of dopamine transporters in humans with technetium‐99m TRODAT 1. European Journal of Nuclear Medicine, 23, 1527–1530. 8854853 10.1007/BF01254479

[jnc15516-bib-0150] Kuriakose, R. , & Stoessl, A. J. (2010). Imaging the nigrostriatal system to monitor disease progression and treatment‐induced complications, in Recent Adv. Parkinson Disease, 184, 177–192. 10.1016/S0079-6123(10)84009-9 20887875

[jnc15516-bib-0151] L’Estrade, E. T. , Hansen, H. D. , Erlandsson, M. , Ohlsson, T. G. , Knudsen, G. M. , & Herth, M. M. (2018). Classics in neuroimaging: The serotonergic 2A receptor system‐from discovery to modern molecular imaging. ACS Chemical Neuroscience, 9, 1226–1229. 29763291 10.1021/acschemneuro.8b00176

[jnc15516-bib-0152] La, F. C. , Pöpperl, G. , Levin, J. , Wängler, B. , Böning, G. , Uebleis, C. , Cumming, P. , Bartenstein, P. , Bötzel, K. , & Tatsch, K. (2010). The value of the dopamine D2/3 receptor ligand ^18^F‐desmethoxyfallypride for the differentiation of idiopathic and nonidiopathic Parkinsonian syndromes. Journal of Nuclear Medicine, 51, 581–587. 20237026 10.2967/jnumed.109.071811

[jnc15516-bib-0154] Laakso, A. , Bergman, J. , Haaparanta, M. , Vilkman, H. , Solin, O. , & Hietala, J. (1998). [^18^F]CFT [(^18^F)WIN 35,428], a radioligand to study the dopamine transporter with PET: Characterization in human subjects. Synapse (New York, N. Y.), 28, 244–250. 10.1002/(SICI)1098-2396(199803)28:3<244:AID-SYN7>3.0.CO;2-A. 9488509

[jnc15516-bib-0155] Langer, O. , & Halldin, C. (2002). PET and SPET tracers for mapping the cardiac nervous system. European Journal of Nuclear Medicine, 29, 416–434. 12002720 10.1007/s002590100640

[jnc15516-bib-0156] Langston, J. , Ballard, P. , Tetrud, J. , & Irwin, I. (1983). Chronic Parkinsonism in humans due to a product of meperidine‐analog synthesis. Science, 219, 979–980. 10.1126/science.6823561. 6823561

[jnc15516-bib-0157] Laruelle, M. (2000). Imaging synaptic neurotransmission with in vivo binding competition techniques: A critical review. Journal of Cerebral Blood Flow and Metabolism, 20, 423–451. 10724107 10.1097/00004647-200003000-00001

[jnc15516-bib-0158] Laruelle, M. (2012). Measuring dopamine synaptic transmission with molecular imaging and pharmacological challenges: The state of the art. Neuromethods, 71, 163–203.

[jnc15516-bib-0159] Lastres‐Becker, I. , Cebeira, M. , Ceballos, M. L. , & de Zeng, B.‐Y. , Jenner, P. , Ramos, J. A. , & Fernández‐Ruiz, J. J. (2001). Increased cannabinoid CB 1 receptor binding and activation of GTP‐binding proteins in the basal ganglia of patients with Parkinson’s syndrome and of MPTP‐treated marmosets. European Journal of Neuroscience, 14, 1827–1832. 11860478 10.1046/j.0953-816x.2001.01812.x

[jnc15516-bib-0160] Le, F. B. , Wilson, A. A. , Graff, A. , Boileau, I. , & Di, C. P. (2014). Recent methods for measuring dopamine D3 receptor occupancy in vivo: importance for drug development. Frontiers in Pharmacology, 5, 161. 25071579 10.3389/fphar.2014.00161 PMC4090596

[jnc15516-bib-0161] Lee, C. S. , Samii, A. , Sossi, V. , Ruth, T. J. , Schulzer, M. , Holden, J. E. , Wudel, J. , Pal, P. K. , De La Fuente‐Fernandez, R. , Calne, D. B. , & Stoessl, A. J. (2000). In vivo positron emission tomographic evidence for compensatory changes in presynaptic dopaminergic nerve terminals in Parkinson's disease. Annals of Neurology, 47, 493–503. 10.1002/1531-8249(200004)47:4<493::aid-ana13>3.0.co;2-4. 10762161

[jnc15516-bib-0162] Lee, H.‐J. , Baek, S. M. , Ho, D.‐H. , Suk, J.‐E. , Cho, E.‐D. , & Lee, S.‐J. (2011). Dopamine promotes formation and secretion of non‐fibrillar alpha‐synuclein oligomers. Experimental & Molecular Medicine, 43, 216. 21415592 10.3858/emm.2011.43.4.026 PMC3085740

[jnc15516-bib-0163] Lei, P. , Ayton, S. , Finkelstein, D. I. , Adlard, P. A. , Masters, C. L. , & Bush, A. I. (2010). Tau protein: Relevance to Parkinson’s disease. International Journal of Biochemistry & Cell Biology, 42, 1775–1778. 20678581 10.1016/j.biocel.2010.07.016

[jnc15516-bib-0164] Leroy, E. , Boyer, R. , Auburger, G. , Leube, B. , Ulm, G. , Mezey, E. , Harta, G. , Brownstein, M. J. , Jonnalagada, S. , Chernova, T. , Dehejia, A. , Lavedan, C. , Gasser, T. , Steinbach, P. J. , Wilkinson, K. D. , & Polymeropoulos, M. H. (1998). The ubiquitin pathway in Parkinson’s disease. Nature, 395, 451–452. 10.1038/26652. 9774100

[jnc15516-bib-0165] Li, C. T. , Palotti, M. , Holden, J. E. , Oh, J. , Okonkwo, O. , Christian, B. T. , Bendlin, B. B. , Buyan‐Dent, L. , Harding, S. J. , Stone, C. K. , DeJesus, O. T. , Nickles, R. J. , & Gallagher, C. L. (2014). A dual‐tracer study of extrastriatal 6‐[^18^F]fluoro‐m‐tyrosine and 6‐[^18^F]‐fluoro‐l‐dopa Uptake in Parkinson's disease. Synapse, 68, 325–331. 10.1002/syn.21745. 24710997 PMC4284201

[jnc15516-bib-0166] Li, W. , Lao‐Kaim, N. P. , Roussakis, A. A. , Martín‐Bastida, A. , Valle‐Guzman, N. , Paul, G. , Loane, C. , Widner, H. , Politis, M. , Foltynie, T. , Barker, R. A. , & Piccini, P. (2018). ^11^C‐PE2I and ^18^F‐Dopa PET for assessing progression rate in Parkinson's: A longitudinal study. Movement Disorders, 33, 117–127. 10.1002/mds.27183. 29082547

[jnc15516-bib-0167] Lim, E. W. , Aarsland, D. , Ffytche, D. , Taddei, R. N. , van Wamelen, D. J. , Wan, Y.‐M. , Tan, E. K. , & Ray Chaudhuri, K. (2019). Amyloid‐β and Parkinson’s disease. Journal of Neurology, 266, 2605–2619. 10.1007/s00415-018-9100-8. 30377818

[jnc15516-bib-0168] Liu, S.‐Y. , Wile, D. J. , Fu, J. F. , Valerio, J. , Shahinfard, E. , McCormick, S. , Mabrouk, R. , Vafai, N. , McKenzie, J. , Neilson, N. , Perez‐Soriano, A. , Arena, J. E. , Cherkasova, M. , Chan, P. , Zhang, J. , Zabetian, C. P. , Aasly, J. O. , Wszolek, Z. K. , McKeown, M. J. , … Stoessl, A. J. (2018). The effect of LRRK2 mutations on the cholinergic system in manifest and premanifest stages of Parkinson’s disease: A cross‐sectional PET study. The Lancet Neurology, 17, 309–316. 10.1016/S1474-4422(18)30032-2. 29456161 PMC5942215

[jnc15516-bib-0169] Lotharius, J. , & Brundin, P. (2002). Pathogenesis of Parkinson’s disease: Dopamine, vesicles and alpha‐synuclein. Nature Reviews Neuroscience, 3, 932–942. 12461550 10.1038/nrn983

[jnc15516-bib-0170] Lundkvist, C. , Halldin, C. , Ginovart, N. , Swahn, C.‐G. , & Farde, L. (1997). [^18^F]β‐CIT‐FP is superior to [^11^C]β‐CIT‐FP for quantitation of the dopamine transporter. Nuclear Medicine and Biology, 24, 621–627. 9352532 10.1016/s0969-8051(97)00077-2

[jnc15516-bib-0171] Ma, Y. , Huang, C. , Dyke, J. P. , Pan, H. , Alsop, D. , Feigin, A. , & Eidelberg, D. (2010). Parkinson’s disease spatial covariance pattern: Noninvasive quantification with perfusion MRI. Journal of Cerebral Blood Flow and Metabolism, 30, 505–509. 10.1038/jcbfm.2009.256. 20051975 PMC2949137

[jnc15516-bib-0172] Ma, Y. , Peng, S. , Dhawan, V. , & Eidelberg, D. (2011). Cerebral glucose metabolism and blood flow in Parkinson’s disease, in imaging in Parkinson’s disease (pp. 21–31). Oxford University Press.

[jnc15516-bib-0173] Ma, Y. , Tang, C. , Chaly, T. , Greene, P. , Breeze, R. , Fahn, S. , Freed, C. , Dhawan, V. , & Eidelberg, D. (2010). Dopamine cell implantation in Parkinson’s disease: Long‐term clinical and ^18^F‐FDOPA PET outcomes. Journal of Nuclear Medicine, 51, 7–15. 20008998 10.2967/jnumed.109.066811 PMC2946843

[jnc15516-bib-0174] Ma, Y. , Tang, C. , Spetsieris, P. G. , Dhawan, V. , & Eidelberg, D. (2007). Abnormal metabolic network activity in Parkinson’s disease: Test‐retest reproducibility. Journal of Cerebral Blood Flow and Metabolism, 27, 597–605. 10.1038/sj.jcbfm.9600358. 16804550 PMC4455600

[jnc15516-bib-0175] Mach, R. H. , & Luedtke, R. R. (2018). Challenges in the development of dopamine D2‐ and D3‐selective radiotracers for PET imaging studies. Journal of Labelled Compounds and Radiopharmaceuticals, 61, 291–298. 28857231 10.1002/jlcr.3558

[jnc15516-bib-0176] Mankoff, D. A. (2007). A definition of molecular imaging. Journal of Nuclear Medicine, 48, 18–21. 17536102

[jnc15516-bib-0177] Mansur, A. , Rabiner, E. A. , Comley, R. A. , Lewis, Y. , Middleton, L. T. , Huiban, M. , Passchier, J. , Tsukada, H. , & Gunn, R. N. (2020). Characterization of 3 PET tracers for quantification of mitochondrial and synaptic function in healthy human brain: ^18^F‐BCPP‐EF, ^11^C‐SA‐4503, and ^11^C‐UCB‐J. Journal of Nuclear Medicine, 61, 96–103. 31324712 10.2967/jnumed.119.228080

[jnc15516-bib-0178] Mansur, A. , Rabiner, E. A. , Tsukada, H. , Comley, R. A. , Lewis, Y. , Huiban, M. , Passchier, J. , & Gunn, R. N. (2021). Test–retest variability and reference region‐based quantification of ^18^F‐BCPP‐EF for imaging mitochondrial complex I in the human brain. Journal of Cerebral Blood Flow and Metabolism, 41, 771–779. 32501157 10.1177/0271678X20928149 PMC7983506

[jnc15516-bib-0179] Marsili, L. , Rizzo, G. , & Colosimo, C. (2018). Diagnostic criteria for Parkinson’s disease: From James Parkinson to the concept of prodromal disease. Frontiers in Neurology, 9, 1–10. 10.3389/fneur.2018.00156. 29628907 PMC5877503

[jnc15516-bib-0180] Matthews, D. C. , Lerman, H. , Lukic, A. , Andrews, R. D. , Mirelman, A. , Wernick, M. N. , Giladi, N. , Strother, S. C. , Evans, K. C. , Cedarbaum, J. M. , & Even‐Sapir, E. (2018). FDG PET Parkinson’s disease‐related pattern as a biomarker for clinical trials in early stage disease. NeuroImage: Clinical, 20, 572–579. 10.1016/j.nicl.2018.08.006. 30186761 PMC6120603

[jnc15516-bib-0181] Matuskey, D. , Tinaz, S. , Wilcox, K. C. , Naganawa, M. , Toyonaga, T. , Dias, M. , Henry, S. , Pittman, B. , Ropchan, J. , Nabulsi, N. , Suridjan, I. , Comley, R. A. , Huang, Y. , Finnema, S. J. , & Carson, R. E. (2020). Synaptic changes in parkinson disease assessed with in vivo imaging. Annals of Neurology, 87, 329–338. 10.1002/ana.25682. 31953875 PMC7065227

[jnc15516-bib-0182] Maurer, A. , Leonov, A. , Ryazanov, S. , Herfert, K. , Kuebler, L. , Buss, S. , Schmidt, F. , Weckbecker, D. , Linder, R. , Bender, D. , Giese, A. , Pichler, B. J. , & Griesinger, C. (2020). ^11^C Radiolabeling of anle253b: a putative PET tracer for Parkinson's disease that binds to α‐synuclein fibrils in vitro and crosses the blood‐brain barrier. ChemMedChem, 15, 411–415. 10.1002/cmdc.201900689. 31859430 PMC7079211

[jnc15516-bib-0183] McCluskey, S. P. , Plisson, C. , Rabiner, E. A. , & Howes, O. (2020). Advances in CNS PET: The state‐of‐the‐art for new imaging targets for pathophysiology and drug development. European Journal of Nuclear Medicine and Molecular Imaging, 47, 451–489. 31541283 10.1007/s00259-019-04488-0 PMC6974496

[jnc15516-bib-0184] Meiser, J. , Weindl, D. , & Hiller, K. (2013). Complexity of dopamine metabolism. Cell Communication and Signaling, 11, 34. 23683503 10.1186/1478-811X-11-34 PMC3693914

[jnc15516-bib-0185] Meles, S. K. , Teune, L. K. , Jong, B. M. , de Dierckx, R. A. , & Leenders, K. L. (2017). Metabolic imaging in parkinson disease. Journal of Nuclear Medicine, 58, 23–28. 27879372 10.2967/jnumed.116.183152

[jnc15516-bib-0186] Melzer, T. R. , Watts, R. , MacAskill, M. R. , Pearson, J. F. , Rueger, S. , Pitcher, T. L. , Livingston, L. , Graham, C. , Keenan, R. , Shankaranarayanan, A. , Alsop, D. C. , Dalrymple‐Alford, J. C. , & Anderson, T. J. (2011). Arterial spin labelling reveals an abnormal cerebral perfusion pattern in Parkinson’s disease. Brain, 134, 845–855. 10.1093/brain/awq377. 21310726 PMC3105489

[jnc15516-bib-0187] Mertsalmi, T. H. , Aho, V. T. E. , Pereira, P. A. B. , Paulin, L. , Pekkonen, E. , Auvinen, P. , & Scheperjans, F. (2017). More than constipation – bowel symptoms in Parkinson’s disease and their connection to gut microbiota. European Journal of Neurology, 24, 1375–1383. 28891262 10.1111/ene.13398

[jnc15516-bib-0188] Meyer, P. M. , Strecker, K. , Kendziorra, K. , Becker, G. , Hesse, S. , Woelpl, D. , Hensel, A. , Patt, M. , Sorger, D. , Wegner, F. , Lobsien, D. , Barthel, H. , Brust, P. , Gertz, H. J. , Sabri, O. , & Schwarz, J. (2009). Reduced α4β2*–nicotinic acetylcholine receptor binding and its relationship to mild cognitive and depressive symptoms in Parkinson disease. Archives of General Psychiatry, 66, 866. 10.1001/archgenpsychiatry.2009.106. 19652126

[jnc15516-bib-0189] Mishina, M. , Ishiwata, K. , Naganawa, M. , Kimura, Y. , Kitamura, S. , Suzuki, M. , Hashimoto, M. , Ishibashi, K. , Oda, K. , Sakata, M. , Hamamoto, M. , Kobayashi, S. , Katayama, Y. , & Ishii, K. (2011). Adenosine A2A receptors measured with [^11^C]TMSX PET in the striata of Parkinson’s disease patients. PLoS One, 6, e17338. 10.1371/journal.pone.0017338. 21386999 PMC3046146

[jnc15516-bib-0190] Monahan, A. J. , Warren, M. , & Carvey, P. M. (2008). Neuroinflammation and peripheral immune infiltration in Parkinson’s disease: An autoimmune hypothesis. Cell Transplantation, 17, 363–372. 10.3727/096368908784423328. 18522239

[jnc15516-bib-0191] Moosa, S. , Martínez‐Fernández, R. , Elias, W. J. , del Alamo, M. , Eisenberg, H. M. , & Fishman, P. S. (2019). The role of high‐intensity focused ultrasound as a symptomatic treatment for Parkinson’s disease. Movement Disorders, 34, 1243–1251. 10.1002/mds.27779. 31291491

[jnc15516-bib-0192] Mori, W. , Yamasaki, T. , Hattori, Y. , Zhang, Y. , Kumata, K. , Fujinaga, M. , Hanyu, M. , Nengaki, N. , Zhang, H. , & Zhang, M.‐R. (2020). Radiosynthesis and evaluation of 4‐(6‐[^18^F]Fluoro‐4‐(5‐isopropoxy‐1H‐indazol‐3‐yl)pyridin‐2‐yl)morpholine as a novel radiotracer candidate targeting leucine‐rich repeat kinase 2. RSC Medicinal Chemistry, 11, 676–684. 33479667 10.1039/c9md00590k PMC7649847

[jnc15516-bib-0193] Morrish, P. K. , Rakshi, J. S. , Bailey, D. L. , Sawle, G. V. , & Brooks, D. J. (1998). Measuring the rate of progression and estimating the preclinical period of Parkinson’s disease with [^18^F]dopa PET. Journal of Neurology, Neurosurgery and Psychiatry, 64, 314–319. 9527140 10.1136/jnnp.64.3.314 PMC2170010

[jnc15516-bib-0194] Mukherjee, J. , Christian, B. T. , Dunigan, K. A. , Shi, B. , Narayanan, T. K. , Satter, M. , & Mantil, J. (2002). Brain imaging of ^18^F‐fallypride in normal volunteers: Blood analysis, distribution, test‐retest studies, and preliminary assessment of sensitivity to aging effects on dopamine D_2_/D_3_ receptors. Synapse (New York, N. Y.), 46, 170–188. 10.1002/syn.10128. 12325044

[jnc15516-bib-0195] Mukherjee, J. , Yang, Z.‐Y. , Brown, T. , Roemer, J. , & Cooper, M. (1996). ^18^F‐desmethoxyfallypride: A fluorine‐18 labeled radiotracer with properties similar to carbon‐11 raclopride for pet imaging studies of dopamine D_2_ receptors. Life Sciences, 59, 669–678. 10.1016/0024-3205(96)00348-7. 8761017

[jnc15516-bib-0196] Müller, M. L. T. M. , & Bohnen, N. I. (2013). Cholinergic dysfunction in Parkinson’s disease. Current Neurology and Neuroscience Reports, 13, 377. 23943367 10.1007/s11910-013-0377-9 PMC3991467

[jnc15516-bib-0197] Nahimi, A. , Kinnerup, M. B. , Sommerauer, M. , Gjedde, A. , & Borghammer, P. (2018). Molecular imaging of the noradrenergic system in idiopathic Parkinson’s disease. International Review of Neurobiology, 141, 251–274. 30314598 10.1016/bs.irn.2018.07.028

[jnc15516-bib-0198] Nahimi, A. , Sommerauer, M. , Kinnerup, M. B. , Østergaard, K. , Wintherdahl, M. , Jacobsen, J. , Schacht, A. , Johnsen, B. , Damholdt, M. F. , Borghammer, P. , & Gjedde, A. (2018). Noradrenergic deficits in Parkinson disease imaged with ^11^C‐MeNER. Journal of Nuclear Medicine, 59, 659–664. 10.2967/jnumed.117.190975. 28848039

[jnc15516-bib-0199] NDA 200655 for Fluorodopa F‐18 https://www.accessdata.fda.gov/scripts/cder/daf/index.cfm?event=overview.process&varApplNo=200655.

[jnc15516-bib-0200] Neumeyer, J. L. , Wang, S. Y. , Milius, R. A. , Baldwin, R. M. , Zea‐Ponce, Y. , Hoffer, P. B. , Sybirska, E. , Al‐Tikriti, M. , Charney, D. S. , & Malison, R. T. (1991). [^123^I]‐2 beta‐carbomethoxy‐3 beta‐(4‐iodophenyl)tropane: High‐affinity SPECT radiotracer of monoamine reuptake sites in brain. Journal of Medicinal Chemistry, 34, 3144–3146. 1920365 10.1021/jm00114a027

[jnc15516-bib-0201] Niccolini, F. , Foltynie, T. , Reis Marques, T. , Muhlert, N. , Tziortzi, A. C. , Searle, G. E. , Natesan, S. , Kapur, S. , Rabiner, E. A. , Gunn, R. N. , Piccini, P. , & Politis, M. (2015). Loss of phosphodiesterase 10A expression is associated with progression and severity in Parkinson’s disease. Brain, 138, 3003–3015. 10.1093/brain/awv219. 26210536

[jnc15516-bib-0202] Niccolini, F. , Rocchi, L. , & Politis, M. (2015). Molecular imaging of levodopa‐induced dyskinesias. Cellular and Molecular Life Sciences, 72, 2107–2117. 10.1007/s00018-015-1854-x. 25681866 PMC11113208

[jnc15516-bib-0203] Niccolini, F. , Wilson, H. , Pagano, G. , Coello, C. , Mehta, M. A. , Searle, G. E. , Gunn, R. N. , Rabiner, E. A. , Foltynie, T. , & Politis, M. (2017). Loss of phosphodiesterase 4 in Parkinson disease. Neurology, 89, 586–593. 10.1212/WNL.0000000000004201. 28701494

[jnc15516-bib-0204] Niethammer, M. , Feigin, A. , & Eidelberg, D. (2012). Functional neuroimaging in Parkinson’s disease. Cold Spring Harbor Perspectives in Medicine, 2, a009274. 10.1101/cshperspect.a009274. 22553499 PMC3331691

[jnc15516-bib-0205] Nors Pedersen, M. , Foderà, V. , Horvath, I. , van Maarschalkerweerd, A. , Nørgaard Toft, K. , Weise, C. , Almqvist, F. , Wolf‐Watz, M. , Wittung‐Stafshede, P. , & Vestergaard, B. (2015). Direct correlation between ligand‐induced α‐synuclein oligomers and amyloid‐like fibril growth. Scientific Reports, 5, 10422. 10.1038/srep10422. 26020724 PMC4603703

[jnc15516-bib-0206] Nutt, J. G. , Rufener, S. L. , Carter, J. H. , Anderson, V. C. , Pahwa, R. , Hammerstad, J. P. , & Burchiel, K. J. (2001). Interactions between deep brain stimulation and levodopa in Parkinson’s disease. Neurology, 57, 1835–1842. 10.1212/WNL.57.10.1835. 11723273

[jnc15516-bib-0207] Oertel, W. , & Schulz, J. B. (2016). Current and experimental treatments of Parkinson disease: A guide for neuroscientists. Journal of Neurochemistry, 139, 325–337. 10.1111/jnc.13750. 27577098

[jnc15516-bib-0208] Ohya, T. , Okamura, T. , Nagai, Y. , Fukushi, K. , Irie, T. , Suhara, T. , Zhang, M.‐R. , Fukumura, T. , & Kikuchi, T. (2011). Effect of radiolabeled metabolite elimination from the brain on the accuracy of cerebral enzyme activity estimation using positron emission tomography with substrate tracers. NeuroImage, 56, 1105–1110. 10.1016/j.neuroimage.2011.02.030. 21324368

[jnc15516-bib-0209] Okuzumi, A. , Hatano, T. , Kamagata, K. , Hori, M. , Mori, A. , Oji, Y. , Taniguchi, D. , Daida, K. , Shimo, Y. , Yanagisawa, N. , Nojiri, S. , Aoki, S. , & Hattori, N. (2019). Neuromelanin or DaT‐SPECT: which is the better marker for discriminating advanced Parkinson’s disease? European Journal of Neurology, 26, 1408–1416. 10.1111/ene.14009. 31136060 PMC6851628

[jnc15516-bib-0210] Oliveri, V. (2019). Toward the discovery and development of effective modulators of α‐synuclein amyloid aggregation. European Journal of Medicinal Chemistry, 167, 10–36. 30743095 10.1016/j.ejmech.2019.01.045

[jnc15516-bib-0211] Ono, M. , Doi, Y. , Watanabe, H. , Ihara, M. , Ozaki, A. , & Saji, H. (2016). Structure–activity relationships of radioiodinated diphenyl derivatives with different conjugated double bonds as ligands for α‐synuclein aggregates. RSC Advances, 6, 44305–44312. 10.1039/C6RA02710E.

[jnc15516-bib-0212] Orimo, S. , Suzuki, M. , Inaba, A. , & Mizusawa, H. (2012). ^123^I‐MIBG myocardial scintigraphy for differentiating Parkinson’s disease from other neurodegenerative parkinsonism: A systematic review and meta‐analysis. Parkinsonism & Related Disorders, 18, 494–500. 10.1016/j.parkreldis.2012.01.009. 22321865

[jnc15516-bib-0213] Otsuka, M. , Ichiya, Y. , Kuwabara, Y. , Hosokawa, S. , Sasaki, M. , Yoshida, T. , Fukumura, T. , Masuda, K. , & Kato, M. (1996). Differences in the reduced ^18^F‐Dopa uptakes of the caudate and the putamen in Parkinson’s disease: Correlations with the three main symptoms. Journal of the Neurological Sciences, 136, 169–173. 8815166 10.1016/0022-510x(95)00316-t

[jnc15516-bib-0214] Ouchi, Y. , Yoshikawa, E. , Sekine, Y. , Futatsubashi, M. , Kanno, T. , Ogusu, T. , & Torizuka, T. (2005). Microglial activation and dopamine terminal loss in early Parkinson’s disease. Annals of Neurology, 57, 168–175. 15668962 10.1002/ana.20338

[jnc15516-bib-0215] Pagano, G. , Niccolini, F. , Fusar‐Poli, P. , & Politis, M. (2017). Serotonin transporter in Parkinson’s disease: A meta‐analysis of positron emission tomography studies. Annals of Neurology, 81, 171–180. 10.1002/ana.24859. 28019672

[jnc15516-bib-0216] Pagano, G. , & Politis, M. (2018). Molecular imaging of the serotonergic system in Parkinson’s disease. International Review of Neurobiology, 141, 173–210. 30314596 10.1016/bs.irn.2018.08.002

[jnc15516-bib-0217] Pagano, G. , Rengo, G. , Pasqualetti, G. , Femminella, G. D. , Monzani, F. , Ferrara, N. , & Tagliati, M. (2015). Cholinesterase inhibitors for Parkinson’s disease: A systematic review and meta‐analysis. Journal of Neurology, Neurosurgery and Psychiatry, 86, 767–773. 25224676 10.1136/jnnp-2014-308764

[jnc15516-bib-0219] Park, E. (2012). A new era of clinical dopamine transporter imaging using ^123^I‐FP‐CIT. Journal of Nuclear Medicine Technology, 40, 222–228. 23160562 10.2967/jnmt.112.111617

[jnc15516-bib-0220] Park, G. , Tan, J. , Garcia, G. , Kang, Y. , Salvesen, G. , & Zhang, Z. (2016). Regulation of histone acetylation by autophagy in Parkinson disease. Journal of Biological Chemistry, 291, 3531–3540. 26699403 10.1074/jbc.M115.675488 PMC4751393

[jnc15516-bib-0221] Parkes, J. D. , Calver, D. M. , Zilkha, K. J. , & Knill‐Jonbs, R. P. (1970). Controlled trial of amantadine hydrochloride in Parkinson’s disease. Lancet, 295, 259–262. 10.1016/S0140-6736(70)90634-3. 4189290

[jnc15516-bib-0222] Parmar, M. , Grealish, S. , & Henchcliffe, C. (2020). The future of stem cell therapies for Parkinson disease. Nature Reviews Neuroscience, 21, 103–115. 31907406 10.1038/s41583-019-0257-7

[jnc15516-bib-0223] Pascoal, T. A. , Chamoun, M. , Shin, M. , Benedet, A. L. , Mathotaarachchi, S. , Kang, M. S. , Therriault, J. , Savard, M. , Thomas, E. , Massarweh, G. , Soucy, J.‐P. , Gauthier, S. , & Rosa‐Neto, P. (2018). Imaging epigenetics in the human brain with the novel [^11^C]Martinostat PET in preclinical AD, MCI, AD, and frontotemporal dementia individuals. Alzheimer's & Dementia, 14, P9–P10. 10.1016/j.jalz.2018.06.2053.

[jnc15516-bib-0224] Pasquini, J. , Ceravolo, R. , Brooks, D. J. , Bonuccelli, U. , & Pavese, N. (2020). Progressive loss of raphe nuclei serotonin transporter in early Parkinson’s disease: A longitudinal ^123^I‐FP‐CIT SPECT study. Parkinsonism & Related Disorders, 77, 170–175. 30981664 10.1016/j.parkreldis.2019.03.025

[jnc15516-bib-0225] Paterson, L. M. , Tyacke, R. J. , Nutt, D. J. , & Knudsen, G. M. (2010). Measuring endogenous 5‐HT release by emission tomography: Promises and pitfalls. Journal of Cerebral Blood Flow and Metabolism, 30, 1682–1706. 10.1038/jcbfm.2010.104. 20664611 PMC3023404

[jnc15516-bib-0226] Pavese, N. , Metta, V. , Bose, S. K. , Chaudhuri, K. R. , & Brooks, D. J. (2010). Fatigue in Parkinson’s disease is linked to striatal and limbic serotonergic dysfunction. Brain, 133, 3434–3443. 10.1093/brain/awq268. 20884645

[jnc15516-bib-0227] Pavese, N. , Simpson, B. S. , Metta, V. , Ramlackhansingh, A. , Chaudhuri, K. R. , & Brooks, D. J. (2012). [^18^F]FDOPA uptake in the raphe nuclei complex reflects serotonin transporter availability. A combined [^18^F]FDOPA and [^11^C]DASB PET study in Parkinson’s disease. NeuroImage, 59, 1080–1084. 10.1016/j.neuroimage.2011.09.034. 21963917

[jnc15516-bib-0228] Perani, D. , Iaccarino, L. , Jacobs, A. H. , Lammertsma, A. A. , Nordberg, A. , Windhorst, A. D. , Gerhard, A. , Winkeler, A. , Jacobs, A. H. , Gee, A. , Kuhnast, B. , Halldin, C. , Perani, D. , Brooks, D. , Rodriguez‐Vieitez, E. , Turkheimer, F. E. , López‐Picón, F. , Knudsen, G. M. , Vercouillie, J. , Moresco, R. M. , … Pappata, S. (2019). Application of advanced brain positron emission tomography–based molecular imaging for a biological framework in neurodegenerative proteinopathies. Alzheimer's & Dementia: Diagnosis, Assessment & Disease Monitoring, 11, 327–332. 10.1016/j.dadm.2019.02.004. PMC6505113 31080871

[jnc15516-bib-0229] Pérez‐Lohman, C. , Kerik, N. E. , Díaz‐Meneses, I. E. , Cervantes‐Arriaga, A. , & Rodríguez‐Violante, M. (2018). Diagnostic utility of [^11^C]DTBZ positron emission tomography in clinically uncertain parkinsonism: Experience of a single tertiary center. Revista De Investigacion Clinica, 70, 285–290. 10.24875/RIC.18002644. 30532098

[jnc15516-bib-0230] Perry, E. , Walker, M. , Grace, J. , & Perry, R. (1999). Acetylcholine in mind: A neurotransmitter correlate of consciousness? Trends in Neurosciences, 22, 273–280. 10354606 10.1016/s0166-2236(98)01361-7

[jnc15516-bib-0231] Peter, J. (2009). Medical imaging modalities — An introduction. In C. Sensen , & B. Hallgrímsson (Eds.), Advanced imaging in biology and medicine, (pp. 225–254). Springer. http://link.springer.com/10.1007/978‐3‐540‐68993‐5_10.

[jnc15516-bib-0232] Petrou, M. , Dwamena, B. A. , Foerster, B. R. , Maceachern, M. P. , Bohnen, N. I. , Müller, M. L. , Albin, R. L. , & Frey, K. A. (2015). Amyloid deposition in Parkinson’s disease and cognitive impairment: A systematic review. Movement Disorders, 30, 928–935. 10.1002/mds.26191. 25879534 PMC4478091

[jnc15516-bib-0233] Piccini, P. , Brooks, D. J. , Björklund, A. , Gunn, R. N. , Grasby, P. M. , Brundin, P. , Hagell, P. , Rehncrona, S. , Widner, H. , & Lindvall, O. (1999). Dopamine release from nigral transplants visualized in vivo in a Parkinson’s patient. Nature Neuroscience, 12, 1137–1140. 10.1038/16060 10570493

[jnc15516-bib-0234] Pinna, A. , Serra, M. , Morelli, M. , & Simola, N. (2018). Role of adenosine A_2A_ receptors in motor control: Relevance to Parkinson’s disease and dyskinesia. Journal of Neural Transmission, 125, 1273–1286. 10.1007/s00702-018-1848-6. 29396609

[jnc15516-bib-0235] Politis, M. , & Niccolini, F. (2015). Serotonin in Parkinson’s disease. Behavioral Brain Research, 277, 136–145. 10.1016/j.bbr.2014.07.037 25086269

[jnc15516-bib-0236] Politis, M. , Wu, K. , Loane, C. , Quinn, N. P. , Brooks, D. J. , Oertel, W. H. , Björklund, A. , Lindvall, O. , & Piccini, P. (2012). Serotonin neuron loss and nonmotor symptoms continue in Parkinson’s patients treated with dopamine grafts. Science Translational Medicine, 4, 1–11. 10.1126/scitranslmed.3003391 22491951

[jnc15516-bib-0237] Polymeropoulos, M. H. (1997). Mutation in the ‐synuclein gene identified in families with Parkinson’s disease. Science, 276, 2045–2047. 10.1126/science.276.5321.2045. 9197268

[jnc15516-bib-0238] Poorkaj, P. , Nutt, J. G. , James, D. , Gancher, S. , Bird, T. D. , Steinbart, E. , Schellenberg, G. D. , & Payami, H. (2004). parkin mutation analysis in clinic patients with early‐onset Parkinson’s disease. American Journal of Medical Genetics, 129A, 44–50. 10.1002/ajmg.a.30157. 15266615

[jnc15516-bib-0239] Prince, J. , & Links, J. (2014). Medical imaging signals and systems, 2nd ed. Pearson.

[jnc15516-bib-0240] Puñal‐Riobóo, J. , Serena‐Puig, A. , Varela‐Lema, L. , Álvarez‐Páez, A. M. , & Ruano‐Ravina, A. (2009). Clinical utility of ^18^F‐DOPA‐PET in movement disorders. A systematic review. Revista Española de Medicina Nuclear e Imagen Molecular (English Edition), 28, 106–113. 19558950

[jnc15516-bib-0241] Pyatigorskaya, N. , Gallea, C. , Garcia‐Lorenzo, D. , Vidailhet, M. , & Lehericy, S. (2014). A review of the use of magnetic resonance imaging in Parkinson’s disease. Therapeutic Advances in Neurological Disorders, 7, 206–220. 25002908 10.1177/1756285613511507 PMC4082302

[jnc15516-bib-0242] Pysz, M. A. , Gambhir, S. S. , & Willmann, J. K. (2010). Molecular imaging: Current status and emerging strategies. Clinical Radiology, 65, 500–516. 10.1016/j.crad.2010.03.011. 20541650 PMC3150531

[jnc15516-bib-0243] Qamhawi, Z. , Towey, D. , Shah, B. , Pagano, G. , Seibyl, J. , Marek, K. , Borghammer, P. , Brooks, D. J. , & Pavese, N. (2015). Clinical correlates of raphe serotonergic dysfunction in early Parkinson’s disease. Brain, 138, 2964–2973. 10.1093/brain/awv215. 26209314

[jnc15516-bib-0244] Rahmim, A. , & Zaidi, H. (2008). PET versus SPECT: strengths, limitations and challenges. Nuclear Medicine Communications, 29, 193–207. 18349789 10.1097/MNM.0b013e3282f3a515

[jnc15516-bib-0245] Rajput, A. H. (2001). Levodopa prolongs life expectancy and is non‐toxic to substantia nigra. Parkinsonism & Related Disorders, 8, 95–100. 11489674 10.1016/s1353-8020(01)00023-2

[jnc15516-bib-0246] Ramlackhansingh, A. F. , Bose, S. K. , Ahmed, I. , Turkheimer, F. E. , Pavese, N. , & Brooks, D. J. (2011). Adenosine 2A receptor availability in dyskinetic and nondyskinetic patients with Parkinson disease. Neurology, 76, 1811–1816. 10.1212/WNL.0b013e31821ccce4. 21606452 PMC3100120

[jnc15516-bib-0247] Remy, P. , Doder, M. , Lees, A. , Turjanski, N. , & Brooks, D. (2005). Depression in Parkinson’s disease: Loss of dopamine and noradrenaline innervation in the limbic system. Brain, 128, 1314–1322. 10.1093/brain/awh445. 15716302

[jnc15516-bib-0248] Rideout, H. J. , Chartier‐Harlin, M.‐C. , Fell, M. J. , Hirst, W. D. , Huntwork‐Rodriguez, S. , Leyns, C. E. G. , Mabrouk, O. S. , & Taymans, J.‐M. (2020). The current state‐of‐the Art of LRRK2‐based biomarker assay development in Parkinson’s disease. Frontiers in Neuroscience, 14, 865. 33013290 10.3389/fnins.2020.00865 PMC7461933

[jnc15516-bib-0249] Rinne, J. O. , Laihinen, A. , Någren, K. , Bergman, J. , Solin, O. , Haaparanta, M. , Ruotsalainen, U. , & Rinne, U. K. (1990). PET demonstrates different behaviour of striatal dopamine D_1_ and D_2_ receptors in early Parkinson’s disease. Journal of Neuroscience Research, 27, 494–499. 1981915 10.1002/jnr.490270409

[jnc15516-bib-0250] Rizzo, G. , Copetti, M. , Arcuti, S. , Martino, D. , Fontana, A. , & Logroscino, G. (2016). Accuracy of clinical diagnosis of Parkinson disease. Neurology, 86, 566–576. 10.1212/WNL.0000000000002350. 26764028

[jnc15516-bib-0251] Rossin, R. , Verkerk, P. R. , Bosch, S. M. , Den, V. , Vulders, R. C. M. , Verel, I. , Lub, J. , & Robillard, M. S. (2010). In vivo chemistry for pretargeted tumor imaging in live mice. Angewandte Chemie International Edition, 49, 3375–3378. 20391522 10.1002/anie.200906294

[jnc15516-bib-0252] Roussakis, A.‐A. , Piccini, P. , & Politis, M. (2013). Clinical utility of DaTscan^TM^ (^123^I‐Ioflupane Injection) in the diagnosis of parkinsonian syndromes. Degenerative Neurological and Neuromuscular Disease, 3, 33–39. 30890892 10.2147/DNND.S19807 PMC6065574

[jnc15516-bib-0253] Saeed, U. , Lang, A. E. , & Masellis, M. (2020). Neuroimaging advances in Parkinson’s disease and atypical parkinsonian syndromes. Frontiers in Neurology, 11, 572976. 33178113 10.3389/fneur.2020.572976 PMC7593544

[jnc15516-bib-0254] Sanders, L. H. , Laganière, J. , Cooper, O. , Mak, S. K. , Vu, B. J. , Huang, Y. A. , Paschon, D. E. , Vangipuram, M. , Sundararajan, R. , Urnov, F. D. , Langston, J. W. , Gregory, P. D. , Zhang, H. S. , Greenamyre, J. T. , Isacson, O. , & Schüle, B. (2014). LRRK2 mutations cause mitochondrial DNA damage in iPSC‐derived neural cells from Parkinson's disease patients: Reversal by gene correction. Neurobiology of Disease, 62, 381–386. 10.1016/j.nbd.2013.10.013. 24148854 PMC3877733

[jnc15516-bib-0255] Sasaki, T. , Ito, H. , Kimura, Y. , Arakawa, R. , Takano, H. , Seki, C. , Kodaka, F. , Fujie, S. , Takahata, K. , Nogami, T. , Suzuki, M. , Fujiwara, H. , Takahashi, H. , Nakao, R. , Fukumura, T. , Varrone, A. , Halldin, C. , Nishikawa, T. , & Suhara, T. (2012). Quantification of dopamine transporter in human brain using PET with ^18^F‐FE‐PE2I. Journal of Nuclear Medicine, 53, 1065–1073. 10.2967/jnumed.111.101626. 22689927

[jnc15516-bib-0256] Sasannezhad, P. , Juibary, A. G. , Sadri, K. , Sadeghi, R. , Sabour, M. , Kakhki, V. R. D. , & Alizadeh, H. (2017). ^99m^Tc‐TRODAT‐1 SPECT imaging in early and late onset Parkinson’s disease. Asia Ocean. Nuclear Medicine and Biology, 5, 114–119. 10.22038/aojnmb.2017.8844 PMC5482916 28660222

[jnc15516-bib-0257] Schapira, A. H. V. (2013). Recent developments in biomarkers in Parkinson disease. Current Opinion in Neurology, 26, 395–400. 23823465 10.1097/WCO.0b013e3283633741 PMC4196782

[jnc15516-bib-0258] Scheperjans, F. , Derkinderen, P. , & Borghammer, P. (2018). The gut and Parkinson’s disease: Hype or hope? Journal of Parkinson's Disease, 8, S31–S39. 10.3233/JPD-181477. PMC6311363 30584161

[jnc15516-bib-0259] Schildknecht, S. , Gerding, H. R. , Karreman, C. , Drescher, M. , Lashuel, H. A. , Outeiro, T. F. , Monte, D. A. , & Di, L. M. (2013). Oxidative and nitrative alpha‐synuclein modifications and proteostatic stress: Implications for disease mechanisms and interventions in synucleinopathies. Journal of Neurochemistry, 125, 491–511. 10.1111/jnc.12226. 23452040

[jnc15516-bib-0260] Schonhaut, D. R. , McMillan, C. T. , Spina, S. , Dickerson, B. C. , Siderowf, A. , Devous, M. D. , Tsai, R. , Winer, J. , Russell, D. S. , Litvan, I. , Roberson, E. D. , Seeley, W. W. , Grinberg, L. T. , Kramer, J. H. , Miller, B. L. , Pressman, P. , Nasrallah, I. , Baker, S. L. , Gomperts, S. N. , … Rabinovici, G. D. (2017). ^18^F‐flortaucipir tau positron emission tomography distinguishes established progressive supranuclear palsy from controls and Parkinson disease: A multicenter study. Annals of Neurology, 82, 622–634. 10.1002/ana.25060. 28980714 PMC5665658

[jnc15516-bib-0261] Schreckenberger, M. , Hägele, S. , Siessmeier, T. , Buchholz, H. G. , Armbrust‐Henrich, H. , Rösch, F. , Gründer, G. , Bartenstein, P. , & Vogt, T. (2004). The dopamine D2 receptor ligand ^18^F‐desmethoxyfallypride: An appropriate fluorinated PET tracer for the differential diagnosis of parkinsonism. European Journal of Nuclear Medicine and Molecular Imaging, 31, 1128–1135. 15042325 10.1007/s00259-004-1465-5

[jnc15516-bib-0262] Schulz‐Schaeffer, W. J. (2010). The synaptic pathology of α‐synuclein aggregation in dementia with Lewy bodies, Parkinson’s disease and Parkinson’s disease dementia. Acta Neuropathologica, 120, 131–143. 10.1007/s00401-010-0711-0. 20563819 PMC2892607

[jnc15516-bib-0263] Sehlin, D. , Fang, X. T. , Cato, L. , Antoni, G. , Lannfelt, L. , & Syvänen, S. (2016). Antibody‐based PET imaging of amyloid beta in mouse models of Alzheimer’s disease. Nature Communications, 7, 1–11. 10.1038/ncomms10759 PMC4762893 26892305

[jnc15516-bib-0264] Sehlin, D. , & Syvänen, S. (2019). Engineered antibodies: New possibilities for brain PET? European Journal of Nuclear Medicine and Molecular Imaging, 46, 2848–2858. 31342134 10.1007/s00259-019-04426-0 PMC6879437

[jnc15516-bib-0265] Seibyl, J. P. , Marchek, K. L. , Quinlan, D. , Sheff, K. , Zoghbi, S. , Zea‐Ponce, Y. , Baldwin, R. M. , Fussell, B. , Smith, E. O. , Charney, D. S. , Hoffer, P. B. , & Innis, R. B. (1995). Decreased single‐photon emission computed tomographic [^123^I]beta‐CIT striatal uptake correlates with symptom severity in parkinson's disease. Annals of Neurology, 38, 589–598. 10.1002/ana.410380407. 7574455

[jnc15516-bib-0266] Seibyl, J. P. , Marek, K. , Sheff, K. , Zoghbi, S. , Baldwin, R. M. , Charney, D. S. , Dyck, C. H. , & van Innis, R. B. (1998). Iodine‐123‐beta‐CIT and iodine‐123‐FPCIT SPECT measurement of dopamine transporters in healthy subjects and Parkinson’s patients. Journal of Nuclear Medicine, 39, 1500–1508. 9744331

[jnc15516-bib-0267] Shalgunov, V. , Xiong, M. , L’Estrade, E. T. , Raval, N. R. , Andersen, I. V. , Edgar, F. G. , Speth, N. R. , Baerentzen, S. L. , Hansen, H. D. , Donovan, L. L. , Nasser, A. , Peitersen, S. T. , Kjaer, A. , Knudsen, G. M. , Syvänen, S. , Palner, M. , & Herth, M. M. (2020). Blocking of efflux transporters in rats improves translational validation of brain radioligands. EJNMMI Research, 10, 124. 10.1186/s13550-020-00718-x. 33074370 PMC7572968

[jnc15516-bib-0268] Shinotoh, H. , Hirano, S. , & Shimada, H. (2021). PET imaging of acetylcholinesterase, in *PET SPECT* . Neurobiology of Disease, 19, 193–220.

[jnc15516-bib-0269] Shinotoh, H. , Namba, H. , Yamaguchi, M. , Fukushi, K. , Nagatsuka, S.‐I. , Iyo, M. , Asahina, M. , Hattori, T. , Tanada, S. , & Irie, T. (1999). Positron emission tomographic measurement of acetylcholinesterase activity reveals differential loss of ascending cholinergic systems in Parkinson’s disease and progressive supranuclear palsy. Annals of Neurology, 46, 62–69. 10.1002/1531-8249(199907)46:1<62:AID-ANA10>3.0.CO;2-P. 10401781

[jnc15516-bib-0270] Shotbolt, P. , Tziortzi, A. C. , Searle, G. E. , Colasanti, A. , van der Aart, J. , Abanades, S. , Plisson, C. , Miller, S. R. , Huiban, M. , Beaver, J. D. , Gunn, R. N. , Laruelle, M. , & Rabiner, E. A. (2012). Within‐subject comparison of [^11^C]‐( + )‐PHNO and [^11^C]raclopride sensitivity to acute amphetamine challenge in healthy humans. Journal of Cerebral Blood Flow & Metabolism, 32, 127–136. 10.1038/jcbfm.2011.115. 21878947 PMC3323295

[jnc15516-bib-0271] Sieradzan, K. A. , Fox, S. H. , Hill, M. , Dick, J. P. R. , Crossman, A. R. , & Brotchie, J. M. (2001). Cannabinoids reduce levodopa‐induced dyskinesia in Parkinson’s disease: A pilot study. Neurology, 57, 2108–2111. 10.1212/WNL.57.11.2108. 11739835

[jnc15516-bib-0272] Silverdale, M. A. , Nicholson, S. L. , Ravenscroft, P. , Crossman, A. R. , Millan, M. J. , & Brotchie, J. M. (2004). Selective blockade of D_3_ dopamine receptors enhances the anti‐parkinsonian properties of ropinirole and levodopa in the MPTP‐lesioned primate. Experimental Neurology, 188, 128–138. 10.1016/j.expneurol.2004.03.022. 15191809

[jnc15516-bib-0273] Singleton, A. B. , Farrer, M. J. , & Bonifati, V. (2013). The genetics of Parkinson’s disease: Progress and therapeutic implications. Movement Disorders, 28, 14–23. 10.1002/mds.25249. 23389780 PMC3578399

[jnc15516-bib-0274] Smith, R. , Schöll, M. , Londos, E. , Ohlsson, T. , & Hansson, O. (2018). ^18^F‐AV‐1451 in Parkinson’s disease with and without dementia and in dementia with lewy bodies. Scientific Reports, 8, 4717. 29549278 10.1038/s41598-018-23041-x PMC5856779

[jnc15516-bib-0275] Sommerauer, C. , Rebernik, P. , Reither, H. , Nanoff, C. , & Pifl, C. (2012). The noradrenaline transporter as site of action for the anti‐Parkinson drug amantadine. Neuropharmacology, 62, 1708–1716. 10.1016/j.neuropharm.2011.11.017. 22155208

[jnc15516-bib-0276] Sommerauer, M. , Fedorova, T. D. , Hansen, A. K. , Knudsen, K. , Otto, M. , Jeppesen, J. , Frederiksen, Y. , Blicher, J. U. , Geday, J. , Nahimi, A. , Damholdt, M. F. , Brooks, D. J. , & Borghammer, P. (2018). Evaluation of the noradrenergic system in Parkinson’s disease: An ^11^C‐MeNER PET and neuromelanin MRI study. Brain, 141, 496–504. 10.1093/brain/awx348. 29272343

[jnc15516-bib-0277] Sood, A. , Shukla, J. , Shree, R. , Vatsa, R. , Modi, M. , & Mittal, B. R. (2021). Comparative performance of ^99m^Tc‐TRODAT‐1 SPECT/CT and ^18^F‐FDOPA PET/CT imaging in patients with Parkinson’s disease, Parkinson‐plus syndrome, and essential tremor. Clinical Nuclear Medicine, 46, 95–102. 33234920 10.1097/RLU.0000000000003409

[jnc15516-bib-0278] Stéen, E. J. L. , Edem, P. E. , Nørregaard, K. , Jørgensen, J. T. , Shalgunov, V. , Kjaer, A. , & Herth, M. M. (2018). Pretargeting in nuclear imaging and radionuclide therapy: Improving efficacy of theranostics and nanomedicines. Biomaterials, 179, 209–245. 10.1016/j.biomaterials.2018.06.021. 30007471

[jnc15516-bib-0279] Strafella, A. P. , Bohnen, N. I. , Perlmutter, J. S. , Eidelberg, D. , Pavese, N. , Van Eimeren, T. , Piccini, P. , Politis, M. , Thobois, S. , Ceravolo, R. , Higuchi, M. , Kaasinen, V. , Masellis, M. , Peralta, M. C. , Obeso, I. , Pineda‐Pardo, J. Á. , Cilia, R. , Ballanger, B. , Niethammer, M. , & Stoessl, J. A. (2017). Molecular imaging to track Parkinson’s disease and atypical parkinsonisms: New imaging frontiers. Movement Disorders, 32, 181–192. 10.1002/mds.26907. 28150432

[jnc15516-bib-0280] Stuendl, A. , Kunadt, M. , Kruse, N. , Bartels, C. , Moebius, W. , Danzer, K. M. , Mollenhauer, B. , & Schneider, A. (2016). Induction of α‐synuclein aggregate formation by CSF exosomes from patients with Parkinson’s disease and dementia with Lewy bodies. Brain, 139, 481–494. 10.1093/brain/awv346. 26647156 PMC4805087

[jnc15516-bib-0281] Sulzer, D. , Cassidy, C. , Horga, G. , Kang, U. J. , Fahn, S. , Casella, L. , Pezzoli, G. , Langley, J. , Hu, X. P. , Zucca, F. A. , Isaias, I. U. , & Zecca, L. (2018). Neuromelanin detection by magnetic resonance imaging (MRI) and its promise as a biomarker for Parkinson’s disease. npj Parkinson's Disease, 4, 11. 10.1038/s41531-018-0047-3. PMC5893576 29644335

[jnc15516-bib-0282] Syvänen, S. , Lindhe, Ö. , Palner, M. , Kornum, B. R. , Rahman, O. , Långström, B. , Knudsen, G. M. , & Hammarlund‐Udenaes, M. (2009). Species differences in blood‐brain barrier transport of three positron emission tomography radioligands with emphasis on P‐glycoprotein transport. Drusg Metabolism and Disposition, 37, 635–643. 10.1124/dmd.108.024745. 19047468

[jnc15516-bib-0283] Tago, T. , & Toyohara, J. (2018). Advances in the development of PET ligands targeting histone deacetylases for the assessment of neurodegenerative diseases. Molecules, 23, 300. 10.3390/molecules23020300. PMC6017260 29385079

[jnc15516-bib-0284] Tang, C. C. , Poston, K. L. , Eckert, T. , Feigin, A. , Frucht, S. , Gudesblatt, M. , Dhawan, V. , Lesser, M. , Vonsattel, J.‐P. , Fahn, S. , & Eidelberg, D. (2010). Differential diagnosis of parkinsonism: A metabolic imaging study using pattern analysis. The Lancet Neurology, 9, 149–158. 10.1016/S1474-4422(10)70002-8. 20061183 PMC4617666

[jnc15516-bib-0285] Tanner, C. M. , Kamel, F. , Ross, G. W. , Hoppin, J. A. , Goldman, S. M. , Korell, M. , Marras, C. , Bhudhikanok, G. S. , Kasten, M. , Chade, A. R. , Comyns, K. , Richards, M. B. , Meng, C. , Priestley, B. , Fernandez, H. H. , Cambi, F. , Umbach, D. M. , Blair, A. , Sandler, D. P. , & Langston, J. W. (2011). Rotenone, paraquat, and Parkinson’s disease. Environmental Health Perspectives, 119, 866–872. 10.1289/ehp.1002839. 21269927 PMC3114824

[jnc15516-bib-0286] Tansey, M. G. , & Goldberg, M. S. (2010). Neuroinflammation in Parkinson’s disease: Its role in neuronal death and implications for therapeutic intervention. Neurobiology of Diseases, 37, 510–518. 10.1016/j.nbd.2009.11.004 PMC2823829 19913097

[jnc15516-bib-0287] Tatsumi, M. , Groshan, K. , Blakely, R. D. , & Richelson, E. (1997). Pharmacological profile of antidepressants and related compounds at human monoamine transporters. European Journal of Pharmacology, 340, 249–258. 9537821 10.1016/s0014-2999(97)01393-9

[jnc15516-bib-0288] Teune, L. K. , Renken, R. J. , Mudali, D. , Jong, B. M. , De, D. R. A. , Roerdink, J. B. T. M. , & Leenders, K. L. (2013). Validation of parkinsonian disease‐related metabolic brain patterns. Movement Disorders, 28, 547–551. 10.1002/mds.25361. 23483593

[jnc15516-bib-0289] Thinakaran, G. , & Koo, E. H. (2008). Amyloid precursor protein trafficking, processing, and function. Journal of Biological Chemistry, 283, 29615–29619. 18650430 10.1074/jbc.R800019200 PMC2573065

[jnc15516-bib-0290] Thobois, S. , Ardouin, C. , Lhommee, E. , Klinger, H. , Lagrange, C. , Xie, J. , Fraix, V. , Coelho Braga, M. C. , Hassani, R. , Kistner, A. , Juphard, A. , Seigneuret, E. , Chabardes, S. , Mertens, P. , Polo, G. , Reilhac, A. , Costes, N. , LeBars, D. , Savasta, M. , … Krack, P. (2010). Non‐motor dopamine withdrawal syndrome after surgery for Parkinson’s disease: Predictors and underlying mesolimbic denervation. Brain, 133, 1111–1127. 10.1093/brain/awq032. 20237128

[jnc15516-bib-0291] Thomas, B. , & Beal, M. F. (2007). Parkinson’s disease. Human Molecular Genetics, 16, 183–194. 10.1093/hmg/ddm159 17911161

[jnc15516-bib-0292] Toch, S.‐R. , Poussier, S. , Micard, E. , Bertaux, M. , Gucht, A. , Der, V. , Chevalier, E. , Marie, P.‐Y. , Guedj, E. , & Verger, A. (2019). Physiological whole‐brain distribution of [^18^F]FDOPA uptake index in relation to age and gender: Results from a voxel‐based semi‐quantitative analysis. Molecular Imaging and Biology, 21, 549–557. 10.1007/s11307-018-1256-1. 30073569

[jnc15516-bib-0293] Tolosa, E. , Vila, M. , Klein, C. , & Rascol, O. (2020). LRRK2 in Parkinson disease: Challenges of clinical trials. Nature Reviews. Neurology, 16, 97–107. 31980808 10.1038/s41582-019-0301-2

[jnc15516-bib-0294] Tran, J. , Anastacio, H. , & Bardy, C. (2020). Genetic predispositions of Parkinson’s disease revealed in patient‐derived brain cells. NPJ Parkinson's Disease, 6, 8. 10.1038/s41531-020-0110-8 PMC7181694 32352027

[jnc15516-bib-0295] Travin, M. I. , Matsunari, I. , Thomas, G. S. , Nakajima, K. , & Yoshinaga, K. (2019). How do we establish cardiac sympathetic nervous system imaging with ^123^I‐mIBG in clinical practice? Perspectives and lessons from Japan and the US. Journal of Nuclear Cardiology, 5, 5–20. 10.1007/s12350-018-1394-5. 30178272

[jnc15516-bib-0296] Tronel, C. , Largeau, B. , Ribeiro, M. J. S. , Guilloteau, D. , Dupont, A. C. , & Arlicot, N. (2017). Molecular targets for PET imaging of activated microglia: The current situation and future expectations. International Journal of Molecular Sciences, 18, 802. 10.3390/ijms18040802 PMC5412386 28398245

[jnc15516-bib-0297] Tseng, C.‐E. J. , Gilbert, T. M. , Catanese, M. C. , Hightower, B. G. , Peters, A. T. , Parmar, A. J. , Kim, M. , Wang, C. , Roffman, J. L. , Brown, H. E. , Perlis, R. H. , Zürcher, N. R. , & Hooker, J. M. (2020). In vivo human brain expression of histone deacetylases in bipolar disorder. Translational Psychiatry, 10, 224. 10.1038/s41398-020-00911-5. 32641695 PMC7343804

[jnc15516-bib-0298] Tsukada, H. , Kanazawa, M. , Ohba, H. , Nishiyama, S. , Harada, N. , & Kakiuchi, T. (2016). PET imaging of mitochondrial complex I with ^18^F‐BCPP‐EF in brain of Parkinsons disease model monkey. Journal of Nuclear Medicine, 57, 950–953. 26912430 10.2967/jnumed.115.169615

[jnc15516-bib-0299] Uzuegbunam, B. C. , Librizzi, D. , & Hooshyar, Y. B. (2020). PET radiopharmaceuticals for Alzheimer’s disease and Parkinson’s disease diagnosis, the current and future landscape. Molecules, 25, 977. 10.3390/molecules25040977. PMC7070523 32098280

[jnc15516-bib-0300] Valente, E. M. (2004). Hereditary early‐onset Parkinson’s disease caused by mutations in PINK1. Science, 304, 1158–1160. 10.1126/science.1096284. 15087508

[jnc15516-bib-0301] Vallone, D. , Picetti, R. , & Borrelli, E. (2000). Structure and function of dopamine receptors. Neuroscience and Biobehavioral Reviews, 24, 125–132. 10654668 10.1016/s0149-7634(99)00063-9

[jnc15516-bib-0302] Van, L. K. , Casteels, C. , Lunskens, S. , Goffin, K. , Grachev, I. D. , Bormans, G. , & Vandenberghe, W. (2012). Regional changes in type 1 cannabinoid receptor availability in Parkinson’s disease in vivo. Neurobiology of Aging, 33, 1–8. 21459482 10.1016/j.neurobiolaging.2011.02.009

[jnc15516-bib-0303] Vaquer‐Alicea, J. , & Diamond, M. I. (2019). Propagation of protein aggregation in neurodegenerative diseases. Annual Review of Biochemistry, 88, 785–810. 10.1146/annurev-biochem-061516-045049 30917002

[jnc15516-bib-0304] Varnäs, K. , Cselényi, Z. , Jucaite, A. , Halldin, C. , Svenningsson, P. , Farde, L. , & Varrone, A. (2019). PET imaging of [^11^C]PBR28 in Parkinson’s disease patients does not indicate increased binding to TSPO despite reduced dopamine transporter binding. European Journal of Nuclear Medicine and Molecular Imaging, 46, 367–375. 30270409 10.1007/s00259-018-4161-6 PMC6333720

[jnc15516-bib-0305] Varrone, A. , Svenningsson, P. , Forsberg, A. , Varnäs, K. , Tiger, M. , Nakao, R. , Halldin, C. , Nilsson, L.‐G. , & Farde, L. (2014). Positron emission tomography imaging of 5‐hydroxytryptamine 1B receptors in Parkinson’s disease. Neurobiology of Aging, 35, 867–875. 24126162 10.1016/j.neurobiolaging.2013.08.025

[jnc15516-bib-0306] Varrone, A. , Svenningsson, P. , Marklund, P. , Fatouros‐Bergman, H. , Forsberg, A. , Halldin, C. , Nilsson, L.‐G. , & Farde, L. (2015). 5‐HT 1B receptor imaging and cognition: A positron emission tomography study in control subjects and Parkinson’s disease patients. Synapse (New York, N. Y.), 69, 365–374. 10.1002/syn.21823 25914348

[jnc15516-bib-0307] Vila, M. (2019). Neuromelanin, aging, and neuronal vulnerability in Parkinson’s disease. Movement Disorders, 34, 1440–1451. 10.1002/mds.27776. 31251435 PMC7079126

[jnc15516-bib-0308] Visser, A. K. D. , Waarde, A. , & van Willemsen, A. T. M. , Bosker, F. J. , Luiten, P. G. M. , Boer, J. A , den Kema, I. P. , & Dierckx, R. A. J. O. (2011). Measuring serotonin synthesis: From conventional methods to PET tracers and their (pre)clinical implications. European Journal of Nuclear Medicine and Molecular Imaging, 38, 576–591. 21113591 10.1007/s00259-010-1663-2 PMC3034914

[jnc15516-bib-0309] Vlaar, A. M. M. , Nijs, T. , de Kessels, A. G. H. , Vreeling, F. W. , Winogrodzka, A. , Mess, W. H. , Tromp, S. C. , & Kroonenburgh, M. J. P. G. , & van Weber, W. E. J. (2008). Diagnostic value of ^123^I‐Ioflupane and ^123^I‐Iodobenzamide SPECT scans in 248 patients with parkinsonian syndromes. European Neurology, 59, 258–266. 18264015 10.1159/000115640

[jnc15516-bib-0310] Vo, A. , Sako, W. , Fujita, K. , Peng, S. , Mattis, P. J. , Skidmore, F. M. , Ma, Y. , Uluğ, A. M. , & Eidelberg, D. (2017). Parkinson’s disease‐related network topographies characterized with resting state functional MRI. Human Brain Mapping, 38, 617–630. 10.1002/hbm.23260. 27207613 PMC5118197

[jnc15516-bib-0311] Von, C. R. , & Shulman, L. M. (2016). Clinical subtypes and genetic heterogeneity: Of lumping and splitting in Parkinson disease. Current Opinion in Neurology, 29, 727–734. 10.1097/WCO.0000000000000384. 27749396

[jnc15516-bib-0312] Vriend, C. , Raijmakers, P. , Veltman, D. J. , Dijk, K. D. , Van, W. Y. D. , Der, V. , Foncke, E. M. J. , Smit, J. H. , Berendse, H. W. , & Van Den, H. O. A. (2014). Depressive symptoms in Parkinson’s disease are related to reduced [^123^I]FP‐CIT binding in the caudate nucleus. Journal of Neurology, Neurosurgery and Psychiatry, 85, 159–164. 23813742 10.1136/jnnp-2012-304811

[jnc15516-bib-0313] Walker, Z. , Gandolfo, F. , Orini, S. , Garibotto, V. , Agosta, F. , Arbizu, J. , Bouwman, F. , Drzezga, A. , Nestor, P. , Boccardi, M. , Altomare, D. , Festari, C. , & Nobili, F. (2018). Clinical utility of FDG PET in Parkinson’s disease and atypical parkinsonism associated with dementia. European Journal of Nuclear Medicine and Molecular Imaging, 45, 1534–1545. 10.1007/s00259-018-4031-2. 29779045 PMC6061481

[jnc15516-bib-0314] Wang, M. , Gao, M. , Xu, Z. , & Zheng, Q.‐H. (2017). Synthesis of [^11^C]HG‐10‐102‐01 as a new potential PET agent for imaging of LRRK2 enzyme in Parkinson’s disease. Bioorganic & Medicinal Chemistry Letters, 27, 1351–1355. 10.1016/j.bmcl.2017.02.019. 28223019

[jnc15516-bib-0315] Wanneveich, M. , Moisan, F. , Jacqmin‐Gadda, H. , Elbaz, A. , & Joly, P. (2018). Projections of prevalence, lifetime risk, and life expectancy of Parkinson’s disease (2010–2030) in France. Movement Disorders, 33, 1449–1455. 10.1002/mds.27447. 30145805

[jnc15516-bib-0316] Watabe, T. , & Hatazawa, J. (2019). Evaluation of functional connectivity in the brain using positron emission tomography: A mini‐review. Frontiers in Neuroscience, 13, 775. 31402852 10.3389/fnins.2019.00775 PMC6676772

[jnc15516-bib-0317] Weingarten, C. P. , Sundman, M. H. , Hickey, P. , & Chen, N. (2015). Neuroimaging of Parkinson’s disease: Expanding views. Neuroscience and Biobehavioral Reviews, 59, 16–52. 26409344 10.1016/j.neubiorev.2015.09.007 PMC4763948

[jnc15516-bib-0318] Wey, H. Y. , Gilbert, T. M. , Zürcher, N. R. , She, A. , Bhanot, A. , Taillon, B. D. , Schroeder, F. A. , Wang, C. , Haggarty, S. J. , & Hooker, J. M. (2016). Insights into neuroepigenetics through human histone deacetylase PET imaging. Science Translational Medicine, 8, 351ra106. 10.1126/scitranslmed.aaf7551. PMC5784409 27510902

[jnc15516-bib-0320] Wielepp, J. P. , Burgunder, J. M. , Pohle, T. , Ritter, E. P. , Kinser, J. A. , & Krauss, J. K. (2001). Deactivation of thalamocortical activity is responsible for suppression of parkinsonian tremor by thalamic stimulation: A ^99m^Tc‐ECD SPECT study. Clinical Neurology and Neurosurgery, 103, 228–231. 10.1016/S0303-8467(01)00165-2. 11714567

[jnc15516-bib-0321] Williams, D. R. , & Lees, A. J. (2009). Progressive supranuclear palsy: Clinicopathological concepts and diagnostic challenges. The Lancet Neurology, 8, 270–279. 10.1016/S1474-4422(09)70042-0. 19233037

[jnc15516-bib-0322] Wilson, H. , Politis, M. , Rabiner, E. A. , & Middleton, L. T. (2020). Novel PET biomarkers to disentangle molecular pathways across age‐related neurodegenerative diseases. Cells, 9, 2581. 10.3390/cells9122581. PMC7761606 33276490

[jnc15516-bib-0323] Winer, J. R. , Maass, A. , Pressman, P. , Stiver, J. , Schonhaut, D. R. , Baker, S. L. , Kramer, J. , Rabinovici, G. D. , & Jagust, W. J. (2018). Associations between Tau, β‐amyloid, and cognition in Parkinson disease. JAMA Neurology, 75, 227–235. 10.1001/jamaneurol.2017.3713. 29228071 PMC5838622

[jnc15516-bib-0324] Winogrodzka, A. (2003). [^123^I]beta‐CIT SPECT is a useful method for monitoring dopaminergic degeneration in early stage Parkinson’s disease. Journal of Neurology, Neurosurgery and Psychiatry, 74, 294–298. 12588911 10.1136/jnnp.74.3.294 PMC1738309

[jnc15516-bib-0325] Wong, K. K. , Raffel, D. M. , Bohnen, N. I. , Altinok, G. , Gilman, S. , & Frey, K. A. (2017). 2‐year natural decline of cardiac sympathetic innervation in idiopathic parkinson disease studied with ^11^C‐hydroxyephedrine PET. Journal of Nuclear Medicine, 58, 326–331. 27539837 10.2967/jnumed.116.176891 PMC5288743

[jnc15516-bib-0326] Wu, T. , Ma, Y. , Zheng, Z. , Peng, S. , Wu, X. , Eidelberg, D. , & Chan, P. (2015). Parkinson’s disease‐related spatial covariance pattern identified with resting‐state functional MRI. Journal of Cerebral Blood Flow and Metabolism, 35, 1764–1770. 10.1038/jcbfm.2015.118. 26036935 PMC4635231

[jnc15516-bib-0327] Zee, S. , Vállez, G. D. , Elsinga, P. H. , Willemsen, A. T. M. , Boersma, H. H. , Gerritsen, M. J. J. , Spikman, J. M. , & Laar, T. (2019). [^18^F]Fluoroethoxybenzovesamicol in Parkinson’s disease patients: Quantification of a novel cholinergic positron emission tomography tracer. Movement Disorders, 34, 924–926. 30977934 10.1002/mds.27698

[jnc15516-bib-0328] Zeglis, B. M. , Sevak, K. K. , Reiner, T. , Mohindra, P. , Carlin, S. D. , Zanzonico, P. , Weissleder, R. , & Lewis, J. S. (2013). A Pretargeted PET imaging strategy based on bioorthogonal diels‐alder click chemistry. Journal of Nuclear Medicine, 54, 1389–1396. 23708196 10.2967/jnumed.112.115840 PMC4151562

[jnc15516-bib-0329] Zella, S. M. A. , Metzdorf, J. , Ciftci, E. , Ostendorf, F. , Muhlack, S. , Gold, R. , & Tönges, L. (2019). Emerging immunotherapies for Parkinson disease. Neurology and Therapy, 8, 29–44. 30539376 10.1007/s40120-018-0122-z PMC6534677

[jnc15516-bib-0330] Zhang, J. , & Tan, L.‐ C.‐S. (2016). Revisiting the medical management of Parkinson’s disease: Levodopa versus dopamine agonist. Current Neuropharmacology, 14, 356–363. 10.2174/1570159X14666151208114634. 26644151 PMC4876591

[jnc15516-bib-0331] Zhang, X. , Gao, F. , Wang, D. , Li, C. , Fu, Y. , He, W. , & Zhang, J. (2018). Tau pathology in Parkinson’s disease. Frontiers in Neurology, 9, 1–7. 30333786 10.3389/fneur.2018.00809 PMC6176019

[jnc15516-bib-0332] Zhang, X. , Jin, H. , Padakanti, P. , Li, J. , Yang, H. , Fan, J. , Mach, R. , Kotzbauer, P. , & Tu, Z. (2014). Radiosynthesis and in vivo evaluation of two PET radioligands for imaging α‐synuclein. Applied Sciences, 4, 66–78. 25642331 10.3390/app4010066 PMC4310556

[jnc15516-bib-0333] Ziebell, M. , Holm‐Hansen, S. , Thomsen, G. , Wagner, A. , Jensen, P. , Pinborg, L. H. , & Knudsen, G. M. (2010). Serotonin transporters in dopamine transporter imaging: A head‐to‐head comparison of dopamine transporter SPECT radioligands ^123^I‐FP‐CIT and ^123^I‐PE2I. Journal of Nuclear Medicine, 51, 1885–1891. 21078806 10.2967/jnumed.110.078337

[jnc15516-bib-0334] Zimprich, A. , Biskup, S. , Leitner, P. , Lichtner, P. , Farrer, M. , Lincoln, S. , Kachergus, J. , Hulihan, M. , Uitti, R. J. , Calne, D. B. , Stoessl, A. J. , Pfeiffer, R. F. , Patenge, N. , Carbajal, I. C. , Vieregge, P. , Asmus, F. , Müller‐Myhsok, B. , Dickson, D. W. , Meitinger, T. , … Gasser, T. (2004). Mutations in LRRK2 cause autosomal‐dominant parkinsonism with pleomorphic pathology. Neuron, 44, 601–607. 10.1016/j.neuron.2004.11.005. 15541309

